# Supplementary material for: Sex and smoking bias in the selection of somatic mutations in human bladder
Source: Nature. 2025 Oct 8;647(8089):436–44. doi: 10.1038/s41586-025-09521-x (PMC12611770; doi:10.1038/s41586-025-09521-x)
Supplement: Supplementary file 1 — Supplementary Figs. 1–3, Notes 1–12, Tables 8–10 and References. [file 41586_2025_9521_MOESM1_ESM.pdf]

nature portfolio

https://doi.org/10.1038/s41586-025-09521-x

Supplementary information

Sex and smoking bias in the selection of somatic mutations in human bladder

In the format provided by the authors and unedited

Supplementary Material

Sex and Smoking Bias in the Selection of Somatic Mutations in Human Bladder

Ferriol Calvet[1,4,6], Raquel Blanco Martinez-Illescas[1,4,6], Ferran Muiños[1,4], Maria Tretiakova[2], Elena S. Latorre-Esteves[2], Jeanne Fredrickson[2], Maria Andrianova[1], Stefano Pellegrini[1,4], Axel Rosendahl Huber[1], Joan Enric Ramis-Zaldivar[1,4], Shuyi (Charlotte) An[2], Elana Thieme[2], Brendan F. Kohrn[2], Miguel Grau[1], Abel Gonzalez-Perez[1,4,5,7], Nuria Lopez-Bigas[1,3,4,5,7,@], Rosa Ana Risques[2,7,@]

[6] These authors contributed equally and the order was decided randomly: R. Blanco Martinez-Illescas, F. Calvet

[7] These authors jointly supervised this work: A. Gonzalez-Perez, N. Lopez-Bigas, R. Risques

[@] Correspondence should be addressed to Nuria Lopez-Bigas <nuria.lopez@irbbarcelona.org> and Rosa Ana Risques <rrisques@uw.edu>

Affiliations

1. Institute for Research in Biomedicine (IRB Barcelona), The Barcelona Institute of Science and Technology, Baldiri Reixac, 10, 08028 Barcelona, Spain.
2. Department of Laboratory Medicine and Pathology, University of Washington, Seattle, WA.
3. Institució Catalana de Recerca i Estudis Avançats (ICREA), Barcelona, Spain
4. Centro de Investigación Biomédica en Red en Cáncer (CIBERONC), Instituto de Salud Carlos III, Madrid, Spain.
5. Department of Medicine and Life Sciences, Universitat Pompeu Fabra, Barcelona, Spain.

Table of contents

Supplementary Figures 3
- Supplementary Figure 1 3
- Supplementary Figure 2 4
- Supplementary Figure 3 11

Supplementary Notes 12
- Supplementary Note 1: Analysis of risk factors of bladder cancer, other tumor types, and clonal hematopoiesis in the UKBiobank cohort 13
- Supplementary Note 2: Sample preparation, duplex protocol and sequencing 22
- Supplementary Note 3: Mutation calling 28
- Supplementary Note 4: Estimation of the error rate of the technology 44
- Supplementary Note 5: Identification of mutational signatures 47
- Supplementary Note 6: Positive selection 54
- Supplementary Note 7: Effect of sequencing depth on the calculation of positive selection 64
- Supplementary Note 8: Analysis of tolerance to errors 68
- Supplementary Note 9: Association of risk factors with the normal urothelium clonal landscape 71
- Supplementary Note 10: Statistical power analysis 81
- Supplementary Note 11: Comparison of normal urothelium and bladder tumors 91
- Supplementary Note 12: Analysis of saturation mutagenesis 96

Supplementary References 99

Supplementary Figures

Supplementary Figure 1

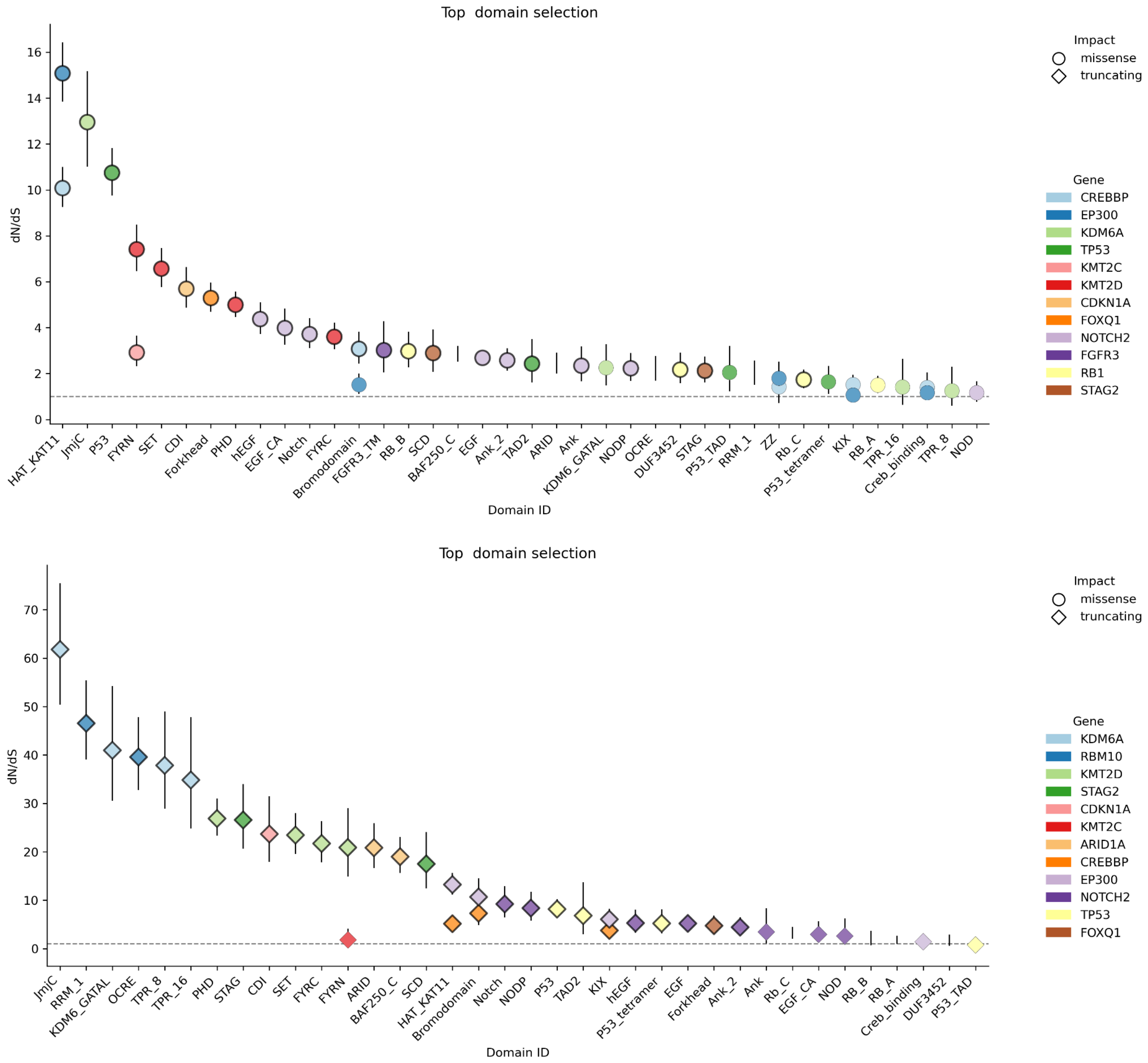

Supplementary Figure 1. Magnitude of positive selection –*dN/dS missense* (top) and *dN/dS truncating* (bottom)– computed for all domains in the genes included in the panel.

Supplementary Figure 2

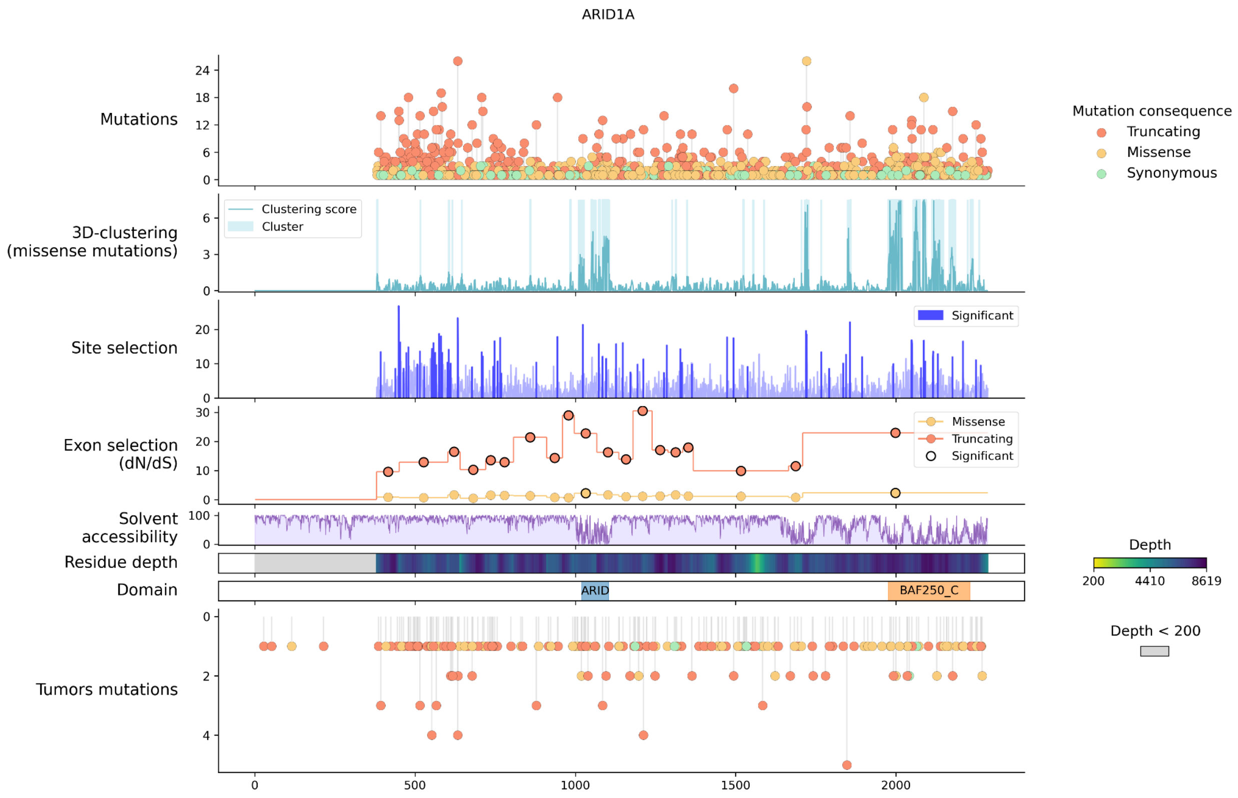

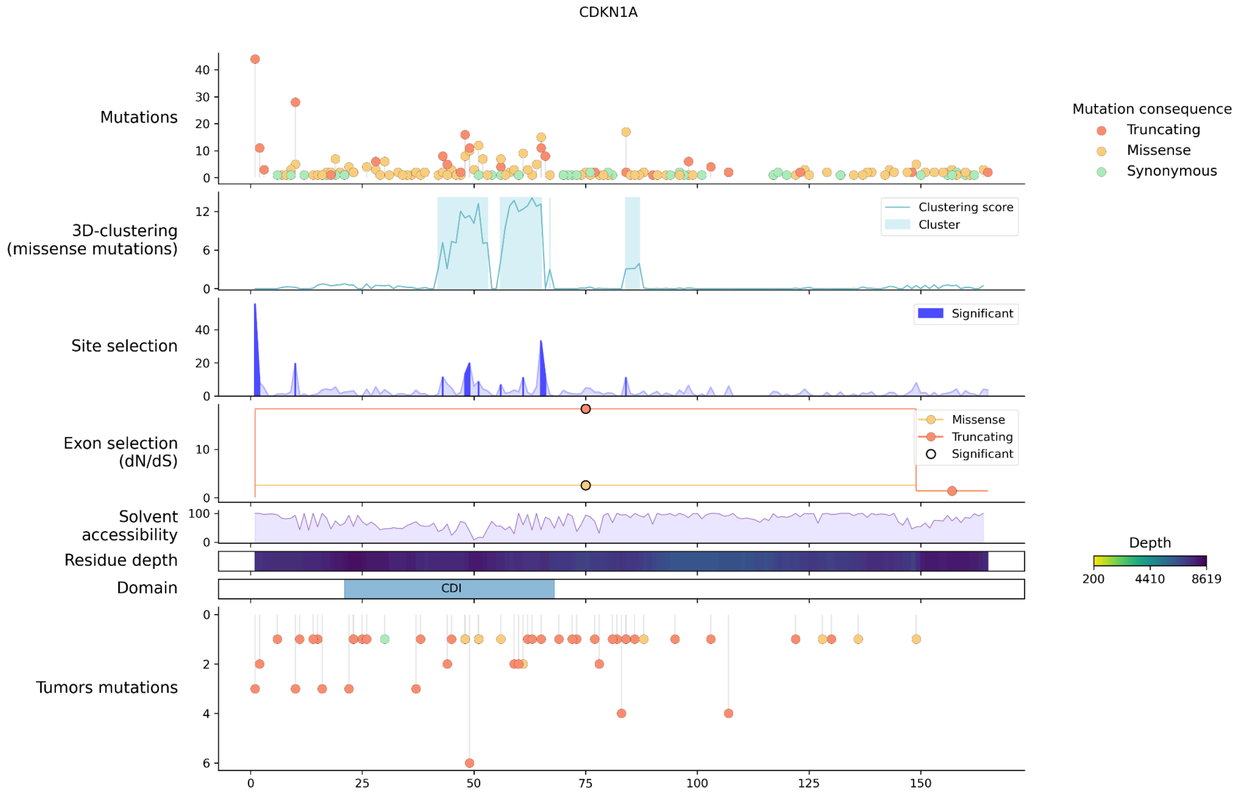

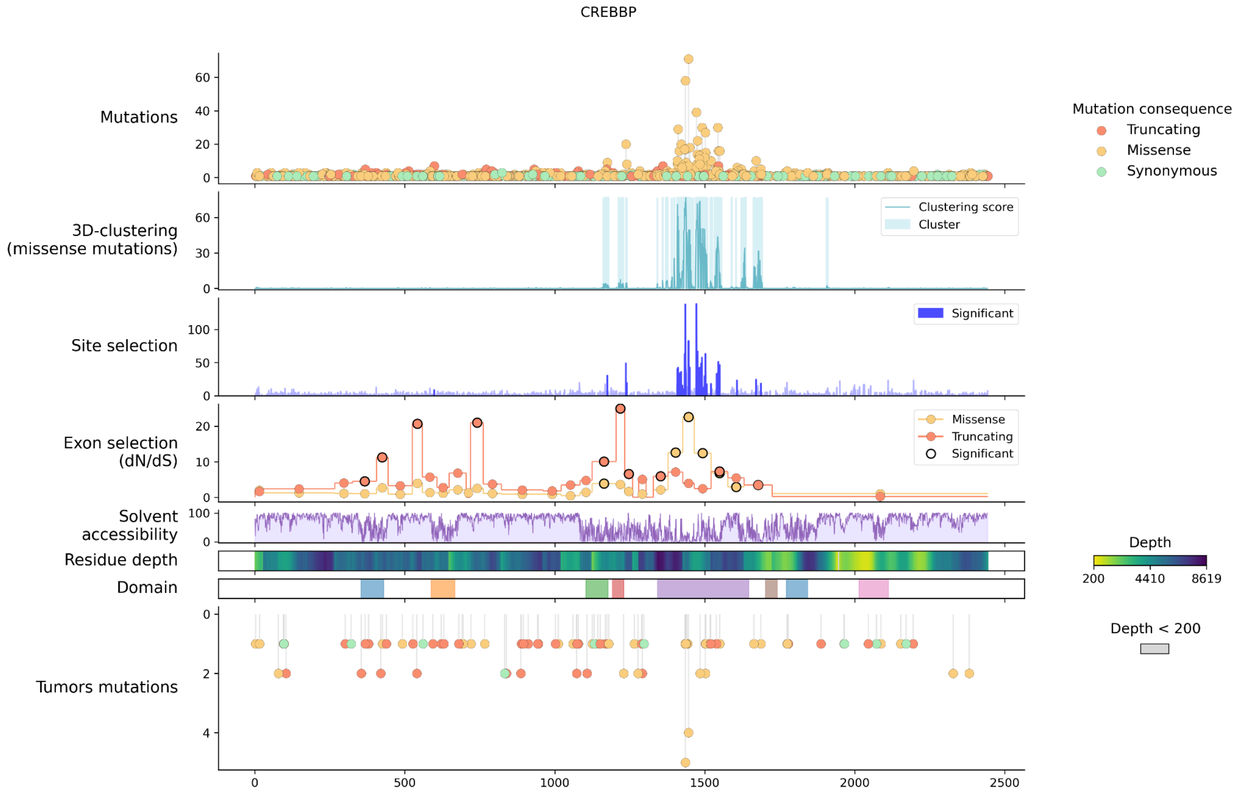
CREBBP
Mutations
3D-clustering (missense mutations)
Site selection
Exon selection (dN/dS)
Solvent accessibility
Residue depth
Domain
Tumors mutations
Mutation consequence
Truncating
Missense
Synonymous
Clustering score
Cluster
Significant
Missense
Truncating
Significant
Depth
200
4410
8619
Depth < 200
0
500
1000
1500
2000
2500

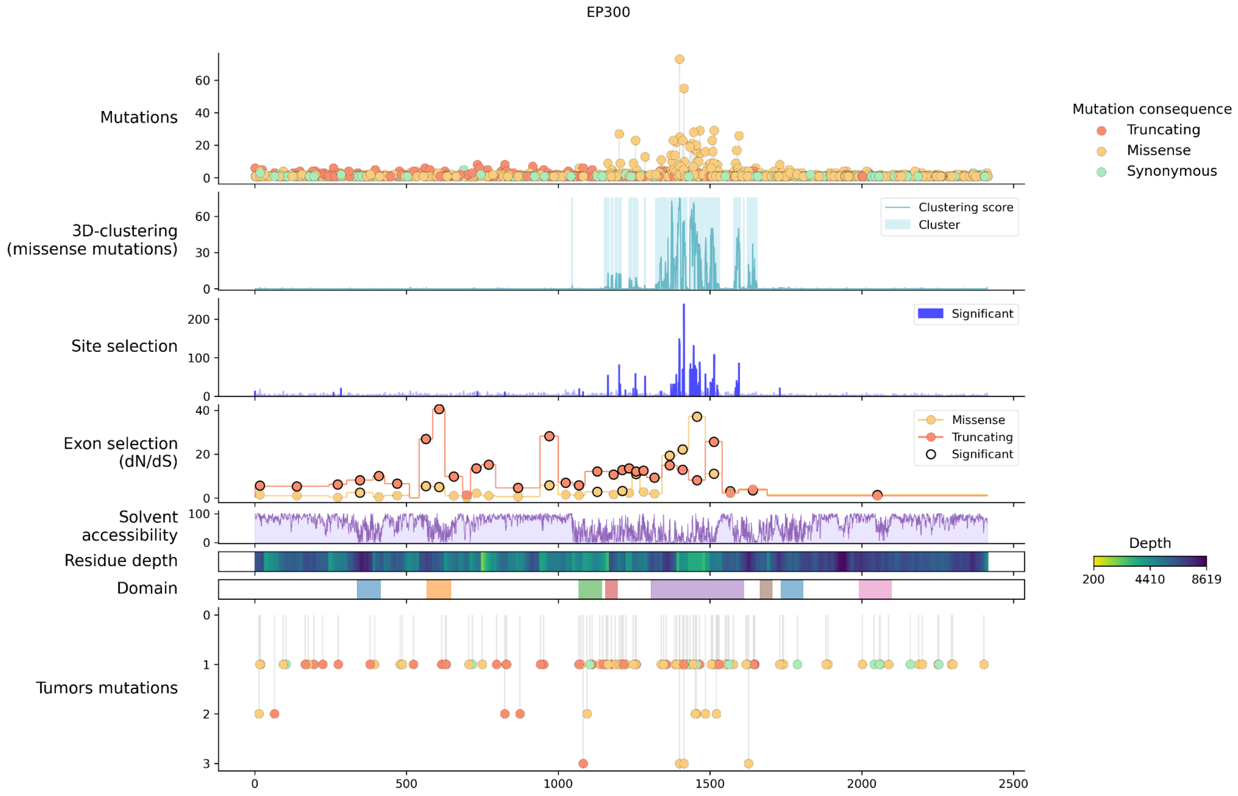
EP300
Mutations
3D-clustering (missense mutations)
Site selection
Exon selection (dN/dS)
Solvent accessibility
Residue depth
Domain
Tumors mutations
Mutation consequence
Truncating
Missense
Synonymous
Clustering score
Cluster
Significant
Missense
Truncating
Significant
Depth
200
4410
8619
0
500
1000
1500
2000
2500

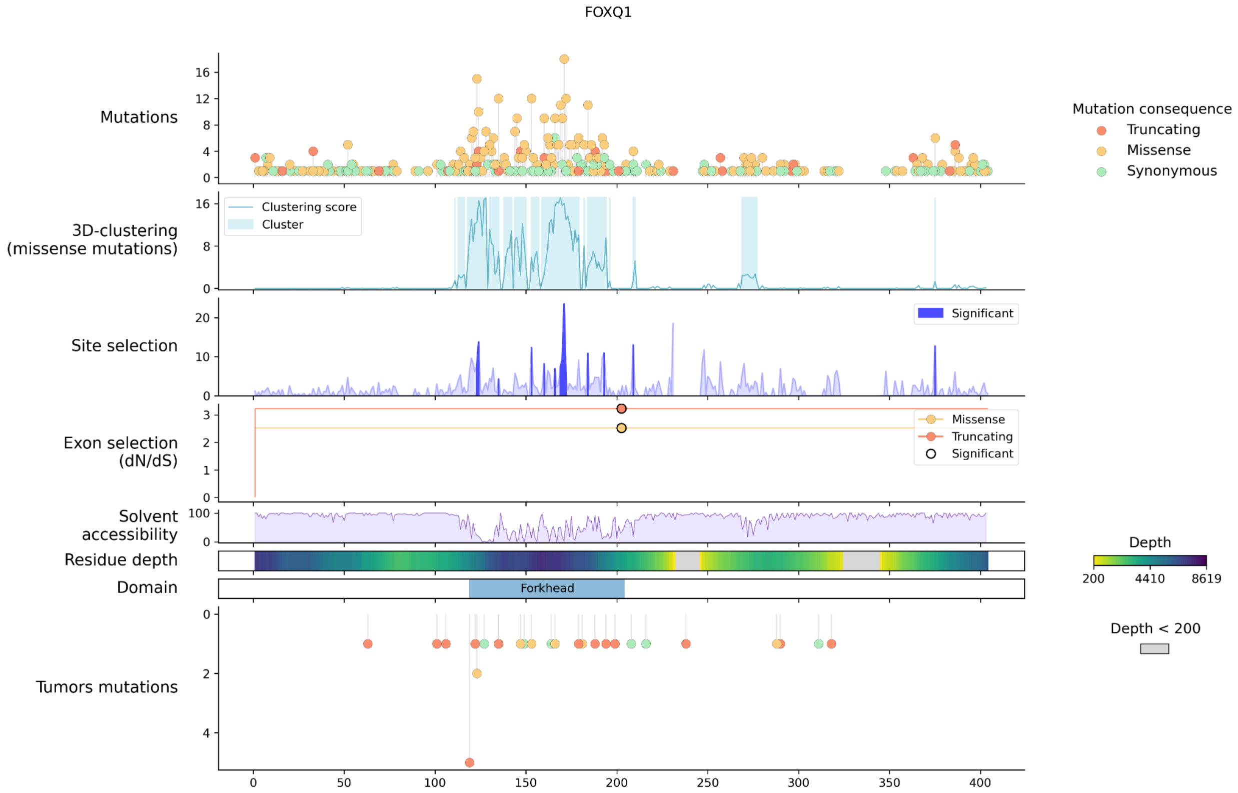
FOXQ1
Mutations
3D-clustering (missense mutations)
Clustering score
Cluster
Site selection
Significant
Exon selection (dN/dS)
Missense
Truncating
Significant
Solvent accessibility
Residue depth
Domain
Forkhead
Tumors mutations
Mutation consequence
Truncating
Missense
Synonymous
Depth
200
4410
8619
Depth < 200

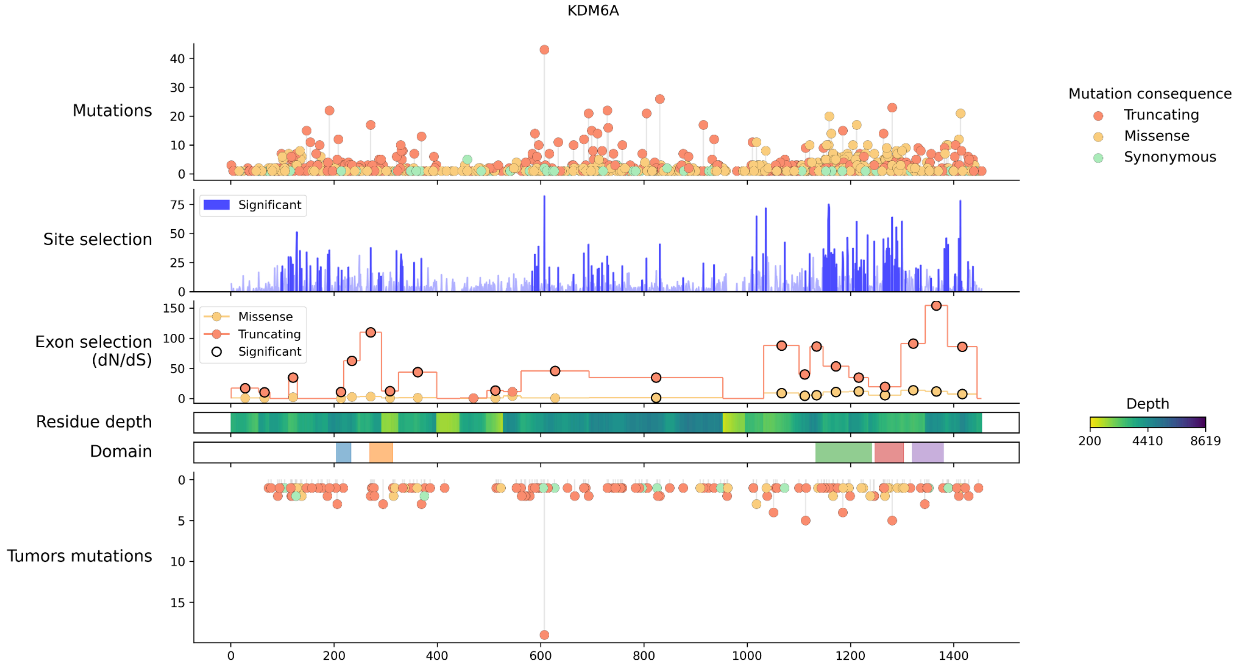
KDM6A
Mutations
Site selection
Significant
Exon selection (dN/dS)
Missense
Truncating
Significant
Residue depth
Domain
Tumors mutations
Mutation consequence
Truncating
Missense
Synonymous
Depth
200
4410
8619

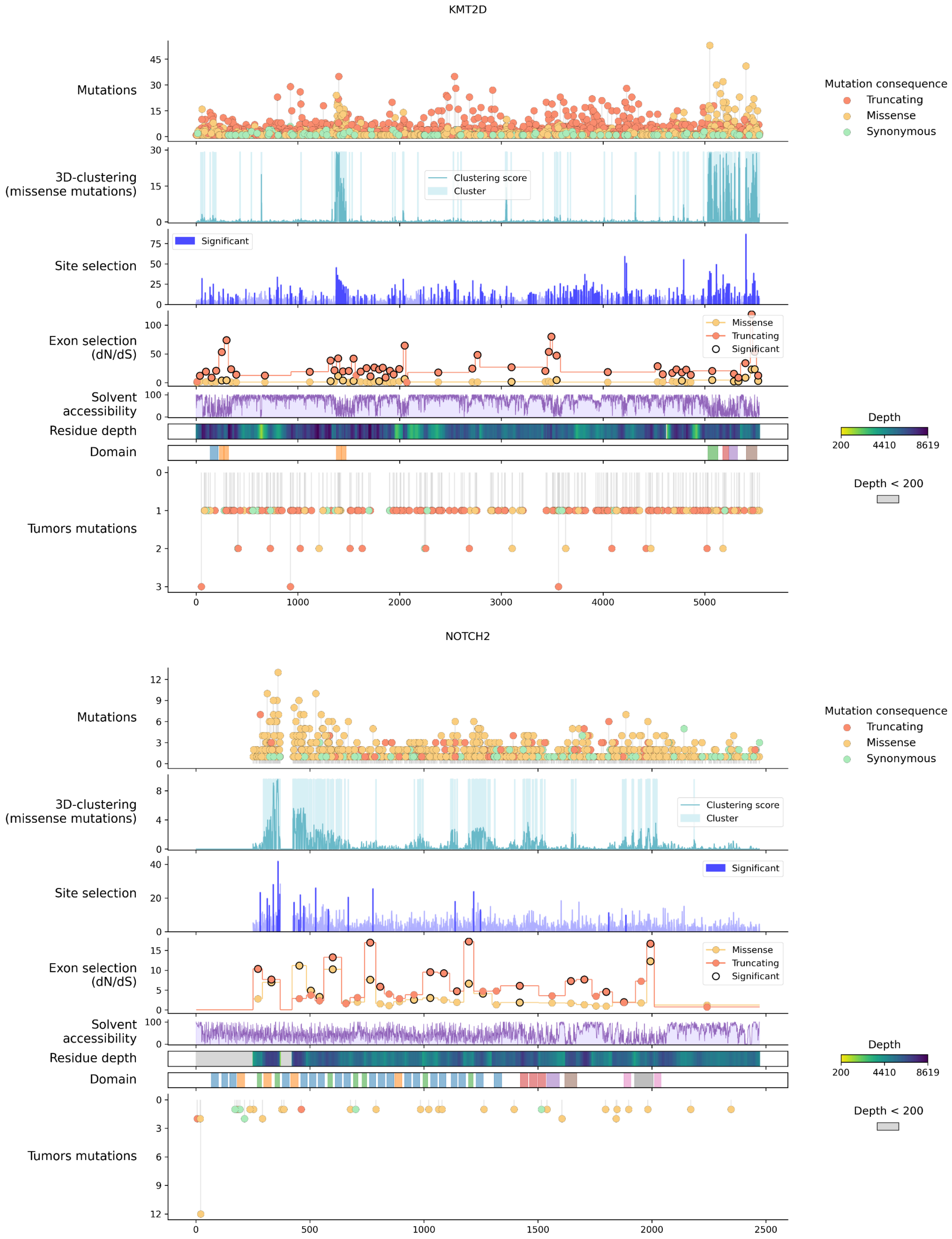
KMT2D
Mutations
3D-clustering (missense mutations)
Site selection
Exon selection (dN/dS)
Solvent accessibility
Residue depth
Domain
Tumors mutations
Mutation consequence
Truncating
Missense
Synonymous
Clustering score
Cluster
Significant
Missense
Truncating
Significant
Depth
200
4410
8619
Depth < 200
0
1000
2000
3000
4000
5000
NOTCH2
Mutations
3D-clustering (missense mutations)
Site selection
Exon selection (dN/dS)
Solvent accessibility
Residue depth
Domain
Tumors mutations
Mutation consequence
Truncating
Missense
Synonymous
Clustering score
Cluster
Significant
Missense
Truncating
Significant
Depth
200
4410
8619
Depth < 200
0
500
1000
1500
2000
2500

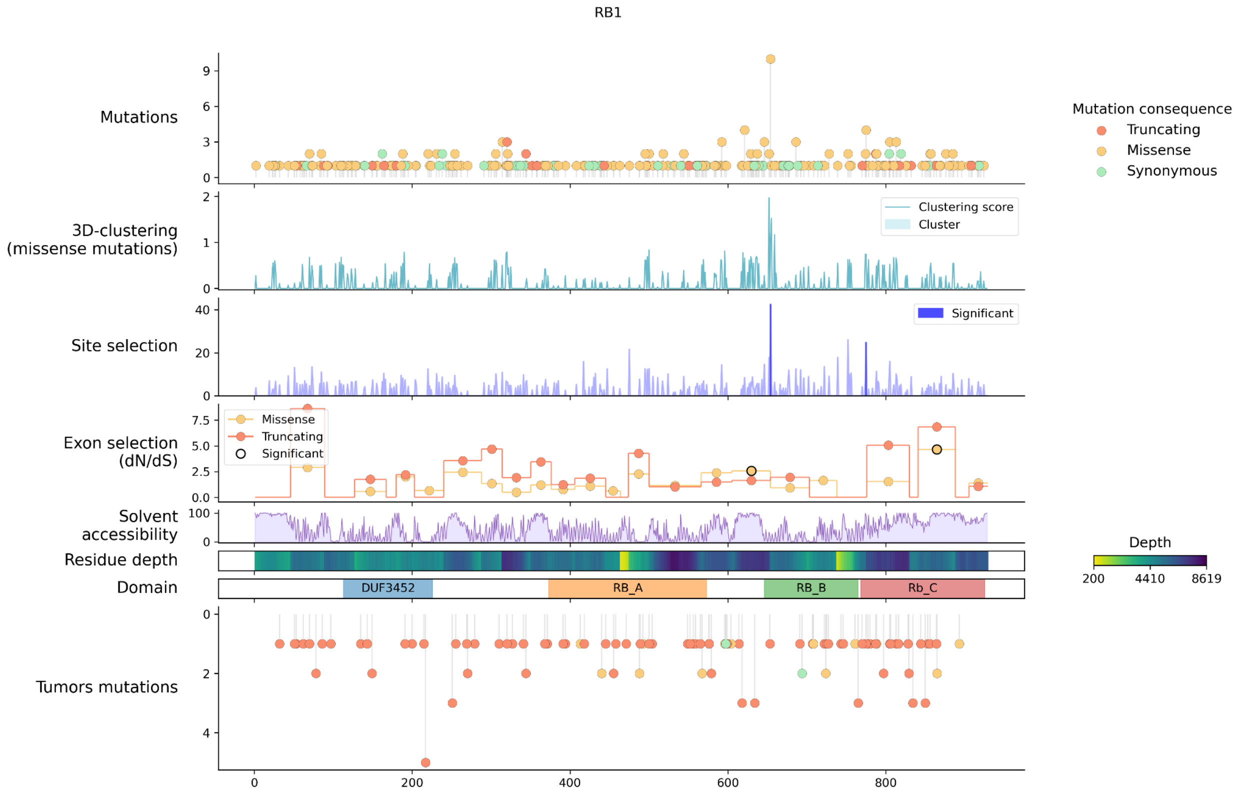
RB1
Mutations
3D-clustering (missense mutations)
Site selection
Exon selection (dN/dS)
Solvent accessibility
Residue depth
Domain
Tumors mutations
Mutation consequence
Truncating
Missense
Synonymous
Clustering score
Cluster
Significant
Missense
Truncating
Significant
Depth
200
4410
8619
DUF3452
RB_A
RB_B
Rb_C
0
200
400
600
800

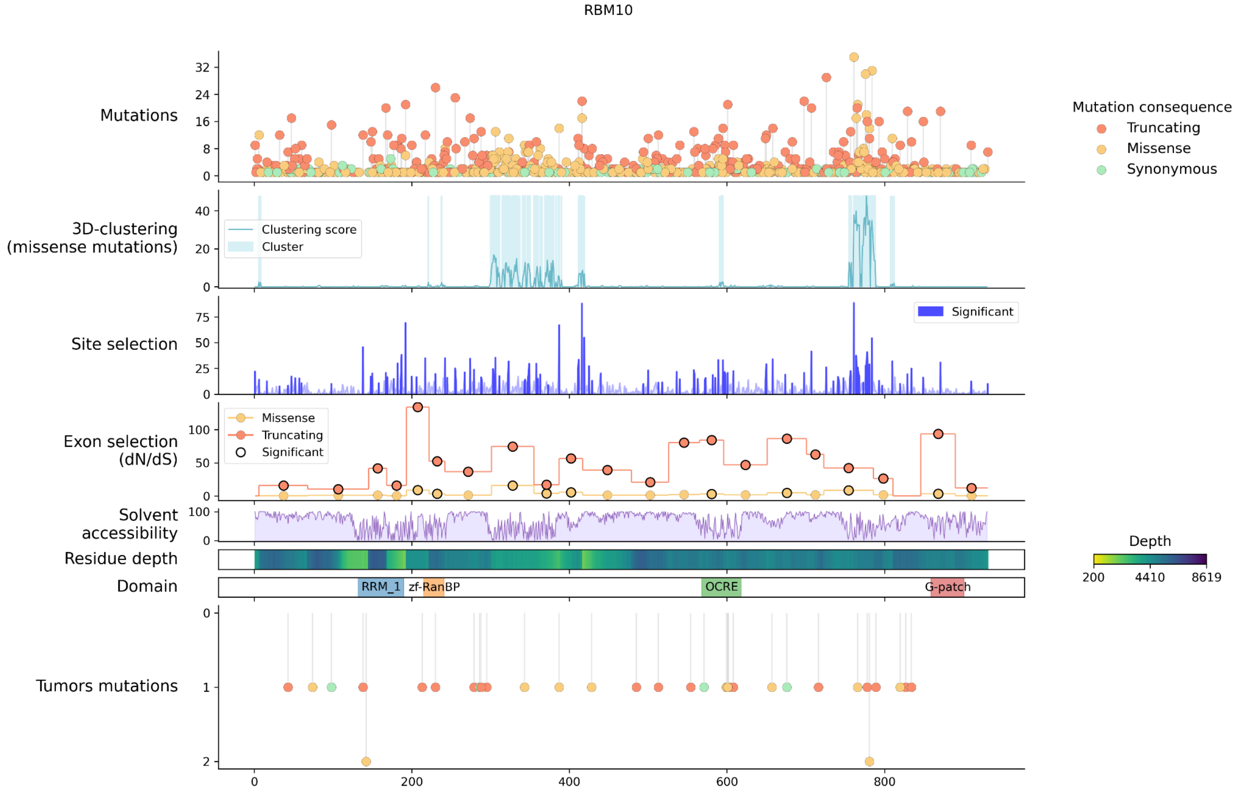
RBM10
Mutations
3D-clustering (missense mutations)
Site selection
Exon selection (dN/dS)
Solvent accessibility
Residue depth
Domain
Tumors mutations
Mutation consequence
Truncating
Missense
Synonymous
Clustering score
Cluster
Significant
Missense
Truncating
Significant
Depth
200
4410
8619
RRM_1
zf-RanBP
OCRE
G-patch
0
200
400
600
800

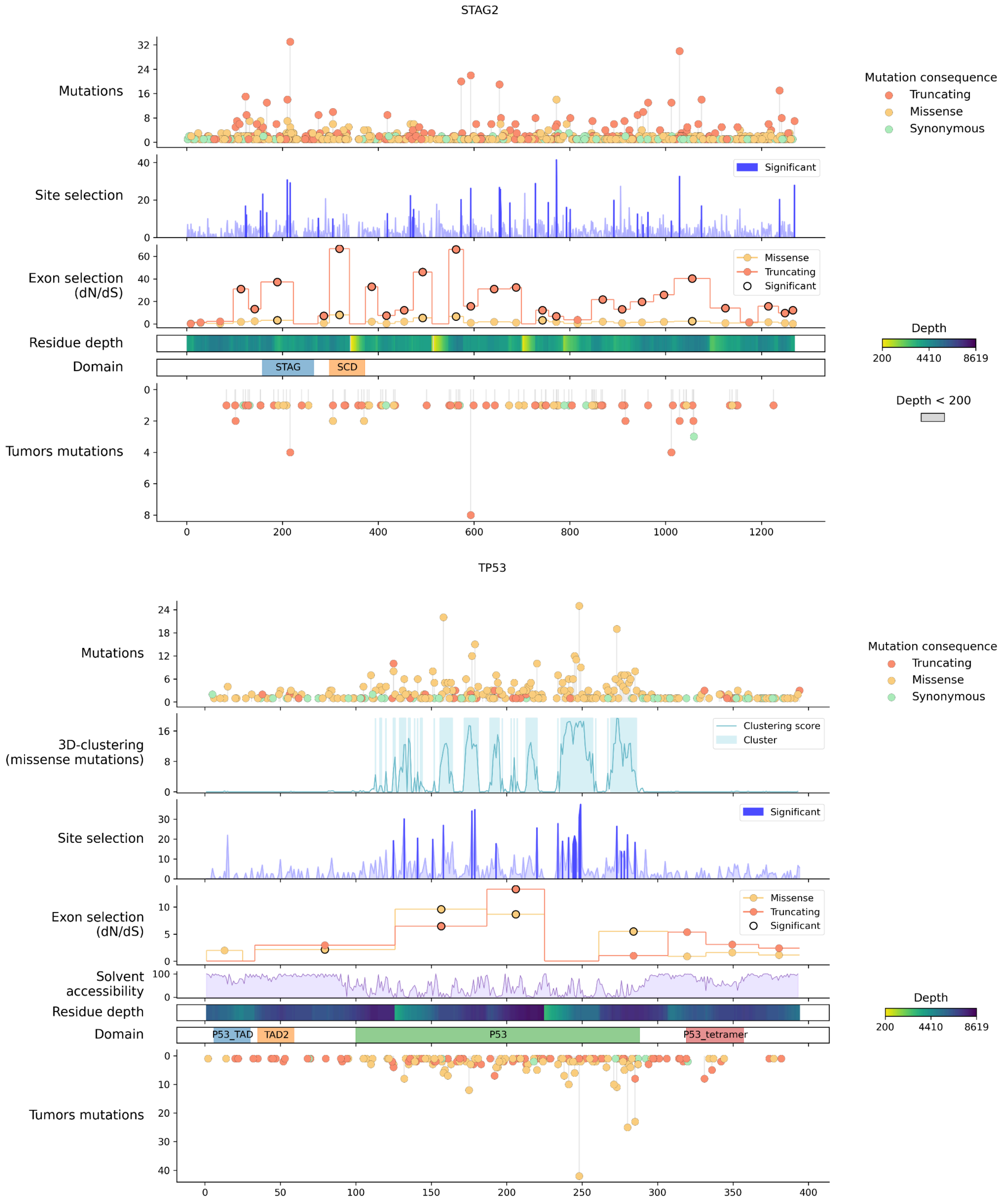

Supplementary Figure 2. Natural saturation mutagenesis plots (analogous to those in Extended Data Fig. 9) for 12 genes. From top to bottom, tracks include: distribution of truncating, missense and synonymous mutations along the coding sequence of the gene (first); 3D clusters obtained via Oncodrive3D (second), site selection computed for each amino acid residue (third); *dN/dS truncating* and *dN/dS missense* values for each exon (fourth); solvent accessibility along the protein sequence (fifth);

duplex sequencing depth per amino acid residue (sixth); protein domains (seventh); and the distribution of tumor mutations (from intOGen; see Methods) along the sequence of the gene (last).

Supplementary Figure 3

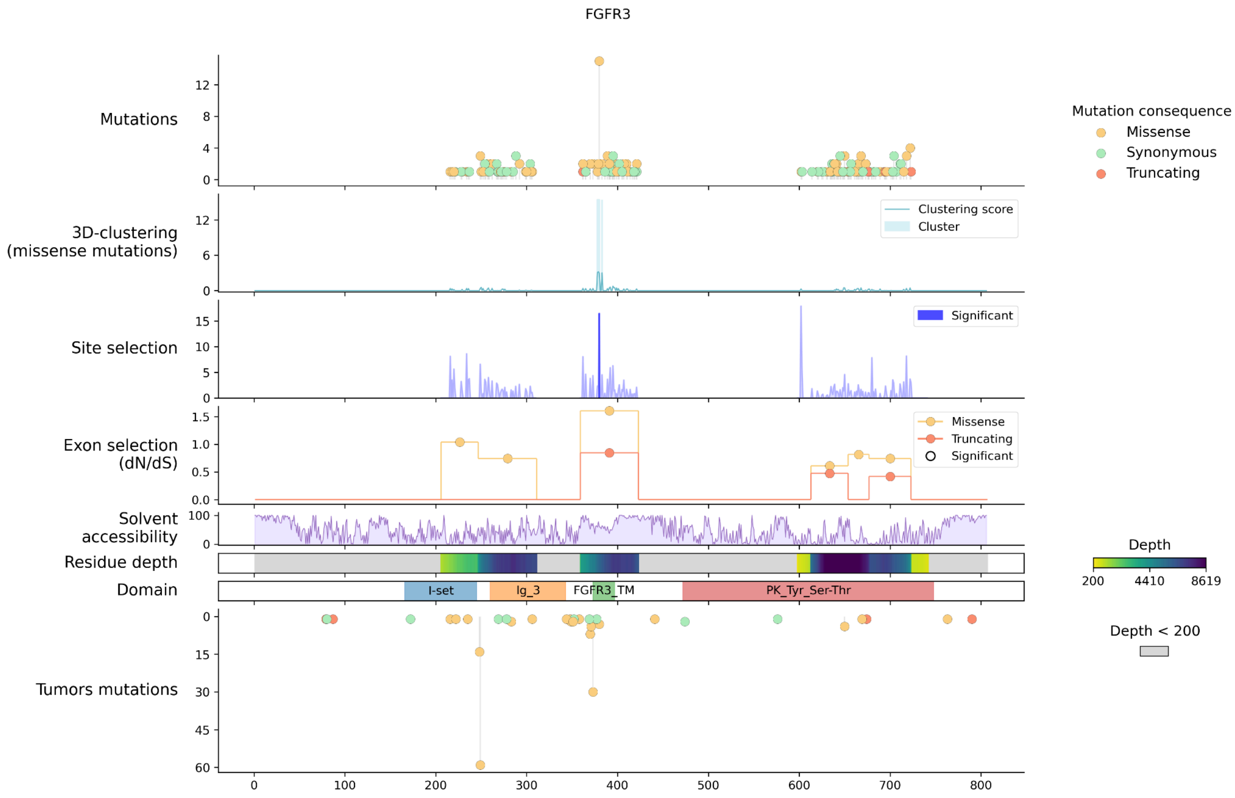

Supplementary Figure 3. Natural saturation mutagenesis plots (analogous to those in Extended Data Fig. 9) for FGFR3. Only specific regions with hotspots in tumors were sequenced. The hotspot seen in normal urothelium (G380) does not correspond to the three most common hotspots in bladder tumors (R248, S249, Y373).

Supplementary Notes

Supplementary Note 1: Analysis of risk factors of bladder cancer, other tumor types, and clonal hematopoiesis in the UKBiobank cohort

Abstract
Several global studies, such as those led by the International Agency for Research on Cancer have established a link between sex and smoking history and the incidence of bladder cancer. In this Supplementary Note we explore this link across the UK Biobank cohort, comprising ~500,000 individuals. Specifically, we ask whether sex and smoking are associated with the observed age-corrected risk of bladder cancer in this cohort independently of each other and of other bladder cancer risk factors. The results of this analysis show that sex is the factor with the largest contribution to bladder cancer risk, independently of smoking and others. A history of smoking is also significantly associated with the risk of this type of cancer. We use this verification as the starting point of the analyses described in the main manuscript, which aim to determine whether sex and smoking also influence the clonal landscape of the normal bladder urothelium.

Population features associated with bladder cancer risk
We tested the association of several population features with the risk of bladder cancer across the ~500,000 individuals in the UK Biobank (UKB[1]) (see details in the Methods section of this Note). Upon a thorough search of the literature summarizing epidemiological studies[2–4], we selected well established risk factors, namely sex (as bladder cancer incidence within males is known to be ~4 times higher than within females), tobacco smoking, occupational exposure to paints, chemical and diesel exhaust, exposure to environmental carcinogens such as arsenic, opium consumption, history of pelvic radiotherapy, history of cyclophosphamide or chlornaphazine medication, infection by schistosomiasis, X radiation, and gamma radiation. We also included factors with less evidence of association with bladder cancer risk: E-cigarette usage, alcohol intake, level of physical activity, history of urinary tract infections, Body Mass Index (BMI), metabolic syndrome, hypertension, diabetes, stroke, asthma, lupus, vasculitis, gout, and history of pioglitazone medication. While we had analyzed a few of these factors (i.e., sex, tobacco smoking, and chemo/radiotherapy) for their association with the clonal structure of normal urothelium, the information of the majority of these factors was not available for the 45 donors in the DNA duplex sequencing-probed cohort.

After extracting and processing the clinical data at UKB as described in the Methods section of this Note, we tested the association of sex, smoking history, occupational exposure (self-reported) to paints, diesel exhaust or chemicals in general, obesity (BMI>=30), history of hypertension, history of diabetes mellitus type 2, history of urinary tract infections, history of stroke, alcohol intake, and use of pioglitazone with bladder cancer risk (Supplementary Table 8). First, we carried out a univariate Cox regression for each of these factors only correcting for ancestry (Supplementary Figure 4).

Supplementary Table 8. Bladder cancer risk factors in the UKB.

Variable	Code		Num. of cases	Num. of bladder cancer cases	Bladder cancer Frequency (%)
Sex	0	Female	254,602	719	0.282
	1	Male	215,140	2,040	0.948
Smoking history	0	Never smokers	255,782	897	0.351
	1	Previous/current smokers	211,567	1,849	0.874
Occupational exposures	0	Non exposed	101,251	440	0.435
	1	Exposed	12,017	89	0.741
Obesity (BMI>=30)	0	No	353,865	1,917	0.542
	1	Yes	114,220	832	0.728
History of hypertension	0	No	285,718	1,031	0.361
	1	Yes	184,024	1,728	0.939
History of Diabetes Mellitus Type 2 (DMT2)	0	No	424,641	2,235	0.526
	1	Yes	45,101	524	1.162
History of urinary tract infections	0	No	443,454	2,654	0.598
	1	Yes	26,288	105	0.399
History of stroke	0	No	452,829	2,687	0.593
	1	Yes	16,913	72	0.426
Alcohol intake	0	Never/occasionally	372,091	950	0.255
	1	Daily or almost daily	95,182	375	0.394
Pioglitazone history	0	No	467,234	1,324	0.283
	1	Yes	1,076	3	0.279

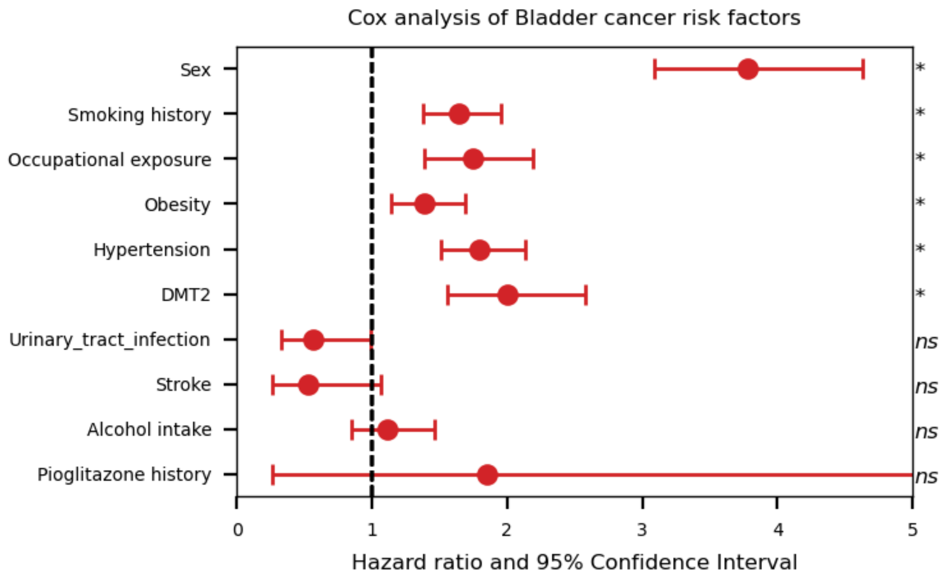

Supplementary Figure 4. Association of variables with the risk of developing bladder cancer measured through univariate Cox regression analyses taking into account ancestry.
Each variable was analyzed separately in a univariate test, correcting for ancestry. Asterisks represent significant associations (FDR<0.05); ns: non-significant. DMT2, Diabetes Mellitus Type 2.

This analysis showed that sex (being male), history of tobacco smoking, frequent occupational exposures to paint, diesel exhaust or chemicals in general, obesity (BMI>=30), and history of hypertension or diabetes mellitus type 2 are significantly associated with an increase of the risk of bladder cancer across UKB. However, this univariate analysis does not account for confounding (or any type of interaction) between these features, and therefore, it does not yield the independent magnitude of influence of each factor on the risk of bladder cancer. Hence, we next explored the correlation between these features across the cohort (Supplementary Figure 5).

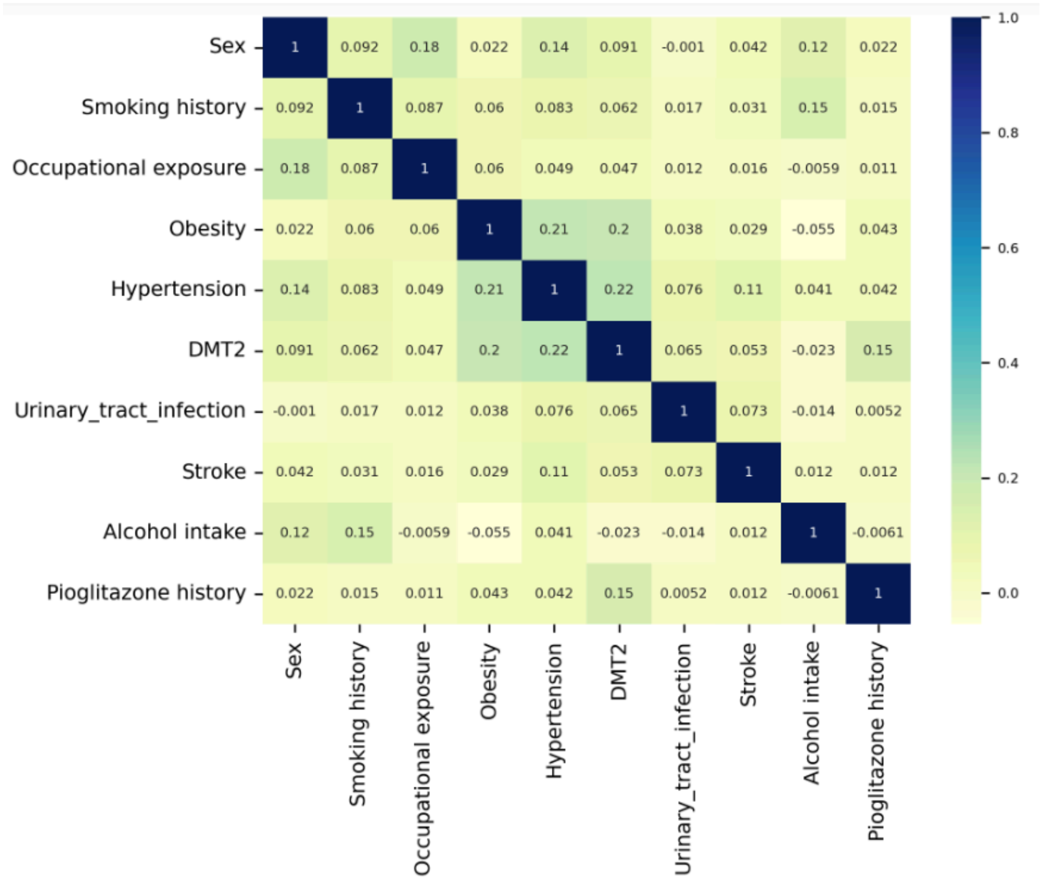

Supplementary Figure 5. Correlogram of features included in the univariate analysis.
Each cell in the heatmap presents the Pearson's correlation coefficient between a pair of features. DMT2, Diabetes Mellitus Type 2.

We found a relatively high (expected) degree of correlation between obesity, history of hypertension, and diabetes mellitus type 2. Thus, in the multivariate analysis, we decided to include only obesity to reduce the number of covariates. We used a multivariate Cox regression model to test the combined association with bladder cancer of the four variables that were significant in univariate analyses: sex, history of tobacco smoking, history of occupational exposures to paints, diesel exhaust or any chemicals, and obesity, correcting for ancestry. (Supplementary Figure 6 and Supplementary Table 9).

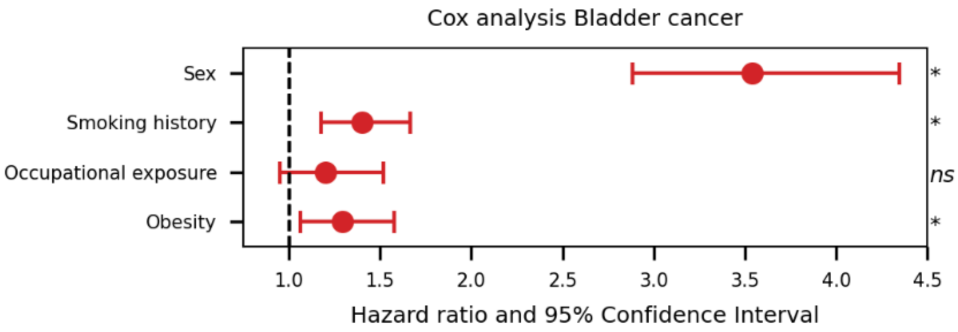

Supplementary Figure 6. Result of multivariate Cox regression analysis of bladder cancer risk. (Ancestry, included as covariate, is not shown.) Asterisks represent significant associations (FDR<0.05); ns: non-significant.

Supplementary Table 9. Result of multivariate Cox regression analysis of bladder cancer risk.

variable	hazard_ratio	confint_1st	confint_2nd	pval	q_value	sig
Sex	3.540859	2.884135	4.347119	1.348992e-33	5.395969e-33	sig
Smoking history	1.399610	1.175344	1.666668	1.611582e-04	3.223164e-04	sig
Occupational exposure	1.200789	0.950660	1.516730	1.246892e-01	1.246892e-01	ns
Obesity	1.295296	1.062992	1.578369	1.029620e-02	1.372827e-02	sig

In the multivariate analysis, sex (being male), a history of smoking, and obesity still appear significantly associated with an increased risk of bladder cancer. However, having a history of frequent occupational exposure to paint, diesel exhaust or any chemical, while still showing a positive effect, is not significant. This may be because occupational exposure is confounded with sex, or due to the relatively smaller number of donors in the cohort with annotations pertaining to this variable.

To confirm that sex (being male) is associated with an increase in bladder cancer risk independently of smoking history, we carried out Cox univariate analyses testing this association exclusively across current smokers, former smokers and never smokers. Sex appears significantly associated with the risk of bladder cancer across the three groups of UKB donors (Supplementary Fig. 7), confirming that its effect is independent from smoking.

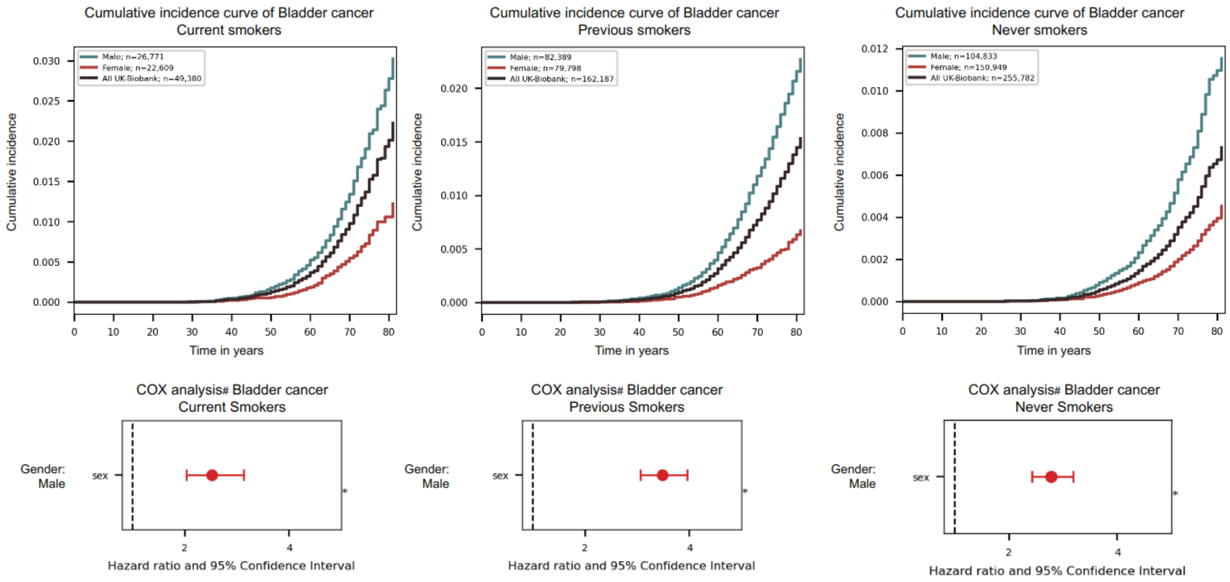

Supplementary Figure 7. Association of sex with the risk of developing bladder cancer across current smokers, former smokers and never smokers in the UKB.
The three graphs in the top row present the Kaplan-Meier incidence curves for (from left to right) current smokers, former smokers, and never smokers. The incidence curves for males and females are shown separately and compared to that of all UKB donors. The three graphs in the bottom row present univariate Cox regression analyses measuring the increased hazard ratios of male (from left to right) current smokers, former smokers, and never smokers compared to females in the same three groups.

Sex bias in the incidence of different tumor types across UKB

We asked whether differences in the incidence per sex can be observed in different cancer types in the general population. To this end, we used data of cancer incidence for ~500,000 individuals in the UK Biobank (UKB) (see details in the Methods section of this Supplementary Note).

Supplementary Figure 8 shows the fraction of men and women affected by different types of cancer at some point of their life after their recruitment in UKB. We included tumor types analyzed in a previous study[5], with at least 500 cases observed in UKB.

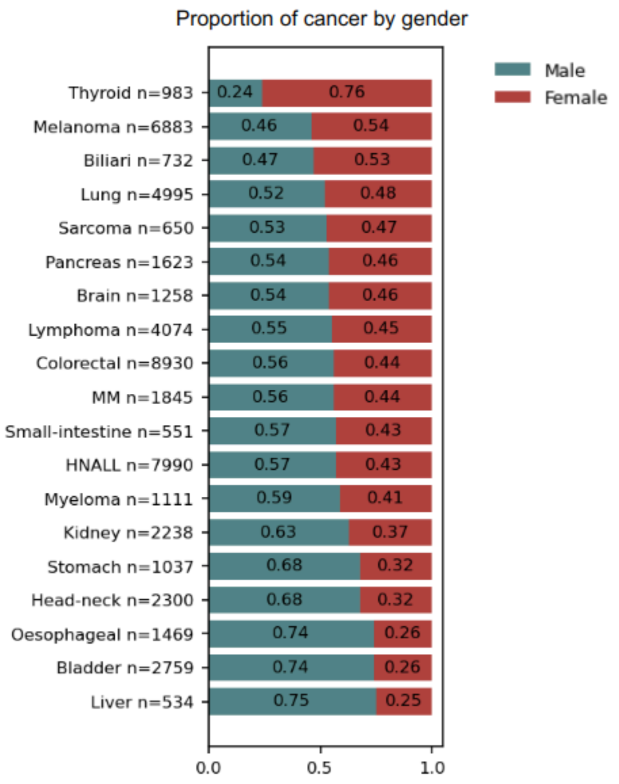

Supplementary Figure 8. Fraction of males and females affected by different types of cancer in UKB.
The total number of cases of each cancer type in UKB is shown to the left of the bar plots. MM, Myeloid malignancies. HNALL, All hematological neoplasms.

To test for sex bias in the incidence of these different types of cancer, we carried out multivariate Cox regressions including smoking, BMI, and ancestry as covariates (Supplementary Fig. 9, left panel). We observed significant differences in incidence for both sexes across several cancer types. The most pronounced differences corresponded to bladder cancer (OR 3.04 [2.79-3.3 CI]), esophageal tumors (OR 2.97 [2.64-3.34 CI]) and liver cancer (3.27 [2.69-3.99 CI]). These differences were maintained when the analysis was restricted to donors with no history of malignancy prior to the cancer type analyzed (Supplementary Fig. 9, right panel). These results are concordant with a prior study from the National Institutes of Health-AARP Diet and Health Study, which reported higher incidence of cancer in males than females for almost all anatomical sites, which was largely unexplained by other risk factors[6].

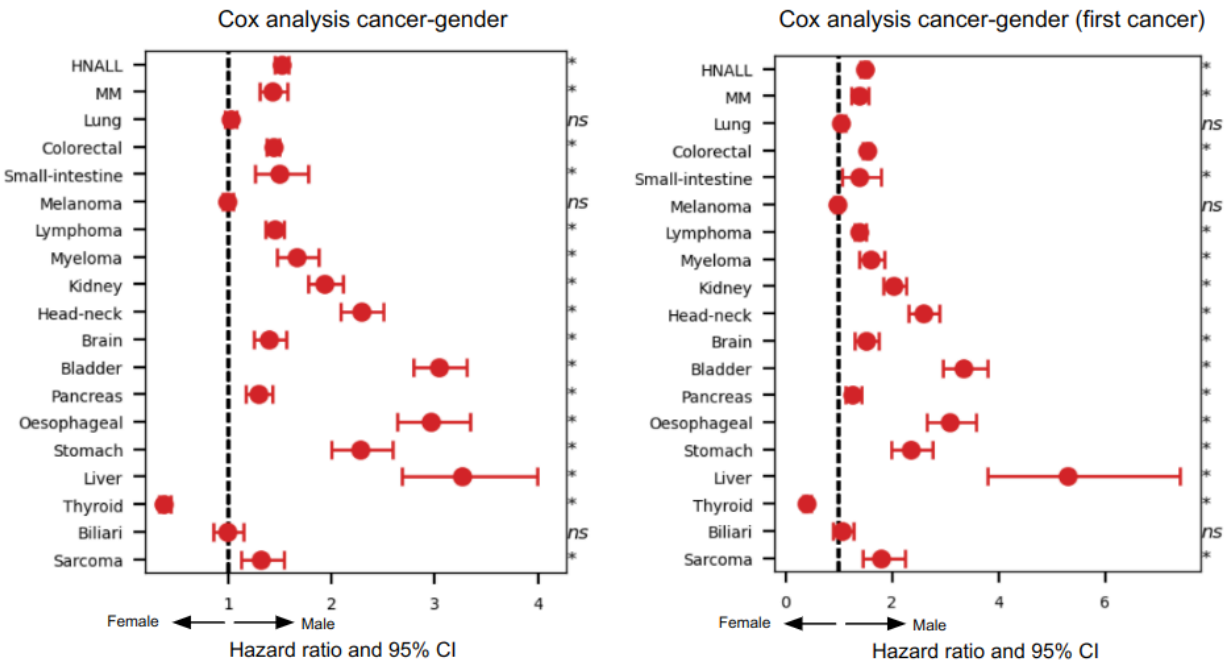

Supplementary Figure 9. Multivariate Cox regression testing the association of the sex of donors with the incidence of different cancer types across UKB.
Left: all cases with malignancies reported after UKB recruitment; Right: only cases with no history of malignancy prior to their cancer type analyzed. HNALL, All hematologic neoplasms; MM,Myeloid Malignancies. CI, confidence interval. ns: non-significant.

Methods

UKB access

This research has been conducted using the UK Biobank Resource under application number 69794. All analyses were performed with the UK Biobank Research Analysis Platform.

Data preparation

The cohort used in the study comprises 469,742 individuals (54% females). Since the last clinical reports were from 2021 the current age was recalculated with age range 40–87 and median age 69 years old. Clinical data were downloaded in November 2022 and individual traits were pulled out from the whole phenotype file classified in data-fields. Basic information from the individuals used for the analyses was year of birth (data-field: 34), sex (data-field: 31), genetic principal components (data-field: 22009), age at death (data-field: 40007), and body mass index (BMI, data-field: 21001).

Cancer diagnosis was annotated combining information from different data-fields including ICD-10 cancer diagnosis (data-field: 40006), ICD-9 cancer diagnosis (data-field: 40013), self-reported cancer (data-field: 20001), and underlying cause of death (data-field: 40001), as in ref [5]. Other diseases such as stroke or urinary tract infection were annotated combining

information from different data-fields including ICD-10 diagnosis (data-fields: 41202, 41270), ICD-9 diagnosis (data-fields: 41203, 41271), self-reported non-cancer illness (data-field: 20002), and underlying cause of death (data-field: 40001), as in refs.[5,7]. For each definition, the first diagnosis event that occurred was selected. Age at first occurrence was calculated using the difference between the year of birth and specific diagnosis dates (data-fields: 40005, 40000, 41260, 41262, 41263, 41280, 41281, 41282) or directly from the diagnosis age (data-fields: 20007, 20009).

Occupational exposure was annotated from different data-fields: chemicals or other fumes (data-field: 22610), cigarette smoke from other people smoking (data-field:22611), materials containing asbestos (data-field: 22612), paints, thinners or glues (data-field: 22613), pesticides (data-field: 22614), and diesel exhaust (data-field: 22615). Job code was obtained from data-field 22601, and duration of the job was obtained from the year starting job (data-field: 22602) and the year of finishing the job (data-field: 22603).

Smoking status was defined as never smoker or ever smoker using smoking status information (data-field: 20116). Alcohol intake frequency was defined as daily/almost daily drinker or occasionally drinker using data-field 1558. Since information was obtained from the initial assessment visit (2006-2010), we removed those cases in which the bladder cancer occurred before 2010. Use of pioglitazone was obtained from medication records (data-field: 20003), removing those cases in which the bladder cancer occurred before 2010.

Regarding some other covariates: diabetes mellitus type II was defined as its diagnosis or treatment with insulin or oral hypoglycemic medication (data-field: 6177); hypertension was defined by its diagnosis or by having a systolic blood pressure ≥140 mmHg (data-field: 4080), diastolic blood pressure ≥90 mmHg (data-field: 4079), or use of antihypertensive medication (data-field: 6177).

Cox regressions and Kaplan-Meier incidence curves

To analyze the risk of different types of cancer, we performed Cox regression with CoxPHFitter (Python lifelines package v.0.27.8). We counted as an event any reported diagnosis of cancer. The value of years to cancer events was calculated from the year of birth. Individuals without the event who died before the end of the follow-up were censored at the time of death, while the rest were censored at the last follow-up reported (2021-06-25, from data-field 40005). The maximum number of years to an event was restricted to the 97th percentile of the UKB population. Kaplan-Meier curves were built using KaplanMeierFitter and logrank_test functions (Python lifelines package v.0.27.8). Regressions were adjusted by smoking history, ancestry, and BMI when required.

Supplementary Note 2: Sample preparation, duplex protocol and sequencing

Abstract

This Supplementary Note describes the collection of normal urothelium of the individuals in the cohort, sample processing, and preparation of the DNA duplex sequencing libraries. It provides a succinct description of the cohort and the clinical data obtained from the donors, which is presented at length in Supplementary Table 1. Moreover, it describes the design of the panel of 16 genomic regions employed in the study. The Note also explains the controls carried out to verify that the cells collected through brushing are epithelial in their vast majority.

Patients and Sample Collection

To analyze the clonal landscape of normal bladder we reasoned that wide brushing of the epithelial surface would provide maximum enrichment of urothelial cells which, coupled with ultradeep DNA duplex sequencing (Supplementary Figure 10, top panel) would enable high resolution detection of clonal expansions, if present. This approach contrasts with the limited resolution of standard next generation sequencing (Supplementary Figure 10, bottom panel) which is sufficient to detect the mutations driving the clonal expansion of tumors but insufficient to detect mutations in polyclonal normal tissue samples where clones are present at very low frequency.

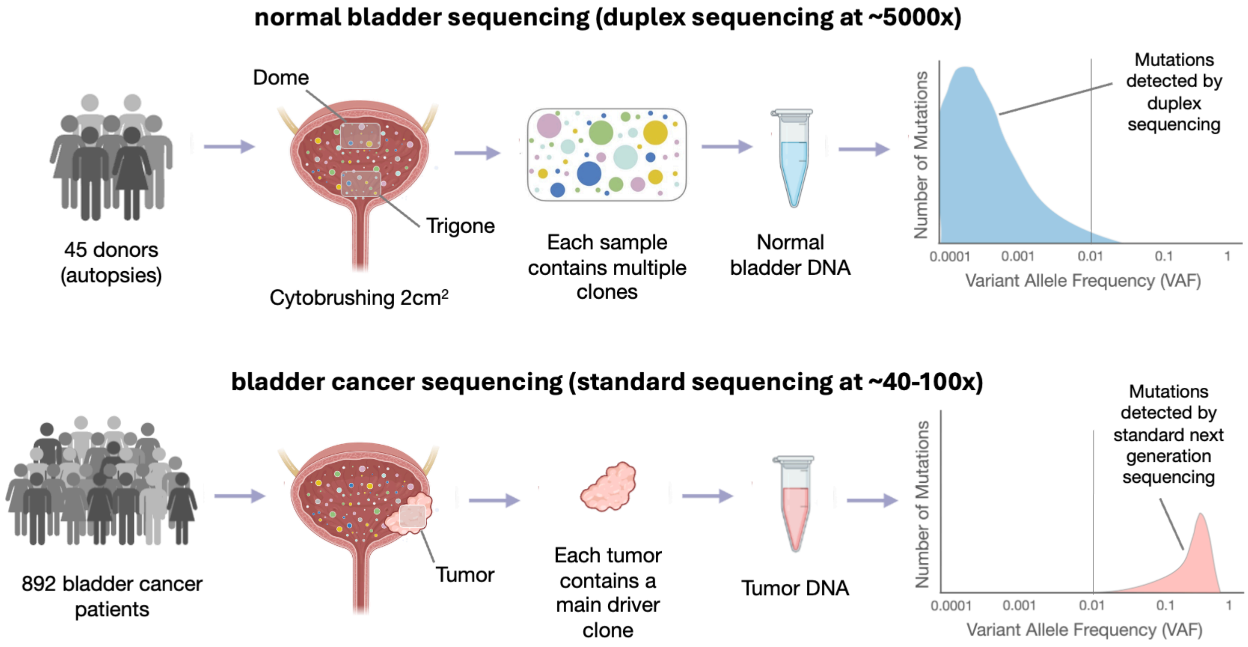

Supplementary Figure 10. Schematic representation of study design and advantage of ultradeep DNA duplex sequencing for sensitive detection of clonal expansions in normal tissue.
Top panel, cytobrushing of dome and trigone in normal bladders at autopsy enables the sampling of multitude of clones present in the urothelium. These clones are composed of small numbers of cells that share a driver mutation. Thus, driver mutations are present at very low Variant Allele Frequency (VAF) in the sample DNA but they can be detected by sequencing at very high depth (mean 5,000x) with duplex

DNA sequencing. Bottom panel, current mutational data for bladder cancers derives from hundreds of patients whose tumors were sequenced in prior studies (intOGen[8] includes 892 bladder cancers). Tumors typically contain only 1 or a few clonal driver mutations, which are readily detected by standard next generation sequencing because of their high VAF. Vertical grey lines indicate the limit of detection of standard next generation sequencing.

We performed urothelial cytobrushing in 53 individuals that underwent autopsy at the University of Washington. Individuals were included if they had no history or evidence (upon autopsy) of gross bladder pathology or bladder cancer. Review of the autopsy report and hematoxylin and eosin (H&E) stained slides revealed active or chronic inflammation with hemorrhages and mucosal erosions in 3 individuals, who were excluded from the study. The average postmortem interval was 4 days (min 1, max 9). Normal urothelium was collected at time of autopsy using an epithelial brush covering a region of 2-3 cm^2. The brush was immediately placed in a 15ml tube containing 10ml of CytoLyt (Hologic) fixative and stored at 4°C until DNA extraction. Two epithelial brushes were collected for each individual, one from the bladder top (dome) and one from the bladder floor (trigone). Most individuals (34) had successful duplex sequencing of both samples. 11 individuals only had data for dome or trigone because of insufficient or too fragmented DNA (DIN <1.4) in the paired sample. Four individuals had insufficient DNA in both samples and were excluded. One individual had successful sequencing in both samples but the mutational profile indicated potential artifacts and thus was also excluded. Thus, the final number of individuals with available data for at least one sample was 45 (Supplementary Table 1) and the final number of samples processed was 79 (Supplementary Table 4).

The following clinical information was obtained from the medical record for each donor: age, sex, BMI (at the time of death), tobacco smoking history, alcohol intake, race/ethnicity, history of urinary tract infections, cancer history, and chemotherapy exposure (Supplementary Table 1). Because race/ethnicity categories other than white had 2 or less individuals, this variable was not analyzed. Smoking was categorized as never, former, or current. For statistical analyses, smoking was dichotomized as no (never) or yes (current or former). Alcohol use was categorized as heavy, moderate, occasional, former, or never based on NIAA guidelines (Drinking Levels and Patterns Defined). For men, heavy drinking was defined as consuming 5 or more drinks on any day or 15 or more per week, and moderate drinking was defined as 2 drinks or less in a day. For women, heavy drinking was defined as consuming 4 or more on any day, or 8 or more drinks per week, and moderate drinking was defined as 1 drink or less in a day. For statistical analyses alcohol drinking history was categorized as never vs ever (heavy, moderate, occasional, and former). Chemotherapy history was extracted from the medical record and categorized as yes or no (yes including any type of chemotherapy or radiotherapy).

Cellular composition of urothelial brushes

The cellular composition of urothelial brushes was investigated by immunohistochemistry (IHC) (Supplementary Figure 11). Three urothelial brushes from two patients (two domes and one trigone) were collected in Cytolyt, spun down, formalin fixed, and paraffin embedded (FFPE) to make blocks for cytologic analysis. Immunostaining was performed in a College of American Pathologists (CAP)-certified diagnostic IHC laboratory at the University of Washington following

standard protocols. In brief, unstained 4-micron sections were deparaffinized on an automated immunostainer (Bond III, Leica Biosystems). After rehydration, antigen retrieval, and peroxidase blocking, slides were incubated with 4 commercially available monoclonal mouse antibodies against CK7 (clone OV-TL12, DAKO), Uroplakin II (clone BC21, Biocare Medical), CD45 (clones 2B11 & PD7/26, DAKO), and Smooth Muscle Actin (clone 1A4, Cell Marque). Chromogenic detection was performed with a polymer anti-mouse poly-horseradish peroxidase-immunoglobulin G with 3,3-diaminobenzidine tetrahydrochloride chromogen and hematoxylin counterstain. Slides were reviewed by an expert genitourinary pathologist (M.T.). All positive and negative controls were appropriately stained.

H&E staining showed that urothelial brushes contain predominantly normal urothelial cells, which are characterized by oval nuclei and elongated spindly cytoplasm and often appear as orderly cell clusters. Umbrella cells, which are large polyploid cells that compose the superficial layer of the urothelium, were also observed. Degenerating urothelial cells with disrupted cytoplasm and pyknotic nuclei were occasionally present, corresponding to less than 1-2% of the entire cell block. CK7 staining was almost uniformly positive, confirming the epithelial nature of most brushed cells. Uroplakin II confirmed the presence of umbrella cells. CD45 showed the presence of few scattered positive lymphocytes, indicating minimal contribution to the brushed cells. Actin staining was negative, indicating no contamination with stromal cells.

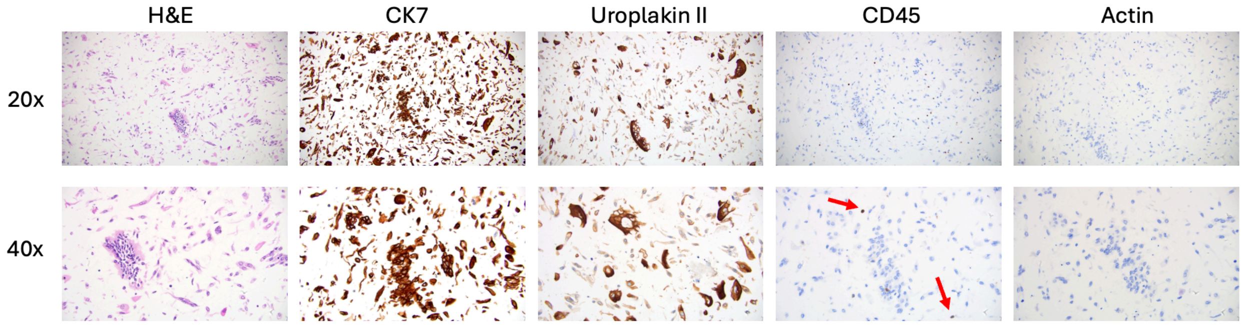

Supplementary Figure 11. Determination of cell composition of urothelial brushes by immunohistochemistry.
A representative urothelial brush from the bladder dome was fixed in Cytolyt, spun down, and embedded in paraffin to make a cell block. Slides sectioned from the cell block were stained with hematoxylin-eosin (H&E) to determine cell morphology, and immunostained with the following antibodies for specific cell types: cytokeratin 7 (CK7) for epithelial cells, uroplakin II specific for upper layer of urothelial cells (strongest in umbrella cells), CD45 for lymphocytes (scattered cells highlighted by red arrows) and actin for muscle cells. Representative pictures at magnification 200x (20x objective) and 400x (40x objective) are shown.

To formally assess the contribution of each cell type to the brushes, for each specimen we evaluated 12-14 fields of view (200x magnification) in the slides stained for CK7, actin, and CD45, and quantified the number of positive cells in each one (Supplementary Figure 12). Each specimen had more than 1600 cells evaluated, for a total of 8681 cells overall. None of the fields had any actin positive cell identified. The fraction of non-epithelial cells (all CD45 positive

cells) was below 0.06 in all fields, with an overall mean of 0.030 across specimens (minimum 0.017, maximum 0.038). These results indicate that urothelial brushes are composed mostly of urothelial cells with minimal contribution of lymphocytes and no stromal contamination.

**50-TR** (1670 cells evaluated)				
Field of view (200x)	CK7 positive cells	Actin positive cells	CD45 positive cells	Non-epithelial cell fraction
1	94	0	4	0.043
2	135	0	6	0.044
3	106	0	5	0.047
4	110	0	3	0.027
5	87	0	2	0.023
6	130	0	6	0.046
7	128	0	5	0.039
8	116	0	2	0.017
9	121	0	4	0.033
10	118	0	6	0.051
11	95	0	2	0.021
12	132	0	5	0.038
13	119	0	2	0.017
14	122	0	5	0.041
**mean**	**115**	**0**	**4.1**	**0.035**

**50-DO** (4505 cells evaluated)				
Field of view (200x)	CK7 positive cells	Actin positive cells	CD45 positive cells	Non-epithelial cell fraction
1	289	0	15	0.052
2	325	0	6	0.018
3	256	0	11	0.043
4	319	0	13	0.041
5	333	0	12	0.036
6	384	0	7	0.018
7	351	0	10	0.028
8	296	0	14	0.047
9	285	0	13	0.046
10	279	0	11	0.039
11	350	0	18	0.051
12	309	0	10	0.032
13	290	0	15	0.052
14	276	0	8	0.029
**mean**	**310**	**0**	**11.6**	**0.038**

**52-TR** (2506 cells evaluated)				
Field of view (200x)	CK7 positive cells	Actin positive cells	CD45 positive cells	Non-epithelial cell fraction
1	240	0	5	0.021
2	175	0	3	0.017
3	181	0	2	0.011
4	169	0	1	0.006
5	174	0	4	0.023
6	211	0	5	0.024
7	205	0	3	0.015
8	196	0	5	0.026
9	233	0	3	0.013
10	238	0	5	0.021
11	215	0	5	0.023
12	226	0	2	0.009
13	na	na	na	na
14	na	na	na	na
**mean**	**205**	**0**	**3.6**	**0.017**

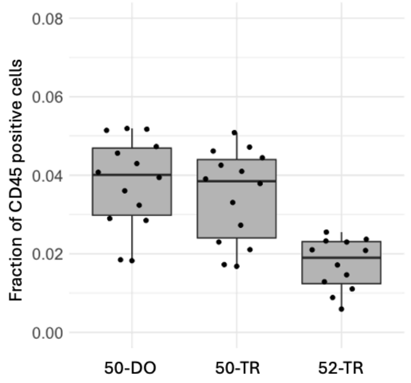

Supplementary Figure 12. Quantification of cell types in urothelial brushes.
For each specimen, immunostaining for CK7, actin, and CD45 was evaluated in 12-14 consecutive fields of view using an objective of 20x, for a total magnification of 200x. Top panel lists the counts by cell type and the non-epithelial cell fraction for each field of view and each sample. Bottom panel quantifies the fraction of lymphocytes (CD45 positive cells) for each sample. Boxplots display the quartiles with whiskers extending to the highest and lowest data points within 1.5 times the interquartile range.

Pathological assessment of epithelial brushes

As part of the routine pathological examination at autopsy, for all the donors in the study, a piece of bladder tissue was formalin fixed, paraffin-embedded and tissue blocks were sectioned and stained with H&E. H&E stained slides were reviewed to determine bladder pathology. All samples had minimal stromal infiltrating lymphocytes, consistent with the immunostaining findings above, and absent or small lymphoid aggregates which were not considered to significantly impact urothelial sampling (Supplementary Figure 13 A-C). Two donors showed

signs of fungal infection (Supplementary Figure 13 D-F), which was concordant with the gross pathological report from autopsy indicating cystitis (donor 10) and yeast infection (donor 35). Inflammatory cells (neutrophils) were present in the tissues but at low abundance and were not expected to contaminate the epithelial brushes significantly. Upon sequencing, the samples from donor 10, but not donor 35, showed a mutational profile indicative of potential artifacts and thus were discarded from downstream analyses (Supplementary Note 3). The rest of individuals presented with normal healthy bladder mucosa (not autolytic) although it appeared denuded in most FFPE slides, which is expected due to the fragility of postmortem urothelium.

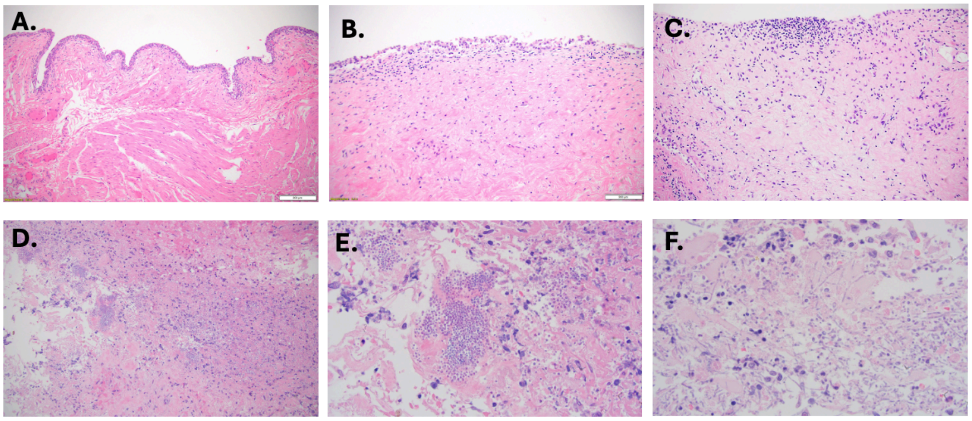

Supplementary Figure 13. Microscopic bladder findings from autopsy biopsies.
A-F. Representative pictures from H&E slides from FFPE bladder autopsy samples.
A-B: normal urothelium with absent or minimal infiltrating lymphocytes (A: donor 45, 10x; B: donor 47, 20x).
C: urothelium with small lymphoid aggregates of stroma and focally denuded mucosa (donor 19, 20x).
D-F: two cases with fungal infection (D: donor 10, 20x; E: donor 10, 60x; F: donor 35, 60x).

DNA Extraction

Epithelial brushes were removed from the tubes and samples were centrifuged at 2250-3000g for 10 min at 4°C. Supernatant was carefully removed and DNA was extracted from the cell pellet using the DNeasy Blood & Tissue (Qiagen). Cell pellets were resuspended in 180 µl of buffer ATL by carefully pipetting up and down several times before transferring to 1.5 ml Eppendorf LoBind tubes (Axygen). Then 20 µl of proteinase K were added and samples were incubated at 56°C for 10 min while shaking. If tissue was not fully digested, incubation time was increased until digestion was complete. Then incubation with 4 µl of RNase A (100 mg/ml) for 2 min at room temperature was performed to eliminate RNA from samples. The rest of the protocol followed manufacturer's instructions with the exception of the elution, which was performed in 50 µl of IDTE buffer (IDT) followed by a second elution with 25 µl. DNA was quantified with a Qubit HS dsDNA kit (ThermoFisher Scientific). DNA yield was highly variable with an average of 5.8 µg (min 359 ng, max 20.9 µg). Agilent 4200 TapeStation Genomic tapes were used to determine the DNA Integrity Number (DIN) for each sample. DINs were low as expected given the nature of the samples but we demonstrated that the mutation density of the

samples was not associated with the DIN number (Supplementary Table 4, Supplementary Note 9). We also demonstrated that the distribution of DINs was comparable in males and females and the dN/dS of the genes that showed increased positive selection in males was not related to DIN (Supplementary Note 9).

Capture Panel Design

A panel containing 16 genomic regions was designed based on the literature and COSMIC data to represent the subset of most commonly mutated genes in normal bladder and bladder cancer while keeping the size of the panel reasonably small for ultradeep DNA duplex sequencing (111,876 bp total footprint, Supplementary Tables 2 and 3). The panel included 12 genes for which most of the coding region was sequenced (*ARID1A, NOTCH2, FOXQ1, CDKN1A, KMT2D, RB1, CREBBP, TP53, EP300, KDM6A, RBM10* and *STAG2*), 3 genes for which only selected regions were targeted due to clustering of cancer mutations in those regions and/or difficulties for capturing the full gene (*PIK3CA, FGFR3,* and *KMT2C*), and the *TERT* promoter, which is mutated in 70-80% of bladder cancers[2,9,10]. The panel was built by TwinStrand Biosciences (Seattle, WA) using biotinylated 120 bp DNA probes with partial probe overlap and rebalancing to maximize homogeneous capture. BED files representing the regions included in the panel were built using UCSC table browser with hg38 and selected transcripts from Supplementary Table 2 plus TERT promoter. BED files to filter regions with enough coverage after sequencing were generated as part of the mutation calling pipeline (see Supplementary Note 3).

Duplex Sequencing Library Preparation and Sequencing

Duplex sequencing libraries were prepared using commercially available kits (TwinStrand Biosciences, Seattle, WA) and 250ng of genomic DNA. DNA was fragmented using the enzymatic fragmentation kit module following the manufacturer’s protocol with the exception of the time of incubation at 25℃, which was kept at 40 min only for samples with DIN>=5. For samples with lower DINs, incubation time was reduced to 5-30min (depending on DIN) to avoid overfragmentation. For each sample, fragmented DNA was run in the Agilent 4200 TapeStation with HS D1000 tapes to confirm average fragment sizes between 300-500bp. If the DNA was not sufficiently cut, additional enzyme was added and the mix was incubated longer. Properly fragmented DNA was then subject to end-repair, A-tailing, ligation to duplex sequencing adapters, library conditioning, and PCR amplification, according to protocol. After PCR product clean up, hybridization capture was performed at 65℃ for 16-20 hours using the designed capture panel (Supplementary Tables 2 and 3). After washes and PCR amplification, libraries were quantified using the Qubit dsDNA HS Assay kit (ThermoFisher Scientific) and fragment size was confirmed using Agilent 4200 TapeStation with HS D1000 tapes. Libraries were then diluted and pooled for sequencing. Sequencing was performed with a NovaSeq 6000 at the Department of Laboratory Medicine and Pathology at University of Washington or a NovaSeq X Plus at Novogene or the Fred Hutchinson Cancer Center using 2 x 150 bp paired-end reads (~115M reads/sample). The average duplex depth of the samples ranged from 1,236x to 9,303x with a median of depth across samples of 5,164x (Supplementary Table 4).

Supplementary Note 3: Mutation calling

Abstract

This Supplementary Note describes the process of mutation calling on DNA duplex sequencing data. It describes a novel computational pipeline (deepUMIcaller) designed for this purpose, and explains its use in the present study. In particular, it describes the rationale behind a set of post-calling filters implemented to rid the calls from potential sequencing and alignment artifacts.

deepUMIcaller

We designed and implemented an end-to-end computational pipeline (deepUMIcaller; github.com/bbglab/deepumicaller) that receives DNA duplex sequencing FASTQ files and outputs VCF files with mutations identified across sequenced samples. deepUMIcaller is based on an early version of nf-core/fastquorum pipeline, implementing the `fgbio` Best Practices FASTQ to Consensus Pipeline (https://github.com/fulcrumgenomics/fgbio/blob/main/docs/best-practice-consensus-pipeline.md), and a variant calling based on `VarDictJava` (https://github.com/AstraZeneca-NGS/VarDictJava). A series of filters to discard potential artifacts are included in the pipeline. In the following paragraphs, we describe the use of this pipeline to call mutations on the ultradeep sequenced urothelium samples included in this project. All parameters described correspond to this work, but are configurable by the users of the pipeline.

Raw reads are first processed by the pipeline to extract the first 8 bp corresponding to the unique molecular identifier (UMI) that tags each read and an additional spacer bp. An additional step of preprocessing consists of removing the first 10 bps following the tag. This removes known artifacts associated with the beginning of the reads[11]. The processed reads are then aligned using bwa-mem[12] v0.7.17 to the GRCh38 reference genome (https://lh3.github.io/2017/11/13/which-human-reference-genome-to-use), masking known artifactual regions[13,14] of this genome assembly using BEDtools[15].

The `GroupReadsByUMI` command from `fgbio` is then used by the pipeline to group aligned reads that share the same duplex consensus tag and genomic coordinates. PCR duplicates originating from each DNA strand of a unique DNA fragment are first grouped together, and then the two groups (corresponding to the two strands of a unique DNA fragment captured by the library, if available) are brought together to form a read family. The requirements for a family to be formed are a minimum mapping quality of 10 for all member reads and, at most, 1 nucleotide difference in the duplex tag sequences of the different member reads (*`--edits 1 --min-map-q 10`*). Then every family of reads is used to build a consensus read from its members. Bases conserved across all reads of the family are present in the consensus, while positions with variable bases across reads may be converted to Ns or called with the preponderant base, depending on the ratios of mismatching bases at one position (see below). To build consensus reads from the read families, the pipeline uses the `CallDuplexConsensus` command from `fgbio`. Only reads with a minimum base quality of 20

are used to build a duplex consensus. The consensus sequence of the family is defined taking advantage of the overlapping bases of all family members. This is achieved through (*--min-reads 1 1 0 --min-input-base-quality 20 --consensus-call-overlapping-bases true*). All consensus reads are then realigned to the genome, and the consensus reads resulting from the same original DNA molecule are built into a duplex consensus read. From this point onwards, we distinguish between duplex consensus reads and the original reads produced by the sequencing machine, to avoid confusions.

To remove potential artifactual mutations introduced by reads with ambiguous mapping to the genome, the pipeline uses the difference between the score of the read's best alignment (AS) and the score of its second best alignment to any other position in the genome (XS). If for the two reads of a pair this difference is less than 50 units, the read pair is discarded. If one of the members of the pair shows an AS-XS smaller than 50, while the other shows AS-AX >= 50, both reads are kept. After filtering for mapping uniqueness, the pipeline only keeps the primary alignment of properly paired reads for downstream calling (*samtools view <bam> -b -h --require-flags 0x2 --exclude-flags 0x900*). Read pairs with one of the members incorrectly mapped are discarded. The resulting BAM file (***BAM_unique***) contains all consensus reads formed as explained above including those which do not form duplex reads (as explained below).

Duplex consensus reads are built from PCR duplicates, i.e., reads bearing the same UMI and aligned at exactly the same genomic position. In this process, we allow at each position of the duplex consensus no more than one out of ten PCR duplicates with a conflicting nucleotide. If this threshold is surpassed, an N is introduced in the sequence of the consensus duplex read. This is achieved by setting the parameter `max-base-error-rate to 0.1`.

The duplex consensus reads are filtered into three groups with different confidence, based on the number of raw reads derived from each DNA strand.

1. **High quality duplex reads** are those composed of families formed with at least 3 PCR copies of both strands of the original DNA fragment,
2. **Medium quality duplex reads** are composed of families formed with at least 2 PCR copies of both strands of the original DNA fragment,
3. **Low quality duplex reads** are composed of families formed with at least one PCR copy per strand.

These three sets of reads are concentric, with the medium quality reads containing the high quality reads and the low quality reads containing the medium quality reads. None of these three sets of reads includes consensus reads that do not have supporting evidence from both strands.

To obtain the reads fulfilling these three different levels of quality, the following command is applied:

High: `FilterConsensusReads --min-reads 6 3 3 --min-base-quality 30 --max-base-error-rate 0.1 0.1 0.1 --max-no-call-fraction 0.2 --require-single-strand-agreement true;`
Medium: `FilterConsensusReads --min-reads 4 2 2 --min-base-quality 30 --max-base-error-rate 0.1 0.1 0.1 --max-no-call-fraction 0.2 --require-single-strand-agreement true;`
Low: `FilterConsensusReads --min-reads 2 1 1 --min-base-quality 20 --max-base-error-rate 0.1 0.1 0.1 --max-no-call-fraction 0.2`

While we keep these three sets of duplex consensus reads and the mutations called using each to gather internal metrics, only the **Medium** set is used in all analysis described in the main manuscript. We made this decision as a compromise between the number of mutations called and the potential rate of errors from these sets of reads. Note that the 3 thresholds produced very similar mutational profiles (Supplementary Fig. 14), indicating that the medium and low thresholds did not introduce mutations shifting the profiles, as artifacts would be expected to do.

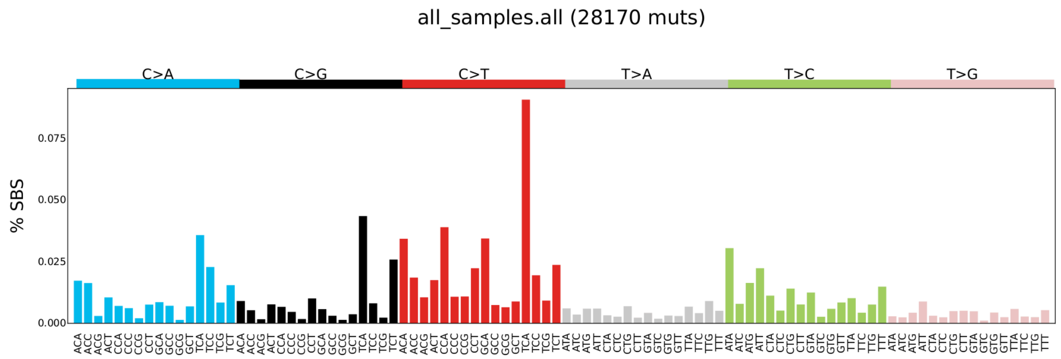

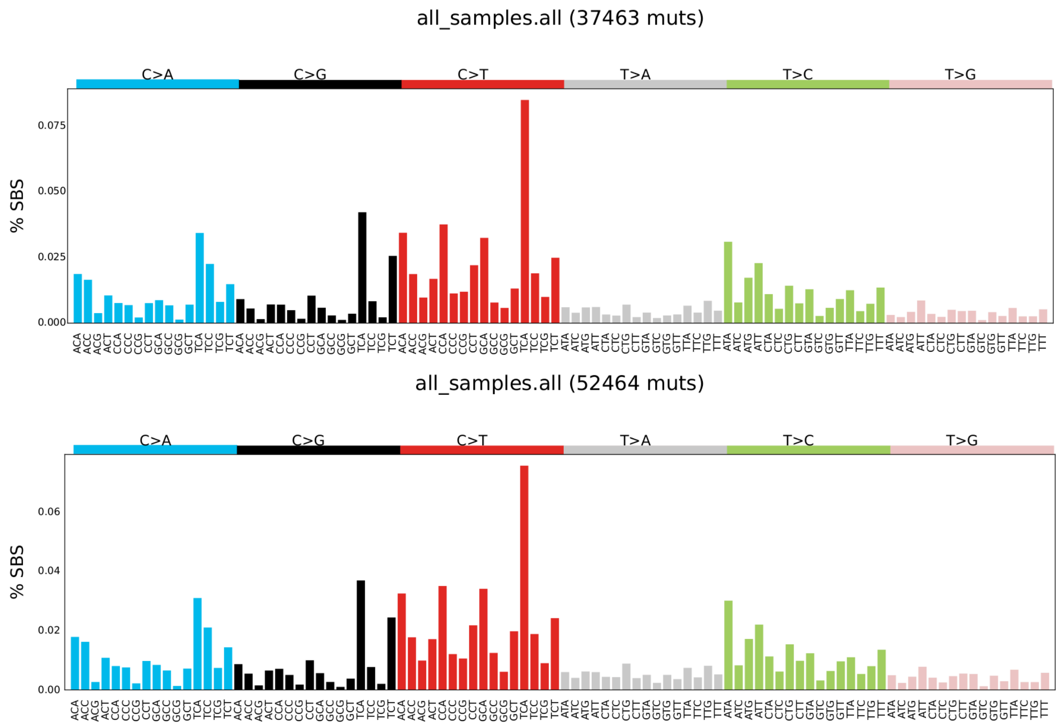

Supplementary Figure 14. Mutational profile of the mutations identified on three sets of reads with different levels of duplex support.
Top: high quality reads; middle: medium quality reads; bottom: low quality reads.

As a result, three new BAM files with the reads resulting from the three quality filters are obtained. The variant calling is then performed on these three BAM files. We will refer to these BAM files as ***BAM_filtered***.

To avoid overlapping read pair ends interfering with the counting of mutations and depth these are hard clipped using `ClipBam` (*`--clipping-mode Hard --clip-overlapping-reads true --clip-bases-past-mate true --auto-clip-attributes true`*). Genomic regions covered by at least one duplex read are kept to ensure that the variant calling takes place in all covered regions, including those outside the areas targeted by the panel. Upon variant calling, this provides an important number of non-protein affecting mutations, essential for some downstream analysis, such as the calculation of mutational signatures and the comparison of protein affecting and non-protein affecting mutation density.

`VarDict-java` v1.8.3 is then used by the pipeline for the variant calling in `pileup` mode, reporting the mutational status of all positions in the previously generated BED file using:

```
vardict-java -c 1 -S 2 -E 3 -g 4 -f 0.0 -r 1 -m 9999 -P 0 -p -z 1 -o 0.5
-L 100 <BEDfile>
```

This command yields all variants (SNVs and indels up to 100bp) in each sample.

The output is filtered with the recommended VarDict filters (*teststrandbias.R | var2vcf_valid.pl -A -E -f 0.0 -p 0 -m 20 -v 2*), and only mutated positions were kept in the final VCF file.

From the VarDict calling, a number of duplex reads supporting the reference and alternate alleles can be computed, and thus, the variant allele frequency (VAF) of the variant can be calculated as the number of alternate reads divided by the number of total reads covering the position. We call this VAF **VAF VD** to distinguish it from different calculations of VAF in downstream analyses.

Next a post-calling analysis of the mutations is carried out to curate them with additional information, relevant for the detection of sequencing, mapping and calling artifacts, as well as to filter out germline variants. This analysis uses `samtools mpileup` to have full control over the reference and alternate read counts, instead of relying on the numbers provided by VarDict upon mutation calling. This command is applied to the *BAM_filtered* files. Hence, for each mutation, the number of duplex consensus reads supporting the reference and alternate bases is obtained. This command is also applied, in parallel, to the *BAM_unique* file. This yields the number of consensus reads (either forming duplex consensus or not) supporting the reference and alternate allele at each mutated position. Finally, an “all molecules non duplex” (that is, accounting only for consensus reads that do not form families with enough quality to produce duplex consensus reads) number of reads supporting the reference and alternate bases can also be calculated.

Then, three values of variant allele frequency (VAF) for each mutation are calculated. The quotient of duplex consensus reads (at a given quality filter) supporting the alternate allele and all duplex consensus reads covering the mutated position is called simply **VAF.** The quotient of all consensus (duplex or single-strand) reads supporting the alternate allele and all consensus reads covering the mutated position is called all molecules VAF (**VAF AM**). Finally, the quotient of all molecules non duplex reads (that is, single strand consensus reads and other consensus reads that do not meet the criteria for duplex) supporting the alternate allele and all molecules non duplex reads covering the mutated position is called non duplex VAF (**VAF ND**).

A comparison of the VAF computed using duplex consensus reads counted by VarDict or using the `samtools mpileup` shows a high degree of agreement between the two (Supplementary Fig. 15).

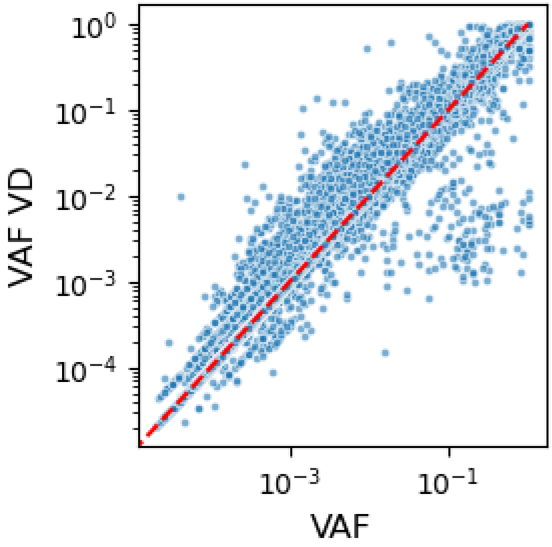

Supplementary Figure 15. Comparison of VAF from duplex reads counted with VarDict (VAF VD) or samtools mpileup (VAF).

As mentioned above, when there is a disagreement between more than 1 out of 10 PCR copies within a family, an N is placed at that position of the duplex consensus read, as a token of that disagreement. We reasoned that genomic positions with an unexpected number of Ns in the duplex consensus reads covering them could reflect sites with high rates of sequencing artifacts, arising from causes such as DNA damage[11,16].

Thus, the pipeline defines a sample-specific threshold to mark positions with a fraction of Ns higher than expected (*n_rich* positions). The rationale to do the calculation per sample is that the number of N-rich positions may vary across samples, as different samples may be subject to different insults damaging the DNA and resulting in sequencing errors. The threshold was defined as follows:

1. **Select positions to compute the expected N distribution.** Select all genomic positions in the panel and filter out those with duplex consensus read coverage below 25, to avoid biasing the calculation due to positions with very low coverage. Out of all remaining positions (with depth above 25), we select those at the top quartile of consensus duplex read depth to compute the expected distribution of Ns.
2. **Compute the fraction of Ns at all selected positions.** This is computed as the number of Ns at a position divided by the total number of reads covering the position.
3. **Log-transform the data.**
4. **Define as n_rich all positions exhibiting the median + 2*standard deviation of the distribution, or higher fraction of Ns.**

Supplementary Figure 16 illustrates the calculation of N-rich positions in one exemplary sample in the cohort, across both all genomic positions with coverage above 25 duplex consensus reads and mutated positions.

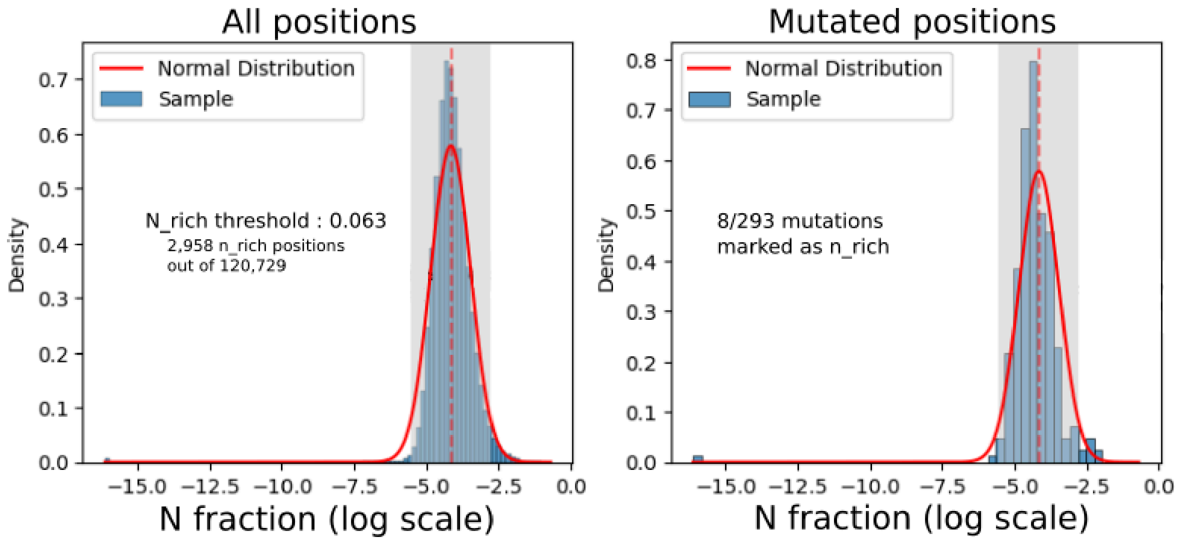

Supplementary Figure 16. Distribution of the fraction of Ns across all (left) or only mutated (right) positions.
The distribution of the fraction of Ns is presented across all genomic positions (left) or only mutated positions (right) in an exemplary sample of the cohort under study. The normal distribution (red line) in the left and right plot is the same, and it is estimated from all positions. The number of N-rich positions according to the criteria described above is indicated in each graph.

Additionally, the pipeline labels as *low_mappability* all mutations overlapping ENCODE blacklisted regions, taken from the ENCFF356LFX dataset (https://www.nature.com/articles/s41598-019-45839-z#data-availability). Mutations falling in RepeatMasker labeled positions are labeled as *low_complexity*.

The mutation calling pipeline produces three VCF files of SNVs and short indels (up to 100bp) for each sample, together with the three *BAM_filtered* files used for the calling that will contribute to the posterior analysis (with each BAM-VCF pair corresponding to one of the three quality thresholds discussed above).

Checking for artifacts
As some sequencing artifacts related to DNA damage and end-repair are known to affect the ends of the reads more frequently, we checked the distribution of all mutations along duplex consensus reads to check for potential end-of-reads artifacts. Initially we observed a slightly higher presence of mutations at the beginning of the reads, as reported previously[11] Nevertheless, after clipping the first 10bp of reads this increment disappeared (Supplementary Fig. 17). Moreover, the fraction of C>A changes, those that are more frequent upon oxidative damage caused to guanines, appeared fairly similar across all read positions.

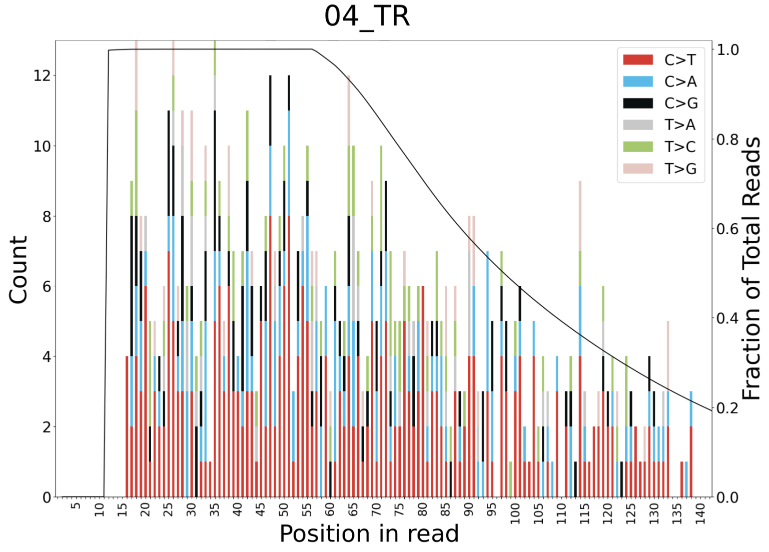

Supplementary Figure 17. Number and type of mutations identified at different positions of duplex consensus reads.
The number and type of mutations at different positions of the reads in an exemplary sample of the cohort is shown. Note that the type and number of mutations are homogeneously distributed along the read position as expected in the absence of end-of-read mutational artifacts.

Upon visual inspection of the mutational profile of all samples in the cohort, we identified two samples from the same donor that presented a profile that appeared very similar to that of SBS18, which is caused by reactive oxygen species, and is also very similar to reported potential duplex sequencing artifacts (Supplementary Fig. 18). Of note, this sample corresponded to a donor with cystitis, which is a bladder condition related to oxidative stress[17]. These two samples were subsequently removed from the cohort. The mutational profile of other samples were very similar to that of the pooled cohort which, in turn, was very similar to that obtained through whole-genome sequencing of clonal or quasi-clonal structures of normal bladder[18] (see Supplementary Note 5).

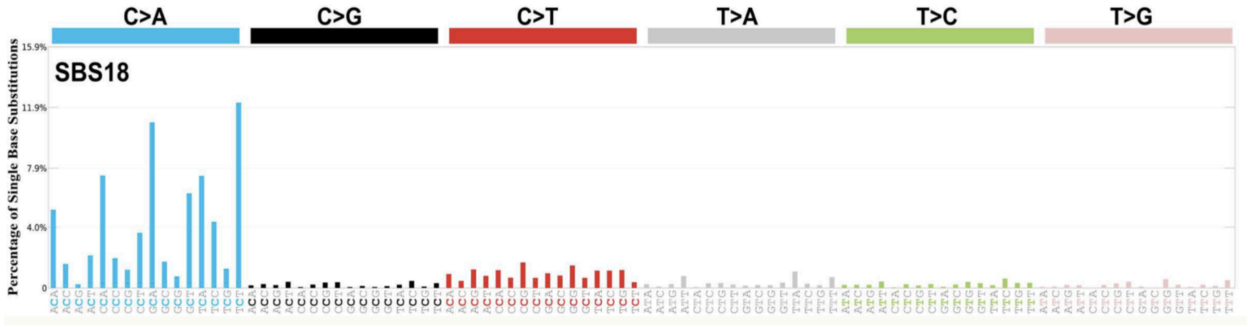

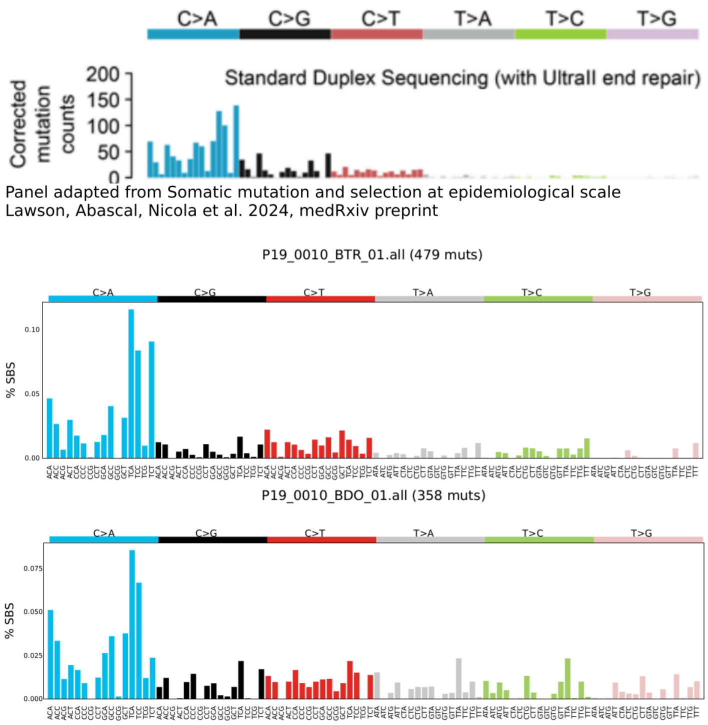

Supplementary Figure 18. Mutational profile of the two samples of donor 10.
The mutational profile of SBS18 and reported potential DNA duplex sequencing artifacts[11] is also shown above for comparison.

Filters

From the VCF files of each sample, we flagged the following variants as candidates to be filtered out:

1. Variants in positions flagged as *N-rich* in the sample or in more than 10% of the samples of the cohort,
2. Variants not supported by the reanalysis of the variant calling with `samtools mpileup` described above,
3. Variants in low mappability areas,
4. Variants in not properly covered areas (at least 190x in 80% of the samples),
5. Variants with a gnomAD allele frequency > 0.1 (source Ensembl 111),
6. Variants with duplex depth below 100,
7.Variants with VAF_AM value that is 3 times or more its duplex VAF (defined based on exploration of Supplementary Fig. 19),
8. Variants with any of the three VAF metrics equal to or greater than 0.3 (likely germline).

Dots represent variants identified in different samples, and the VAF and VAF AM calculated for each of them. While the VAF AM of the vast majority of SNVs (38,030/39,925) is highly concordant with that obtained using only duplex consensus reads (within VAF +/- 3*VAF, as indicated by the blue and green lines), for a minority of variants (in general with low VAF), the

VAF AM is more than 3 times the value of VAF. These cases are likely recurrent artifacts of the calling that form pseudo-duplex mutations; that is, damage in one strand of the DNA that is copied to the opposite strand during the step of end repair and nick filling in the library preparation protocol or at the first round of PCR. This suspicion is supported by the fact that the vast majority of these variants are located at N-rich positions (orange dots). There are few other N-rich variants (removed by filter number 2). These remaining unfiltered SNVs are very rare and can be easily filtered out through this comparison (filter number 7 in the list above). We defined this criteria with SNVs and applied across variants for consistency.

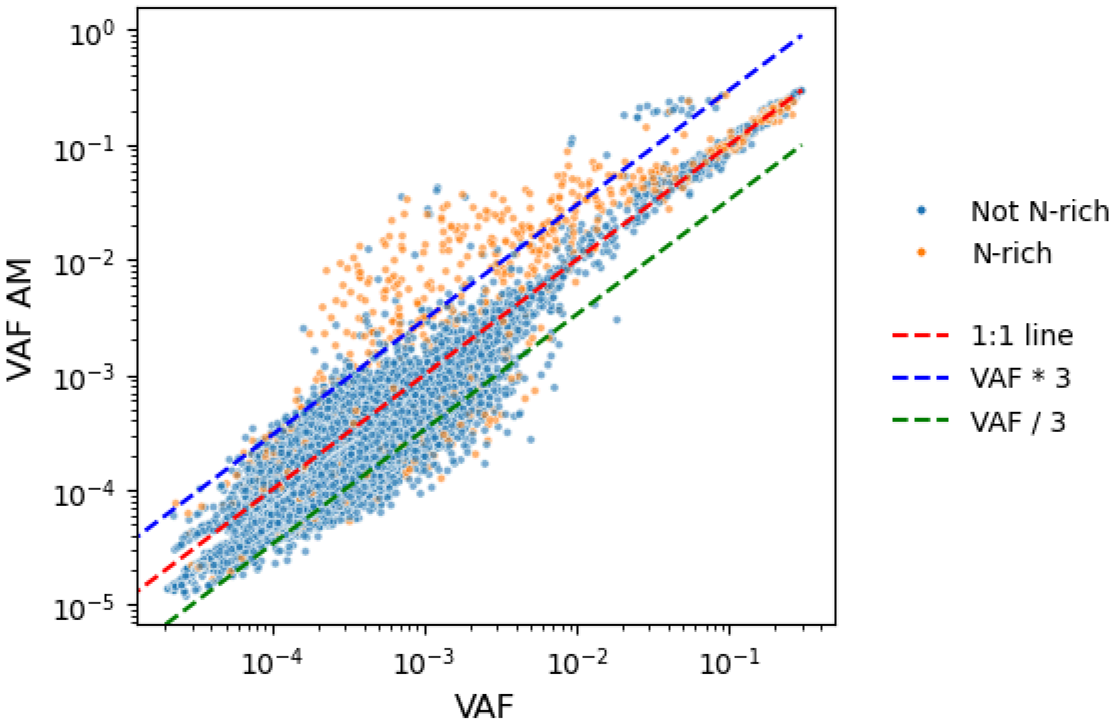

Supplementary Figure 19. Comparison of VAF and VAF AM across the cohort.
Every dot represents a SNV identified in a sample, represented by their VAF and VAF AM values. The dots are colored according to whether or not they overlap N-rich positions in the panel. The plot shows that SNVs at N-rich positions are overrepresented across those whose VAF AM is distorted. This suggests that N-rich positions correspond to recurrent sequencing artifacts, probably caused by DNA damage, which can constitute pseudo-duplex reads, and must therefore be filtered out.

Below, we show the impact of the application of the 8 aforementioned filters to the variants detected in the 79 samples analyzed in the cohort.

1. Applying FILTER:notcontains n_rich filter implied going from 113,317 mutations to 100,920 mutations.

Applying FILTER:notcontains cohort_n_rich_threshold filter implied going from 100,920 mutations to 99,959 mutations.

Applying FILTER:notcontains cohort_n_rich filter implied going from 99,959 mutations to 95,042 mutations.

2. Applying FILTER:notcontains no_pileup_support filter implied going from 95,042 mutations to 91,316 mutations.
3. Applying FILTER:notcontains low_mappability filter implied going from 91,316 mutations to 91,257 mutations.
4. Applying FILTER:notcontains not_covered filter implied going from 91,257 mutations to 70,393 mutations.
5. Applying FILTER:notcontains gnomAD_SNP filter implied going from 70,393 mutations to 65,824 mutations.
6. Applying DEPTH:ge 100 filter implied going from 65,824 mutations to 65,790 mutations.
7. Applying VAF_distorted_expanded_sq:False filter implied going from 65,790 mutations to 65,570 mutations.
8. Applying VAF:le 0.3 filter implied going from 65,570 mutations to 64,286 mutations.
   Applying vd_VAF:le 0.3 filter implied going from 64,286 mutations to 64,282 mutations.
   Applying VAF_AM:le 0.3 filter implied going from 64,282 mutations to 64,278 mutations.

To understand how these filters affect the distribution of the three values of VAF computed from the reads counted using the `samtools mpileup`, we compared the distributions of duplex VAF (VAF), all molecules VAF (VAF AM) and all molecules non duplex VAF (VAF ND). As explained above, we found that a group of variants (most of them N-rich) exhibited a more than 3-fold increase in the VAF AM with respect to the VAF (Supplementary Fig. 20). Variants with the highest distortion in VAF (ratio between VAF AM and VAF) were particularly enriched for some tri-nucleotide changes, giving further support to the suspicion that many of them constitute artifacts likely introduced during the library construction (Supplementary Fig. 20). The application of filter number 7 removed this group of variants (Supplementary Fig. 20) and eliminated the observed differences in frequency of tri-nucleotide changes across the 96 channels for all remaining variants (Supplementary Fig. 20).

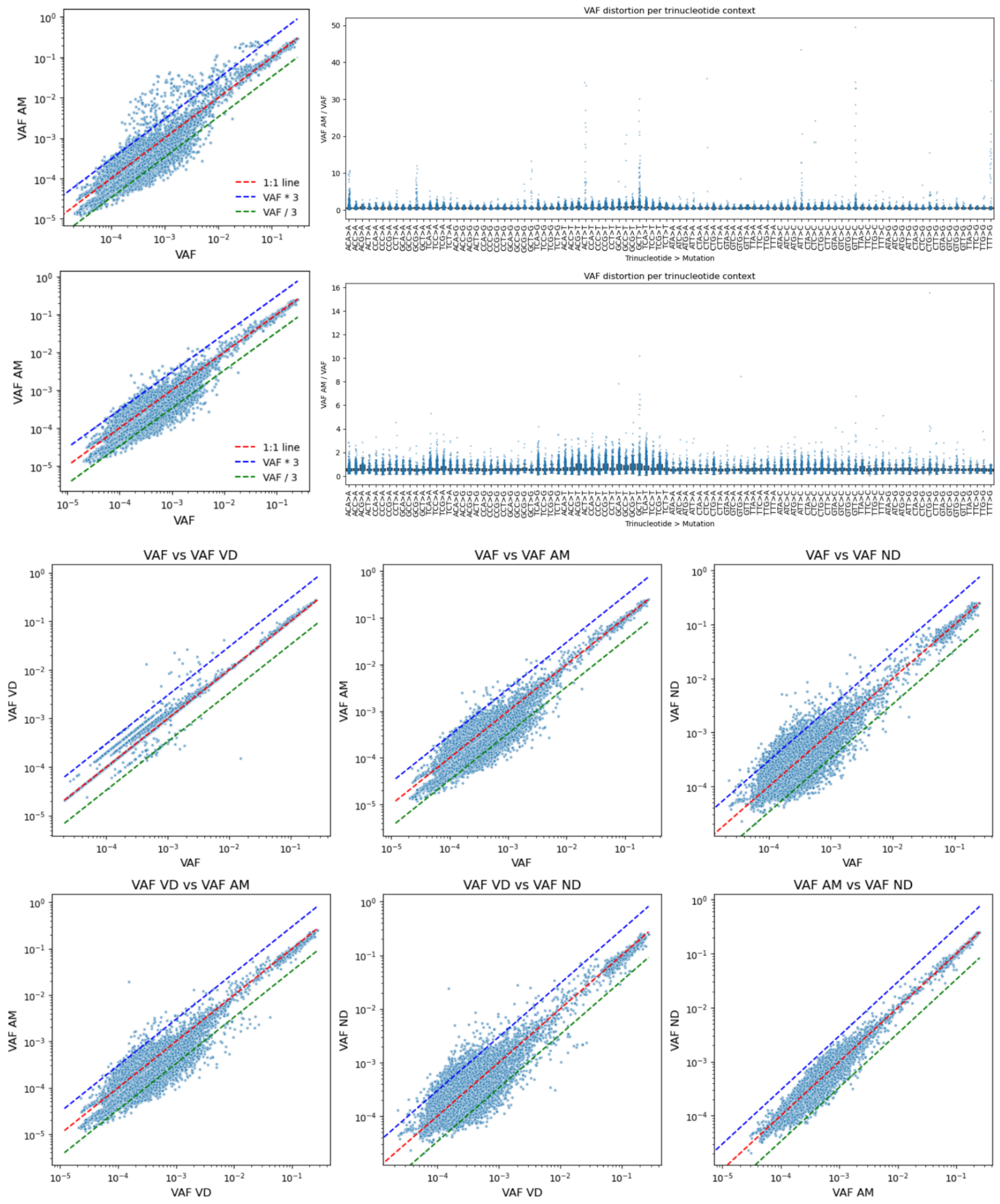

Supplementary Figure 20. Comparison of different calculations of VAF of variants across samples before and after the application of filters.
Explanation in the text.

The distribution of duplex VAF and alternate duplex consensus read counts of all variants identified in the 79 samples are shown in Supplementary Figure 21. As expected, the majority of variants are supported by just one duplex consensus read, and present VAF below 10^{-3}. However, some variants (driving the expansion of bigger clones, and/or very recurrent within samples due to convergent evolution caused by positive selection) rise to around 1-10% VAF.

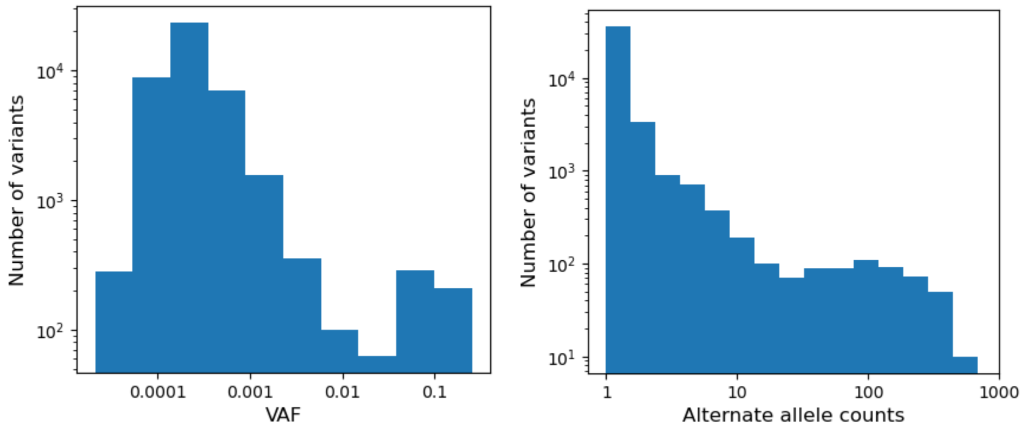

Supplementary Figure 21. Distribution of variant duplex VAF and alternate duplex consensus read count.

Cross-contamination analysis

We explored the level of potential contamination between samples in the cohort. To this end, we identified all germline variants in each of the samples that were at a VAF > 0.3. We then compared these sets of germline variants between samples to identify, for each pair of samples, which mutations are germline in only one of them. These are the variants that are useful for identifying hypothetical contamination between samples. The only mutation that we excluded from the analysis of contamination is a mutation in one hotspot of the TERT promoter, which was identified as a SNP across several samples and was detected as a somatic mutation with a variant allele frequency above 0.01 in 8 samples. This mutation is likely a recurrent somatic mutation that is present at high VAF in some samples. For each pair of samples we then compared all the non-germline variants to the set of susceptibly contaminating variants and computed the proportion of how many of those are present in each sample.

We reasoned that if a contamination from one sample (contaminant) was present in another (contaminated), we expected to find all germline variants of the contaminant within the contaminated, either as already germline or as somatic mutations. This would be consistent with a small portion of DNA from the contaminant present in the contaminated. Moreover, all the germline variants of the contaminant would be observed at roughly the same variant allele frequency in the contaminated. Therefore, we next looked at the fraction of germline variants across samples that are identified as somatic mutations across other samples (Supplementary Fig. 22).

We found one sample (the dome of donor 38) with more than half (0.6) of the germline variants identified in another donor (9) as somatic mutations. Yet, there are 4 private SNPs of donor 9 that are not identified as somatic mutations in this sample, casting doubt on whether a sample from donor 9 is really a contaminant of the sample from donor 38. This is still a possibility, since the 4 SNPs could be missing due to chance in the detection. While this is the only case in which cross-contamination could conceivably have occurred in the cohort, we conservatively decided to filter out all somatic mutations detected across samples that are germline variants in another donor. The only exception was a mutation in one hotspot of the TERT promoter, as discussed above.

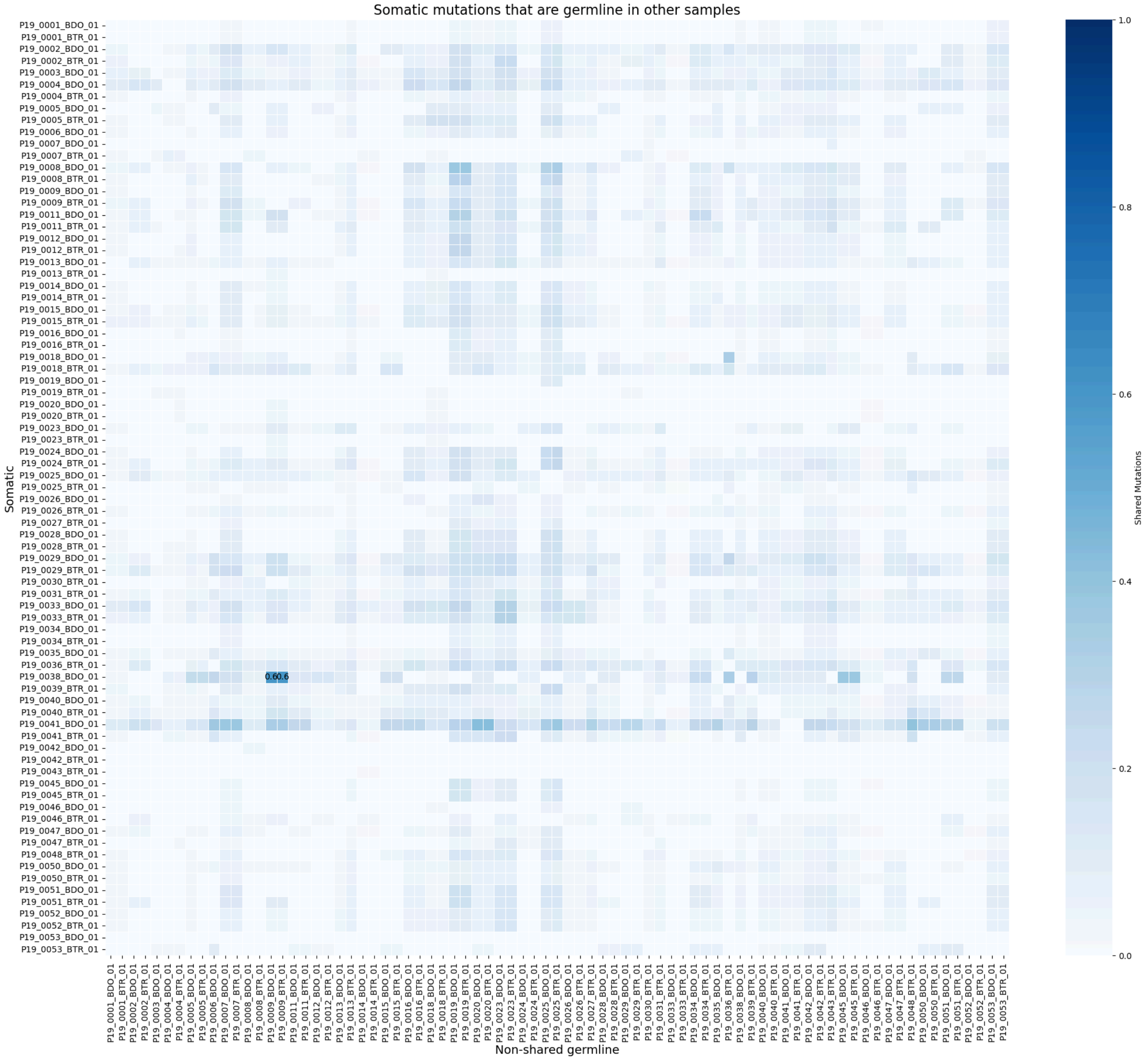

Supplementary Figure 22. Germline variants identified as somatic mutations in other samples.

The values in the heatmap represent the proportion of germline variants of a sample (in columns) that have been identified as somatic mutations in another sample (in rows). The proportion is computed taking into account the number of non-shared germline variants to obtain a fair comparison.

Ultradeep sequencing

The study aimed for a sequencing depth of ~5,000x in each sample analyzed, and the starting amount of DNA and specific library preparation parameters (Supplementary Note 2) were adjusted for this goal. In duplex sequencing, each duplex read corresponds to an original DNA molecule, therefore our target depth enables us to sample mutations present in less than 1 in 1,000 cells (VAF below 10^{-3}). The mean depth obtained for each sample is indicated in Supplementary Table 4.

The targeted panel was carefully designed, balancing the location of probes and their relative amounts to produce a coverage as even as possible across all captured regions. Nevertheless, differences in depth across regions were expected, due to variation in the efficiency of capture between the genomic regions included in the panel (Extended Data Fig. 1b). The aggregated depth across genes ranged from 277,337x (KDM6A) to 578,908x (CDKN1A). Even within every gene, differences in the coverage per exon could be detected (Extended Data Fig. 1c). Moreover, the sequencing coverage within exons also showed variability across different positions (Supplementary Fig. 23).

The differences in coverage across genomic positions in a gene, across genes in a sample, and across samples affect essential downstream analyses, such as the calculation of positive selection and the estimation of the fraction of urothelium with driver mutations. Indeed, these differences affect the probability of detecting mutations, and the expectation of mutations under neutrality at different genomic positions, different genes and different samples. In particular, this motivated us to design (or adjust) robust methods for the calculation of positive selection to account for the position-specific sequencing coverage (Supplementary Note 5).

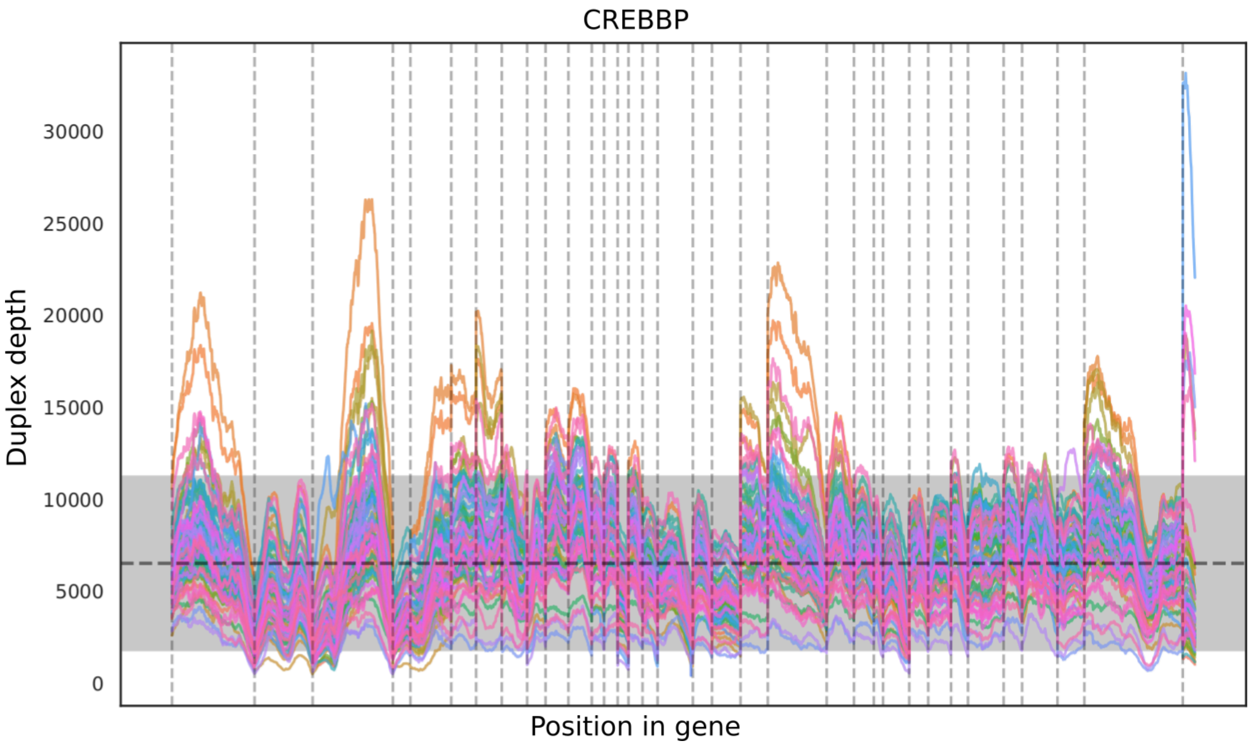

Supplementary Figure 23. Duplex depth (number of reads) at each position of the coding sequence of CREBBP included in the panel.

Each color line corresponds to a sample. Vertical lines indicate exon boundaries. The horizontal line indicates the median depth in the coding region, and the shaded area corresponds to the interquartile range. See also Supplementary Table 4.

Supplementary Note 4: Estimation of the error rate of the technology

Abstract

To calculate the error rate of the DNA duplex sequencing technology employed in this study, we compared the density of mutations identified in three cord blood samples with that expected from the observations of two studies that carried out whole genome sequencing of colonies derived from hematopoietic stem cells[19,20]. This study yielded an error rate of ~4x10^{-8}, two orders of magnitude lower than the average mutation density in the cohort, ensuring that the proportion of errors is low. Several orthogonal analyses carried out on the samples of our cohort corroborate the presence of a low level error rate in the cohort.

Estimation of the error rate of the technology in cord blood samples

We estimated the error rate intrinsic to the duplex sequencing technology employed to detect mutations in this study. To that end, we constructed DNA duplex libraries using the same kits and the same capture panel as in this study for three cord blood samples obtained from newborns (StemCell; Extended Data Fig. 2a). The samples were sequenced to depths of 2,985x, 4,608x and 4,308x. We identified 35, 26 and 47 SNVs in these three samples, accounting for mutation density of 6.1x10^{-8}, 3.1x10^{-8} and 5.7x10^{-8} mutations per sequenced base (Extended Data Fig. 2a). We obtained the average number of mutations observed in newborn blood from two studies[19,20], based on the expansion of clones from hematopoietic stem cells. According to these two studies, the number of somatic SNVs in cord blood is between 60 and 105, yielding an expected mutation density between 2x10^{-8} and 3.5x10^{-8}, considering a haploid whole genome.

Comparing the rate of mutations detected in these three samples with that expected in newborn blood, we estimated that the rate of errors introduced by the technology using these three samples is not greater than 4.1x10^{-8} (Extended Data Figure 2a).

Comparison with the mutation density of samples

The importance of the error rate must be evaluated in comparison with the mutation density of the samples analyzed (Extended Data Fig. 2a). The errors introduced by the technology become a problem if they are an important part of the mutations observed in the samples under evaluation. Thus, we next compared this error rate calculated in cord blood with the mutation density obtained across the samples of the cohort. Across samples, this error rate is, in most cases below 10%, with the lowest being 2%. In a couple of samples with very low mutation density, the error rate is comparable to 20% of the mutation density.

Mutational profile of samples with different mutation density

This comparison is based on the error rate calculated in cord blood, a scenario in which we do not expect positive selection, in contrast to what we observe in the normal urothelium of adults. In addition, the type of sampling is different in both scenarios. Thus, to understand whether this rate of errors posed a problem in its comparison with different mutation density across samples,

we inspected the mutational profiles of the two samples with the lowest mutation density (01_DO and 52_DO) and the two samples with the highest mutation density (02_DO and 02_TR), and compared them with the mutational profile of the pooled cohort. We reasoned that a higher prevalence of artifacts in the samples with lower mutation density should be reflected through the observation of a more different mutational profile. However, the comparison reveals that the profile of these samples, despite their low mutation density (less than 1 mut/Mb) is remarkably similar to that of the pooled cohort (cosine similarity = 0.82 and and 0.8, respectively; Supplementary Fig. 24).

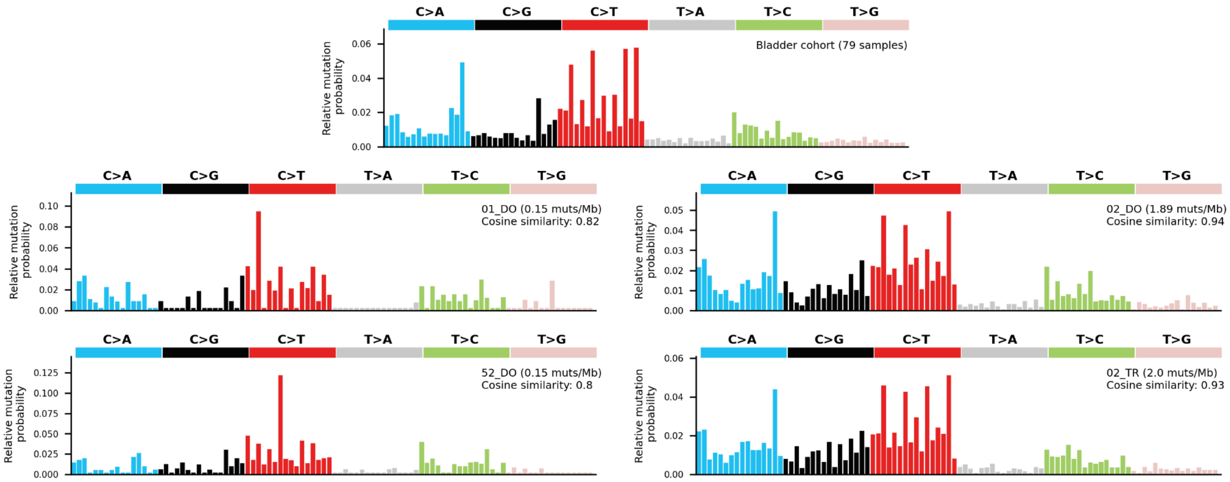

Supplementary Figure 24. Mutational profile of the pooled cohort (top) and the two samples with the lowest (left) and highest (right) mutation density.
Cosine similarity with the mutational profile of the cohort is shown for each sample.

There were only two samples whose mutational profile clearly differed from the rest, and they corresponded to a donor with cystitis, who was consequently excluded from the study. The rest of the samples had mutational profiles similar to that of the pooled cohort. Moreover, we expect that the presence of artifactual mutations across samples in the cohort would result in the extraction of a mutational signature representing them. As discussed in Supplementary Note 5, this is not the case.

Comparison of ultradeep sequencing with other technology

To have an orthogonal comparison of the error rate we can compare the mutation pattern and mutation density of our study with that of normal bladder urothelium from autopsies obtained through laser capture microdissection of clonal or quasi-clonal structures[18]. In this case the sequencing approach is bulk whole-genome sequencing, which allows the comparison of duplex and bulk genome sequencing in the same tissue and type of samples (that is, normal bladder urothelium).

This comparison revealed strikingly similar mutational profiles (Extended Data Fig. 2b), suggesting the absence of a large number of artifacts with a different profile across the

mutations identified in the duplex sequencing of brushes of normal urothelium. In addition, the mutation density of the normal urothelium in our study revealed a very close match with the values obtained in the laser microdissection study (Extended Data Fig. 2c), again pointing to a low proportion of errors in our study. Moreover, the distribution of mutation density across both cohorts is very similar, as is the linear relationship between mutation density and age, which would be incompatible with high error rates across the samples in our cohort.

Conclusions

The calculation of the potential rate of errors inherent to the DNA duplex sequencing technology employed in this study shows that it is two orders of magnitude lower than the mutation density observed across the studied cohort, ensuring that the proportion of errors is in general low. Several orthogonal analyses presented above strongly suggest the lack of appreciable contribution of artifacts to the mutations observed across samples.

Supplementary Note 5: Identification of mutational signatures

Abstract

This Supplementary Note describes the identification of the mutational signatures active in this cohort using two independent algorithms. We demonstrate reliable extraction of signatures with high cosine similarity across both methods, high correlation between dome and trigone samples, and high similarity with signatures previously reported in normal bladder. These results show that duplex sequencing in urothelial brushes uncovers bona fide somatic mutations. In addition, these findings demonstrate that mutational signatures can be reliably identified across samples despite the panel being overrepresented with genic regions with pervasive positive selection. We pursue the etiology of each signature through their decomposition into the COSMIC reference catalog[21–23], and their comparison with different phenotypic or clinical features of the individuals included in the study.

Associations of non-protein affecting mutation density

We observed a significant association between the density of non-protein affecting mutations (i.e., those that do not change the sequence of the encoded protein) and the age of the donors (Supplementary Fig. 25 left; univariate linear regression p-value=3.2×10^{-3}) and their exposure to cytotoxic chemotherapy/radiotherapy (Supplementary Fig. 25, center; univariate linear regression p-value=1.0×10^{-5}; multivariate regression analysis including age, sex and sample location p-value=7.2×10^{-6}). No significantly higher mutation density was observed in individuals with history of smoking (Supplementary Fig. 25, right; univariate linear regression p-value=0.05) (See also Supplementary Note 9 and Supplementary Table 7). After correction for age, sex and sample location, the association remained non-significant (p-value=0.2).

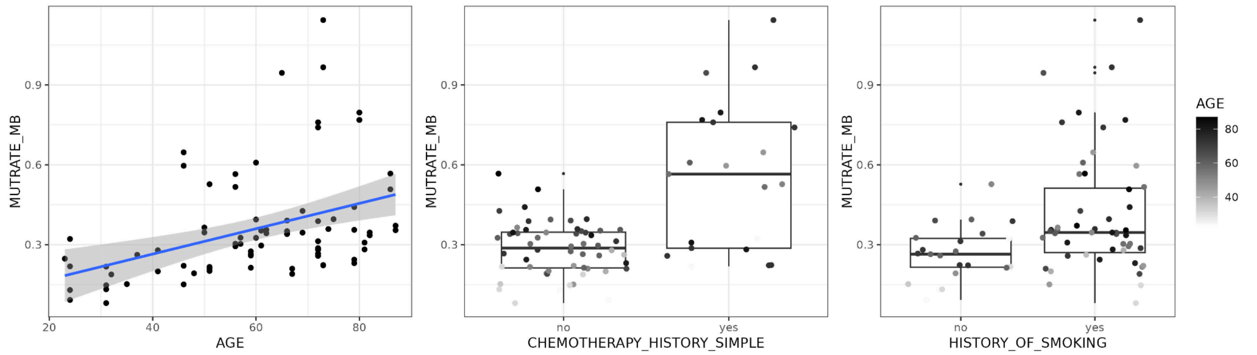

Supplementary Figure 25. Association between the density of non-protein affecting mutations and clinical features of the donors.

a) Association with the age of donors, presented as a linear regression (univariate linear regression p-value=3.2×10^{-3}).

b) Association with the exposure of donors to chemotherapy, presented as box plots showing the distribution of the mutation density in donors with and without chemotherapy history.

c) Association with the history of smoking, presented as box plots showing the distribution of the mutation density in donors with and without smoking history.

Mutational signatures identified in the cohort

We carried out a *de novo* mutational signature extraction from the mutational profiles observed across samples in the cohort. To this end, we used two different methods: a Bayesian hierarchical Dirichlet process (HDP)[24,25] and a nonnegative matrix factorization-based algorithm (SigProfiler)[22,23,26,27]. While the extraction using SigProfiler yielded three signatures (SBS96A-C; Extended Data Fig. 3a), we obtained five signatures using HDP (N1-N5; Extended Data Fig. 3b).

HDP signature N1 showed a very similar profile to SBS96A (cosine similarity 0.93; Supplementary Fig. 26), but showed also some resemblance to SBS96B. As explained in the main manuscript, these signatures summarize the APOBEC mutational process (SBS96A) and aging-related mutagenesis (SBS96B) (Fig. 1c and Extended Data Fig. 3a-d). Apparently, N1, and to some extent N3, capture these two processes together. SBS96C, which we associated with chemo/radiotherapy-related mutagenesis, is very similar to HDP signature N2. The remaining N4 and N5 show very little activity across the cohort (less than 7% each), with the exception of a few samples (Extended Data Fig. 3a-d).

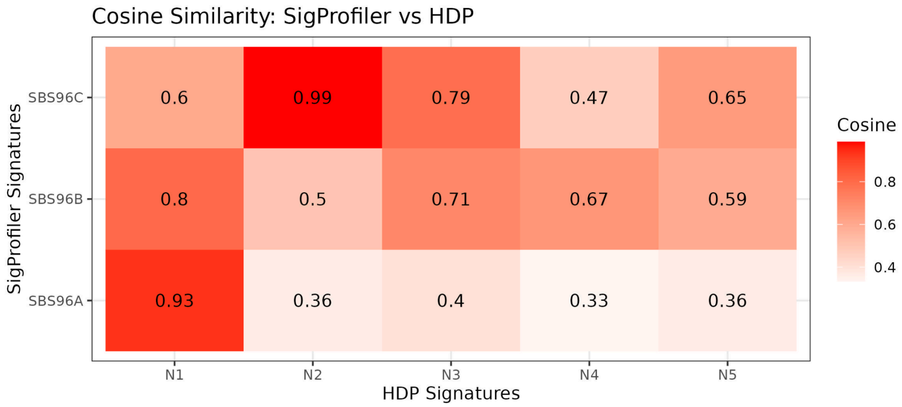

Supplementary Figure 26. Comparison of de novo signatures extracted by two independent methods.
Values in the heatmap represent the cosine similarity of each pair of signatures.

Decomposition of extracted signatures on the reference catalog

Formally, SBS96A (N1, according to HDP) can be represented as a linear combination of COSMIC signatures SBS2 (23.4%), SBS13 (19.7%) and SBS40a (56.9%) with a cosine similarity 0.93 (Supplementary Fig. 27, left). Therefore, we named it SBS-APOBEC (as the etiology of SBS40a is unknown). APOBEC mutagenesis and its associated signatures have already been described in normal bladder urothelium[18], where it was shown to be specific to some clones, as well as in bladder tumors[18,23]. In our data it was found across almost all samples, probably due to the wide brushing collection that leads to the combined analysis of a

mixture of clones (see main manuscript), although its contribution varies between individuals (Extended Data Fig. 3b,d).

The second most active signature in the cohort, SBS96B, is very similar to SBS5 (cosine similarity=0.91). Formally, SBS96B can be represented as a linear combination of COSMIC signatures SBS5 (92%) and SBS97 (8%) with a cosine similarity 0.94 (Supplementary Fig. 27, center). Since SBS5 is known to be clock-like[28], we checked the association of the activity of SBS96B with the age of donors. This analysis showed that its activity (measured in absolute number of mutations) strongly correlates with age (Fig. 1d in the main manuscript). We thus refer to SBS96B as SBS-aging.

The third signature, SBS96C (N2) has not been previously described in normal bladder. Attempts to decompose it on the COSMIC catalog give a preponderance to SBS3, a flat signature associated with homologous recombination deficiency, which is absent from this cohort. We found no other meaningful decomposition of SBS96C (Supplementary Fig. 27, right). However, we noticed a strong association of this signature with prior exposure to chemotherapy (Fig. 1d of the main manuscript). It is very difficult to associate this signature with a particular chemotherapy, as all exposed donors (11) received combinations of multiple agents (Supplementary Table 1).

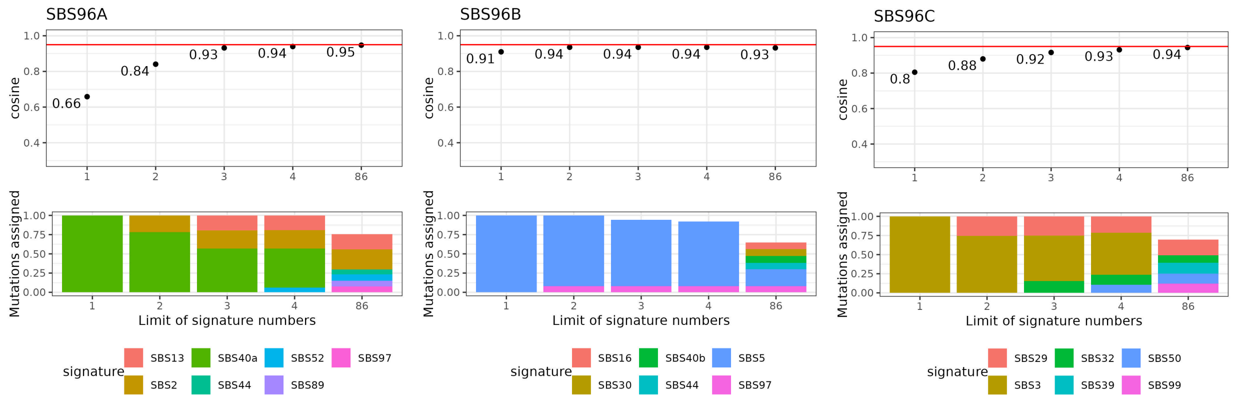

Supplementary Figure 27. Decomposition of de novo extracted signatures in linear combination of cosmic signatures using deconstructSigs.
The x-axis represents the signatures allowed for the reconstruction of the mutational profile of the cohort in each case. The top plots show the cosine similarity between the observed and reconstructed profiles. The bottom plots show the activity of each cosmic signature in the decomposition.

One salient difference between the mutational signatures active in these normal urothelium samples and those identified in a previous study also probing normal bladder urothelium[18] is the absence in the extraction carried out here of a specific smoking-related signature. The aforementioned study identified what is now called SBS92 in the COSMIC reference catalog[23], and described it as specifically associated with tobacco smoking in bladder. However, in this study SBS92 was not extracted from the observed mutational profile of the samples. The failure

to detect this signature may be related with the constraint in trinucleotide sites imposed by the panel, the relative enrichment of positive selection across observed mutations, or the limitations ascertaining smoking history from medical records. Larger genomic regions probed using duplex sequencing across more donors are likely required to discern the signature of tobacco-driven mutagenesis in normal urothelium.

Activity of mutational signatures in the dome and trigone

We also observed a good overall correlation in the activity of signatures SBS96A, SBS96B and SBS96C (as well as N1, N2, and N3) across the bladder dome and the trigone of the donors (Supplementary Fig. 28). These results indicate a similar effect of APOBEC and chemotherapy in the top and the bottom of the bladder epithelium, as expected, and validate our approach for mutation detection and signature assignment across samples.

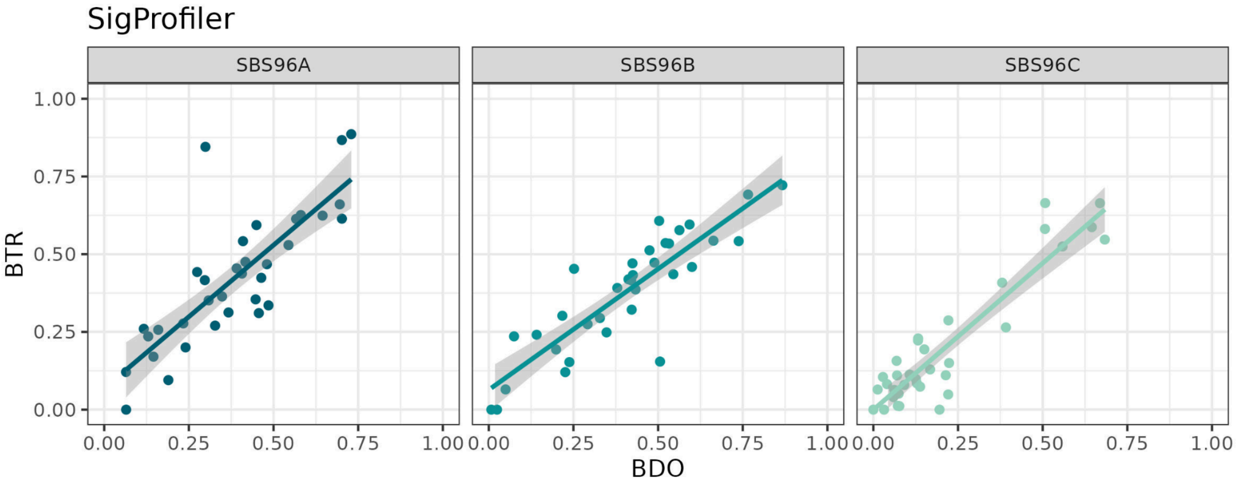

Supplementary Figure 28. Correlation of the activity of different signatures computed using SigProfiler in the dome (BDO) and the trigone (BTR) of the bladder of the same individuals.

Representativeness of observed mutations of active mutational processes

We reasoned that sequencing only a small portion of the genome, captured by the panel of 15 genes (and TERT promoter), may affect how well the mutations observed in the mixture of clones represent the mutational processes active across normal urothelium in at least two ways. First, the distribution of trinucleotide sites in the panel does not match their observed distribution across the entire genome, with an abnormal enrichment for genic sites (Supplementary Fig. 29). While the difference in distribution can be corrected by rescaling the frequency of sites in the panel to that in the genome, still a bias against sites that appear frequently mutated by a given process may result in hindering its detection. Secondly, in a scenario of pervasive positive selection (see corresponding section in the main paper), the enrichment for genic mutations

(more likely to be under selection) may result in a profile that is biased away from the mutational processes active in purely neutral mutagenesis.

The trinucleotide composition of the panel differs from those of the whole genome (Supplementary Fig. 30) and also is affected by difference in the sequencing depth of different positions.

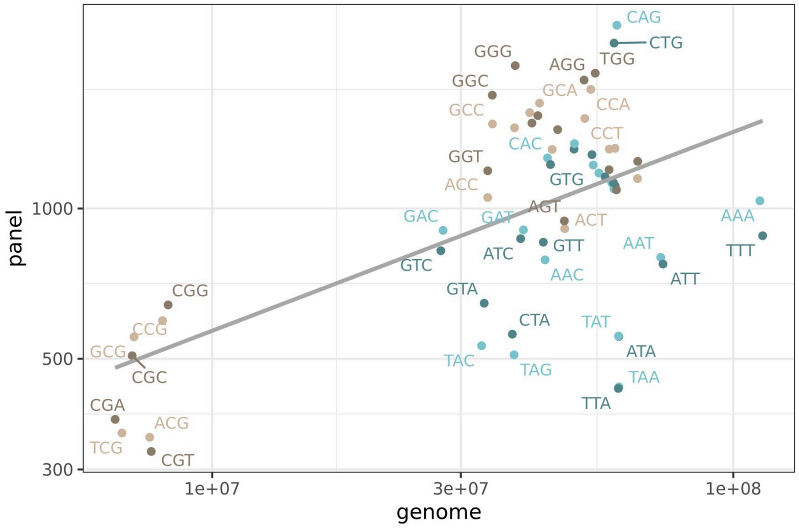

Supplementary Figure 29. Abundance of different trinucleotides among the sites captured in the panel and the whole genome.
Trinucleotides are colored depending on the nucleotide in the middle position. The grey line represents the linear correlation of the trinucleotide frequencies in the panel and in the whole genome.

We also observed that the mutational profiles obtained from all or only non-protein affecting mutations captured by the panel, are very similar to that obtained from whole-genome mutations from laser capture microdissection (LCM) of the normal bladder urothelium[18] (cosine similarities, 0.91 and 0.95, respectively; Supplementary Fig. 30). This strongly suggests that there is not a strong bias in observed mutation contexts.

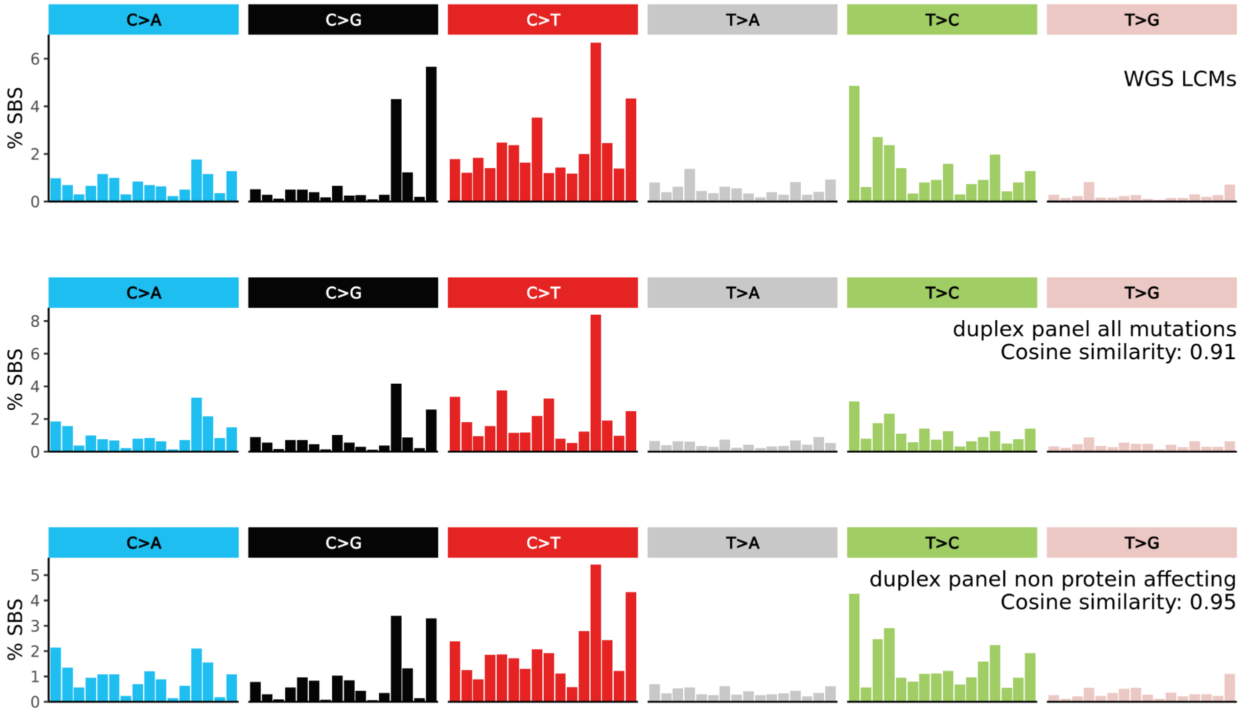

Supplementary Figure 30. Comparison of mutational profiles in normal urothelium.
The top row presents the profile of mutations obtained through whole-genome sequencing of LCMs of normal urothelium. The middle row presents the profile of mutations (both protein- and non-protein affecting) observed in 16 genes across all samples probed in this study using duplex DNA sequencing. The bottom row presents the profile of non-protein affecting mutations observed across all samples using the study panel. For the middle and bottom rows, the cosine similarity to the profile in the top row is shown.

To explore the problem of mutational profiles biased by positive selection, we directly compared, for each sample, the mutation density and mutational profile computed using all mutations with those obtained using only non-protein affecting mutations. For this analysis, we computed, for each sample, the excess of protein-affecting mutations (dN/dS; see section on calculation of positive selection, below) with respect to the expectation under neutrality as a measure of the overall magnitude of positive selection acting on the mutations of the 15 genes. Despite the existence of a strong positive correlation between the density of protein affecting mutations and all mutations across samples (Supplementary Fig. 31a), the higher the excess of mutations over the neutral expectation (higher dN/dS), the higher the degradation of the cosine similarity of their non-protein affecting-to-all mutational profile (Supplementary Fig. 31b). This is expected, as higher dN/dS values indicate a preponderance of protein affecting mutations. This may impact the identification of mutational signatures active in these samples. Nevertheless, the aforementioned comparison between the non-protein affecting mutations captured by the panel and that obtained from whole-genome mutations from LCM suggests that this deviation is not large enough to cause a great distortion in the profile.

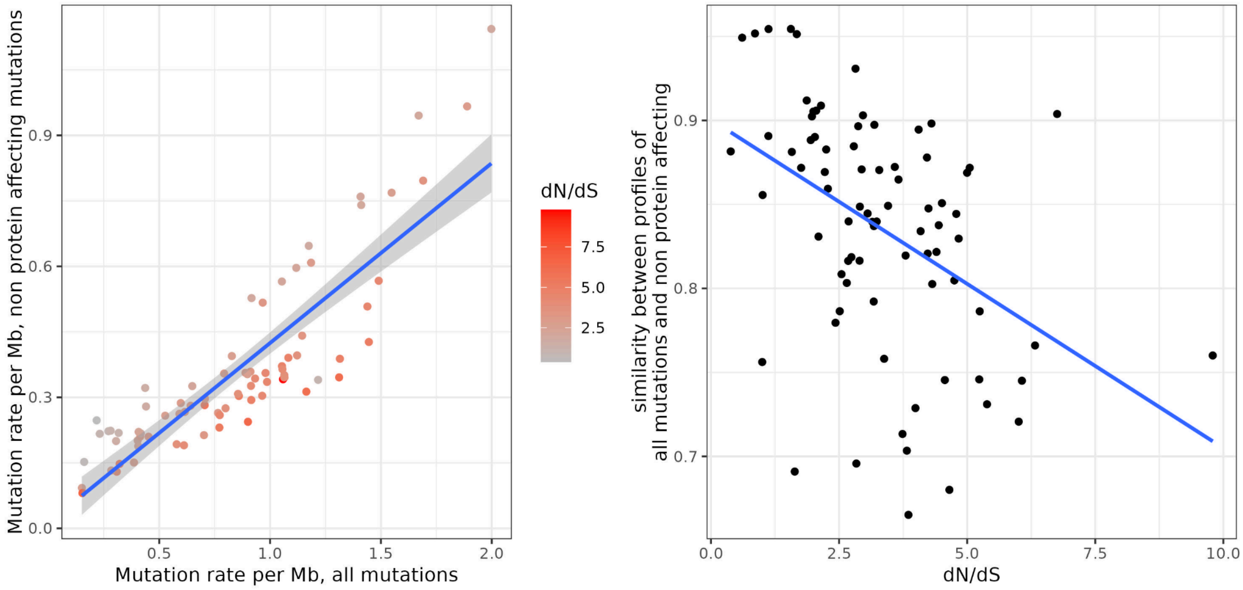

Supplementary Figure 31. Effect of pervasive positive selection on the mutational profile of normal urothelium samples.

a) Correlation of the mutation density observed for all sites and only for non-protein affecting sites across the genes in the panel. The dots represent samples, and they are colored following the excess of protein-affecting mutations observed in each of them (dN/dS). b) Relationship between the similarity of the mutational profile computed for all mutations and only protein-affecting mutations (cosine similarity, y-axis) and the excess of protein-affecting mutations observed in each sample (dN/dS, x-axis). The downward trend implies that the higher the magnitude of positive selection observed in the sample the more dissimilar (lower cosine similarity) the profile of protein-affecting mutations and that of all mutations. Both plots show that a high magnitude of positive selection may cause a distortion in the profile of mutations observed in a sample with respect to that expected under purely neutral mutagenesis.

We reasoned that *de novo* extraction of mutational signatures using only non-protein affecting mutations would be the best way to minimize the effect of selection on the observed mutational profile. However, this substantially reduces the number of mutations available for the calculation of mutational signatures. Both SigProfiler and HDP yielded 2 signatures as the most robust solution of the extraction: one of these was similar to SBS-treatment and the second one appeared to be a combination of SBS-age and SBS-APOBEC. This similarity of the mutational signatures extracted using all mutations and only non-protein affecting mutations suggests that the two aforementioned factors do not introduce an important bias in the mutational profile of samples.

In summary, the difference in the set of mutational signatures (most significantly the absence of smoking signature) extracted from a mixture of clones in samples of this cohort and those extracted from whole-genome bladder LCMs does not appear to emerge from the overrepresentation of genic sites that distorts the distribution of available trinucleotide sites and the pervasive positive selection in the samples of normal urothelium analyzed.

Supplementary Note 6: Positive selection

Abstract

As described in the main manuscript, the calculation of the magnitude of positive selection on mutations in different genes and subgenic regions is a cornerstone of this study. Here we describe four methods to probe and quantify positive selection across genomic elements and samples based on SNVs (OncodriveFML, Oncodrive3D, Omega) and one additional method based on indels (frameshift enrichment). Two of these methods (OncodriveFML[29], Oncodrive3D[30]) had been previously developed to calculate positive selection on tumor somatic mutations and have been adapted to work on ultradeep sequencing data by accounting for differences in sequencing coverage at different genomic positions in a gene and between different samples (Supplementary Note 3). These adaptations are described below. Omega (https://github.com/bbglab/omega) and frameshift enrichment (available in deepCSA) have been developed specifically for this study, and their design and implementation are described below.

Consequence types conventions

We used ENSEMBL VEP v.111[31,32] to map the mutations observed in samples to protein coordinates and obtain their consequence types. To this end we use the set of MANE Select transcripts defined by GENCODE and REFSEQ[33]. SNVs affecting the coding sequence (or occurring directly at intronic splice sites), with consequence type missense, nonsense or essential splice (either exonic or intronic) are deemed protein affecting and annotated as such. In all analyses shown in the main manuscript, nonsense and essential splice SNVs are lumped together in a category called truncating. All other SNVs mapping to introns and synonymous SNVs are deemed non-protein affecting. Indels overlapping the coding sequence of the genes are classified into frameshift and inframe. Those falling into the non-coding regions of genes are all classified as non-protein affecting, although we record whether their length is multiple of 3 nucleotides or not to carry out the frameshift enrichment test.

Background mutation density

All three methods to compute the positive selection on SNVs make use of site-specific background mutation density. That is, they are based on modeling the expected mutation density under neutral evolution across genomic sites. Our approach to model the background mutation density for these three methods has three key features. First, it is based on the observed frequency of tri-nucleotide changes across all genomic positions sequenced; second, a model is built for each gene; and third, it accounts for the variability in sequencing depth across genomic sites and samples (Supplementary Note 3).

This last aspect is unique for ultradeep duplex sequencing of a mixture of clones in a normal tissue. When the same methods are applied to somatic mutations in tumors or to mutations obtained from the sequencing of clonal or quasi-clonal structures in normal tissues, the observed mutation density can be considered an unbiased reflection of the underlying (biological) probability of a given substitution to occur in a given tri-nucleotide context. In these cases, the potential variability of the sequencing coverage is small and has no consequences

on the calculation of positive selection, as all mutations identified are assumed to have occurred in one cell and grow through clonal expansion. However, when analyzing somatic mutations that occur at very low frequency using ultradeep duplex sequencing, the sequencing depth/coverage at each position has a significant impact on the probability to observe a mutation at a particular site. Thus, these different probabilities arising from variable sequencing coverage need to be accounted for when modeling the background mutation density.

Relative mutation density per tri-nucleotide context

Given a sample and a genomic region of interest we can estimate the relative mutation density across trinucleotide contexts. Given the substitution type R>a, where "R" represents the reference trinucleotide (one out of the 32 pyrimidine-centric trinucleotides) and "a" the alternate allele, let n(R>a) denote the number of sites in the region where the R>a change has been observed, and let N(R) denote the number of duplex reads mapping to sites with reference tri-nucleotide R, which can be calculated as the sum of site-specific depths D(x) across all positions x with reference tri-nucleotide R,

$$N(R) = \sum_{x \in R} D(x).$$

The mutation density of the substitution type R>a per sequenced read is given as:

$$\tilde{r}(R > a) = \frac{n(R>a)}{N(R)}$$

We define a vector r, indexed by the 96 possible single-base substitution trinucleotide context, as follows:

$$r(R > a) = \frac{\tilde{r}(R>a)}{\sum_{R>a} \tilde{r}(R>a)} \quad (1)$$

In particular, the entries of r represent relative densities per trinucleotide context that correct for the possibly uneven sequencing coverage across trinucleotides.

Synonymous mutation density

Neutral mutagenesis is heterogeneous across genomic elements and regions and this has been explained by a number of genomic features such as DNA sequence, chromatin conformation, the transcriptional status of genic regions or the replication timing[34–36]. In order to calibrate the relative mutation density per trinucleotide context into a per-site mutation density model, we are required to estimate a baseline neutral mutation density per gene. Methods such as dNdScv[37] propose a regression approach using maps of epigenetic modifications, replication time and other genomic features as covariates. The observed mutation density of genes in whole-exome or whole-genome sequencing experiments is used to regress out an accurate baseline neutral mutation density. With a small panel of genes this approach is not feasible, so we need to resort directly to the synonymous mutation density per gene to infer the baseline neutral mutagenesis. The validity of the observed synonymous mutation density of the cohort to estimate the neutral mutation rate is guaranteed by the high sequencing depth achieved.

Given a sample, we would like to use the synonymous mutation count per gene as a reference of the neutral mutation density at that gene-sample. Because mutation calling can yield sparse

synonymous mutation counts per gene-sample, we must resort to simplifying assumptions. After verifying that the distribution of synonymous mutation density across genes is concordant across samples, we proceed to compute per-gene-sample synonymous mutation density by considering the relative distribution of synonymous mutation density per gene pooling the entire cohort, then redistributing in each sample the number of synonymous observed across all genes according to the proportions cohort wise. Specifically, if S_g is the cohort-wise, depth-adjusted synonymous mutation density at gene g and if sample s has a total synonymous burden of N_s, then for sample s and gene g we compute the synonymous mutation density as follows:

$$\mu_{sg} = D_{sg} \cdot S_g / \sum_k S_k$$

$$Syn(s, g) = N_s \cdot \mu_{sg} / \sum_k \mu_{sk} \quad (2)$$

where D_{sg} is the total depth across synonymous sites in sample s and gene g, and *k* represents the total number of genes.

Gene and site specific mutation density

Given a sample and a gene we now describe a method to derive site specific mutation density under neutral evolution. Let r be the vector of relative density per trinucleotide context of the sample (equation 1) and let S be the synonymous mutation count estimate in that sample-gene (equation 2). Given the set of sites that yield a synonymous mutation in the gene, their trinucleotide context and the sequencing depth at each of these sites, we can estimate the scaling factor α such that $\alpha \cdot r$ is the mutation density per site per read that best explains the synonymous mutation count. Let N(R>a) be the number of reads mapping to R>a sites that encode a synonymous mutation. The value of α can be inferred from the following equation:

$$S = \sum_{R>a} \alpha r_{R>a} N_{R>a}$$

The mutation density of the R>a substitution at any mutation site x of the gene with trinucleotide context R>a will be given by the expression:

$$\lambda_x = \alpha r_{R>a} D(x).$$

This approach easily generalizes when considering groups of samples by adding the mutation density per site across samples.

Mutation probability vectors

Some methods require the specification of a probability vector (normalized to sum 1) indexed by all the possible mutation sites in a given region. Once the mutation density $\lambda(x)$ are computed per sample, they can be added per site if necessary and simply divide each by the total sum across all sites.

OncodriveFML

OncodriveFML[29] is a driver discovery method devised for measuring selection in the context of tumor evolution that we have repurposed for the analysis of clonal selection in healthy tissue.

The method tests the distributional difference between the expected mean functional impact and the observed one (functional impact bias) on a gene by gene basis based on precomputed functional impacts (CADD score[38]). The method relies on the redistribution of as many mutations as they have been observed in a gene according to an input mutation probability vector, defining a probability for each site in the gene to undergo a mutation neutrally. The method has been adapted to work with custom mutation probability vectors whereby the depth correction has already been incorporated (see "Background mutation density" section above).

The method computes a Z-score and a p-value for each gene tested. The Z-score takes advantage of the fact that we can compute exactly the expected mean functional impact and the standard deviation from the mutation probability vector and the corresponding vector of precomputed functional impacts (CADD score):

$$\mu = \sum_x p_x I_x, \quad \sigma^2 = \sum_x p_x (I_x - \mu)^2, \quad z = \frac{\sqrt{n}(\bar{I} - \mu)}{\sigma}$$

where $\bar{I}$ is the mean observed functional impact across n mutations, where x runs through all the mutation sites of the gene and p(x) represents the mutation probability of x.

Oncodrive3D

Oncodrive3D[30] is a driver discovery method devised for measuring selection in the context of tumor evolution that we have repurposed for the analysis of clonal selection in healthy tissue. The method tests for mutational clustering anomalies taking into consideration the mapping of the mutations onto the 3D protein conformation obtained from the AlphaFold database[39,40]. For each residue, the method defines an enrichment score that accounts for the density of missense mutations mapping to residues located within a spherical volume around the residue. Then, for several iterations the method randomizes mutations based on the relative mutation probability of each site and computes the resulting enrichment scores. Ranking the observed and the randomized enrichment scores and comparing the observed against the distribution of randomized scores, the method derives a significance which is then used to identify 3D clusters of missense mutations.

The difference between the observed and expected enrichment scores of the most significant volume, along with its corresponding p-value, are assigned as the gene's score and p-value, respectively. Both the enrichment scores and the randomization procedure require mutation probability vectors. We redefined the method accordingly so that we can feed it on mutation probability vectors that account for the uneven sequencing depth across samples and genomic positions (see "Background mutation density" section above).

Omega

Omega is a driver analysis method owing its formulation to the dNdScv method[37], which can be considered an adaptation of, albeit with some differences from the implementation point of view, such as the correction for uneven sequencing depth and the model specification. Briefly, the method models mutation counts as following a negative binomial distribution with mean depending on the background mutation density per site and a correcting factor (dN/dS) intended

to model the excess of observed mutations over expected uniformly for all the trinucleotide contexts. Given a catalog of observed mutations in a genomic region of interest across a group of samples, omega estimates a dN/dS with confidence bounds and provides a p-value.

Let $G = \{g_1, ..., g_k\}$ be a group of samples and $X = \{x_1, ..., x_m\}$ be a collection of mutation sites with a specified non-synonymous consequence type of choice. By default our implementation sets several consequence types of interest derived from Sequence Ontology[41] terms:

"missense": ["missense"],
"nonsense": ["nonsense"],
"essential_splice": ["essential_splice"],
"truncating": ["nonsense", "essential_splice"],
"nonsynonymous_splice": ["missense", "nonsense", "essential_splice"]

Using the background mutation density $\lambda(g, x)$ specified for each sample g and mutation site x (see “Background mutation density” section above) we model the count of mutations n(R>a) observed at a given context R>a as:

$$n_{R>a} \sim NegBinom(\omega\mu_{R>a}, \theta) \text{ with } \mu_{R>a} = \sum_{g} \sum_{x \in X \cap \{R>a\}} \lambda(g, x)$$

Experiments with synthetic data yield similar results within a range of choices for the overdispersion parameter. For the main analysis, we set a moderate overdispersion θ=0.1, implying that the variance of the distribution is higher than the mean μ, i.e. $\sigma^2 = \mu + \theta\mu^2$.

We fit the model with a maximum likelihood approach by solving for the dN/dS parameter ω that maximizes the log-likelihood function

$$l(\omega) = \sum_{R>a} log\, L_{R>a}(\omega, n_{R>a})$$

where $L_{R>a}(\omega, n)$ is the likelihood function of the negative binomial distribution with mean $\mu_{R>a}$ and the sum runs along all the trinucleotide contexts. We determine the best fit $\hat{\omega} = argmax_w\, l(\omega)$ with Python scipy.optimize (Nelder-Mead method).

We can compute the confidence intervals and p-values associated with the best fit via the Wilks log-likelihood ratio statistic $W(\omega)$ defined as $W(\omega) = 2(l(\hat{\omega}) - l(\omega))$. The confidence interval with confidence level α can be computed as the set of values

$$CI_\alpha = \{\omega \mid W(\omega) \leq c_\alpha\}$$

where c_α is the $(1 - \alpha)$ quantile of χ^2_1 the chi-square distribution with one degree of freedom. Using the fact that $W(\omega) \sim \chi^2_1$ we can as well compute a one-sided p-value associated with $\hat{\omega}$ as the probability mass

$$p = \int_{W(1)}^{\infty} \varphi(s)ds,$$

where φ is the probability density of the χ_1^2 distribution.

The dN/dS point estimate $\hat{\omega}$ readout can be translated into an excess of mutations over expected by the transformation $(\hat{\omega} - 1)/\hat{\omega}$.

Calibration of omega

For the sake of ensuring correct calibration of the dN/dS determination method, we conducted several experiments with synthetic data whereby sets of mutations were randomly generated subject to a prescribed baseline mutation density and dN/dS value, then applied our method to reconstruct the point estimate dN/dS and its 95% confidence intervals.

Taking as reference the distribution of depths across sites observed in our sequencing data and the inferred depth-adjusted neutral mutation density across samples, we simulated Poisson distributed counts for each 96-channel trinucleotide context in accordance with the expected trinucleotide context specific mutation density of the whole cohort of samples (“all_samples”). A ground truth dN/dS was employed to rescale the mean of the Poisson distribution. We tried several overdispersion parameters for the negative binomial based MLE inference to confirm that our choice was not detrimental in the data context of the main analysis.

Overall the coverage was close to the nominal value across the configurations tested (Supplementary Fig. 32). Although in our study a data-informed choice to infer an overdispersion was not obvious, in future studies comprising larger panels of genes it is conceivable that this parameter is informed directly from the input data.

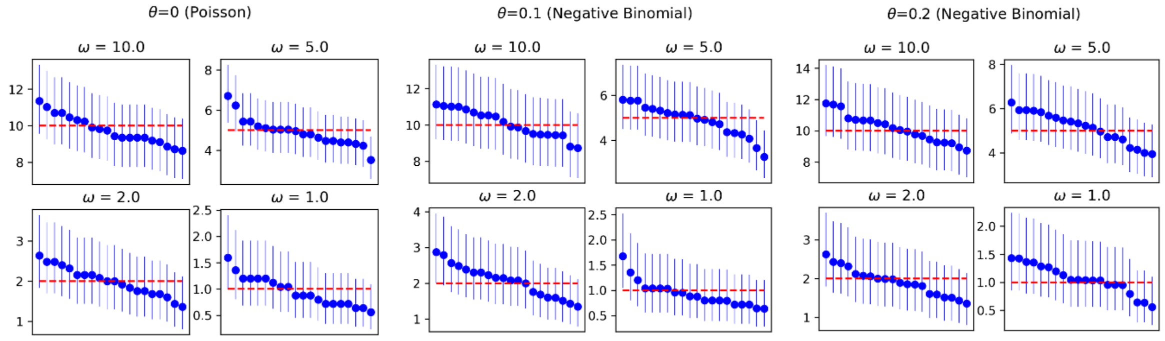

Supplementary Figure 32. Simulation tests of Omega.

Reconstruction of the ground-truth dN/dS for several values 1, 2, 5 and 10 across three different inference configurations: Poisson and negative binomial with overdispersion 0.1 and 0.2, respectively. These simulations took as a reference the distribution of depths sequenced in the gene TP53 with an exemplary trinucleotide specific mutation density, with total mutation burden matching that of the entire cohort. For the 20 random simulations represented in each case, the confidence interval coverage oscillated between 90% and 100%.

Smoothing synonymous mutation counts

The calculation of dN/dS for each gene across samples was possible using the number of observed synonymous mutations. However, to compute dN/dS values for a gene in individual samples, or for within-gene elements, such as exons or protein domains (see below), in certain cases the number of observed synonymous mutations was too sparse to make a meaningful calculation. We could not resort to estimate the number of synonymous mutations based on a regression of covariates of the mutation density (as in dNdScv[37]), due to the small number of genes included in the panel.

To solve this problem, we reasoned that the number of synonymous mutations observed across genes in any given sample could be smoothed out using the relative proportion of synonymous mutations born by each gene across the entire cohort. We could then apply these relative proportions –as surrogate of the relative levels of neutral mutation density across all genes– to the set of synonymous mutations observed in any sample. This set of mutations would then be distributed across all genes following these relative proportions. We call the value of Omega calculated using these smoothed synonymous counts, dN/dS *globalloc*, after the fusion of the words global and local. We verified that dN/dS values computed directly using the number of observed synonymous mutations and those of the dN/dS *globalloc* are very similar (Supplementary Fig. 33). Throughout the manuscript, the values of dN/dS used for different analyses at the level of samples correspond to dN/dS *globalloc*.

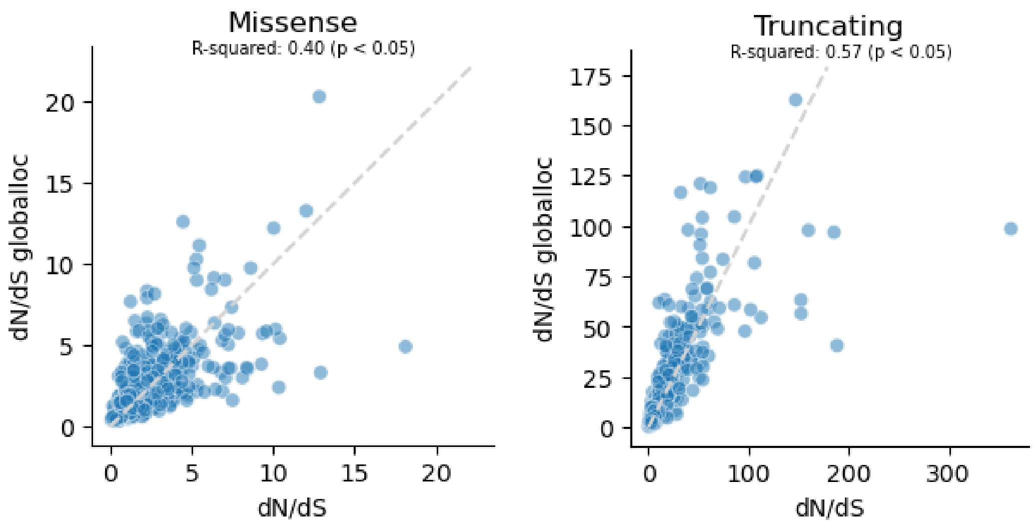

Supplementary Figure 33. Agreement between dN/dS and dN/dS globalloc. The dashed grey line represents x=y (dN/dS = $dN/dS_{globalloc}$).

Frameshift enrichment

The rationale underlying this method is that indels causing a shift of the reading frame of a gene (frameshift, i.e., indels within the coding sequence of a gene with length not exactly divisible by

3 nucleotides, or non-3n) are likely to be more deleterious than indels that do not disrupt the gene's reading frame (inframe, with length exact multiple of 3, or 3n).

To build a background model representing how the ratio of non-3n-to-3n indels is expected under neutrality (i.e, in the absence of any positive selection), we exploited the number of non-3n and 3n indels observed in intronic regions of the gene under analysis. This assumes that both intronic non-3n and 3n indels have a null (or at least comparable) effect on the expression/function of the gene. We then used a G-test[42] to analyze whether the non-3n-to-3n ratio of indels in the coding region of the gene and the corresponding non-3n-to-3n ratio of intronic indels were significantly different. We thus report the p-value yielded by the G-test and the log-fold-change to assess the relative enrichment or depletion of non-3n indels in the coding region.

Positive selection in the TERT promoter

We applied the dN/dS approach implemented within Omega to the calculation of positive selection in mutations observed in the TERT promoter. To this end, all mutations that have been observed at least twice across 8,136 WGS tumor samples sequenced by the Hartwig Medical Foundation and the PCAWG consortium[9,10,43–45] were considered activating. All other mutations observed in the TERT promoter were considered not functional. We then used the number of not functional mutations to estimate the expected number of activatingmutations in the promoter under neutrality. This allowed us to calculate a dN/dS-like value (*dN/dS* TERTp) based on the ratio of functional to non-functional mutations observed in this element across samples. With the *dN/dS* TERTp we obtained a measure of the excess of functional mutations in the promoter over the expectation under neutrality.

Positive selection in different within-gene elements

We hypothesized that the same or similar approaches to estimate positive selection could be applied to specific within-gene elements, such as exons, protein domains, or even individual amino acids. In the following subsections, we describe the calculation of missense and truncating dN/dS values for individual exons and protein domains, as well as the estimation of the strength of selection at individual sites within the protein.

Exon and domain-wise dN/dS

All exons or protein domains of the genes included in the panel (based on the Pfam definition of domains) were converted into new genomic elements bearing the number of non-synonymous mutations observed within their boundaries. The values of *dN/dS missense* and *dN/dS truncating* were then calculated for all these new genomic elements using the same Omega implementation as for full genes. We used the *globalloc* implementation (see above) to estimate the number of synonymous mutations to compute these dN/dS values. The number of synonymous mutations expected in a specific exon or protein domain was obtained by multiplying the number of synonymous mutations expected in the gene as described above, by the proportion of mutation probability corresponding to the sub-genic region of interest. Thus we

assume that the rate at which mutations occur within a gene does not change except for the differences in trinucleotide mutation probability.

Site selection

To estimate the strength of selection at the level of individual mutation or amino acid change at a given position or protein position, we resorted to an *ad hoc* method inspired on the ideas of dN/dS. We can compute the probability to observe any particular mutation in a gene using the tri-nucleotide change frequencies, the sequencing depth at the corresponding genomic position and the expected mutation density based on the estimations described above (see sections on Synonymous mutation density and Smoothing synonymous mutation counts). The site selection at the level of the genomic mutation is then calculated as the ratio between the number of times the mutation has been observed across samples and its probability to occur. The calculation at the level of residue change was carried out as the ratio between the number of observed mutations with all nucleotide changes resulting in the same amino acid change and their sum of probabilities. Analogously, to compute the site selection at the level of amino acid residue, we used the ratio between the sum of all possible mutations in the codon in question and the sum of probabilities of the nine possible nucleotide changes.

To define the p-value of each comparison, we used a Poisson distribution with the number of expected mutations as mean and from it, we calculated the probability of obtaining the observed number of mutations. We set the significance threshold at 10^{-5} to account for the number of tests carried out.

Positive selection in dome and trigone of the same donors

The dome (upper part) and trigone (bottom portion) of the bladder are two structurally and functionally distinct regions within the organ. Our study design included sampling ~2cm^2 of the dome and trigone urothelium of each individual to answer whether the clonal evolution in the urothelium differs between both regions. The comparison of the clonal landscape revealed by ultradeep sequencing showed that the two areas sampled from each individual are highly similar: the genes with the highest clonal selection in the dome also exhibited the highest clonal selection in the trigone (Extended Data Fig. 5c). In addition, we observed a high proportion of shared mutations between the dome and trigone of the same individual (Extended Data Fig. 6a,b). These results could be partly explained by convergent evolution, that is, the independent selection of the same mutation in two different clones due to shared exposures and selective pressures. However, a fraction of the concordant mutations between dome and trigone included non-protein affecting variants, indicating that mechanisms other than convergent evolution must be in place. One possibility is intraluminal seeding, which has been previously proposed as an explanation for multifocal bladder tumors[46]. While epithelial brushes were carefully performed, the possibility of cross-contamination cannot be ruled out. Remarkably, however, positive selection per gene between dome and trigone remained similar even when accounting only for non-shared mutations (Extended Data Fig. 6c).

This overall high similarity indicates that, despite the differences in exposure to urine of the dome and the trigone, the evolutionary dynamics and selective forces in their urothelium are equivalent. Thus, a brushing of cells from ~2cm^2 of the urothelium provides a good representation of the clonal structure of the entire tissue. This allows us to use the mixture of clones probed through ultradeep DNA duplex sequencing to analyze the clonal structure of the normal urothelium of the 45 individuals in this cohort (see main manuscript).

Supplementary Note 7: Effect of sequencing depth on the calculation of positive selection

Rationale

One of the strengths of the experimental design of this study is the ultradeep sequencing of the samples, which allows the robust calculation of dN/dS values per sample for all genes under study. These, in turn, ensured that robust regressions could be carried out to probe a sex bias of the clonal landscape of the normal urothelium. It also opened up the path towards natural saturation mutagenesis.

We reasoned that this study was only possible with this experimental design, based on ultradeep sequencing. To prove this postulate, we designed a sub-sampling experiment, in which we systematically reduced the sequencing depth –that is, the effective number of clones observed– and tracked the change in the values of dN/dS obtained at each sub-sequencing step. The expectation was that the reduction of the sequencing depth would eventually make it impossible to estimate the dN/dS for an increasing number of samples (due to the absence of synonymous, missense and truncating mutations in a gene). The growth of missing dN/dS values would, in turn, reduce the statistical power of the regressions, eventually rendering them impossible to carry out.

Design

Starting with the 79 samples of the cohort, we created copies of these samples as if they had been probed with lower sequencing depth, downsampling the sequencing depth at each genomic position in each sample. The downsampling range starts from 80% of the obtained depth down to 5%, with intermediate points at 60, 40, 20 and 10%. They each represent the sequencing depth remaining in each sample after the sub-sampling, and thus, we call them the "**remaining proportion (RP)**" of sequencing depth. For every RP we created 10 replicates of the entire cohort.

In each replicate sample, the depth per position (D_i') is computed as:

$$D_i' = D_i * RP$$

Thus all randomizations of the same RP have exactly the same D_i' at a given position.

To define the mutations observed in the downsampled copy of each original sample, we computed the initial reference (REF_DEPTH_i) and alternate depths (ALT_DEPTH_i) :

$$REF\_DEPTH_i = D_i - ALT\_DEPTH_i$$

We then sampled a random subset of size D_i' from a pool containing REF_DEPTH_i reference alleles and ALT_DEPTH_i alternate alleles.

The result of this sampling provided the value of ALT_DEPTH_i' and whenever this updated value was bigger than 1 we kept the mutation with its corresponding D_i' and ALT_DEPTH_i'. With this

random sampling procedure, at each of the replicates of the same RP, the exact number and identity of the mutations changed. Supplementary Figure 34 presents the value of depth, and the average number of protein affecting and synonymous mutations per gene for each remaining proportion.

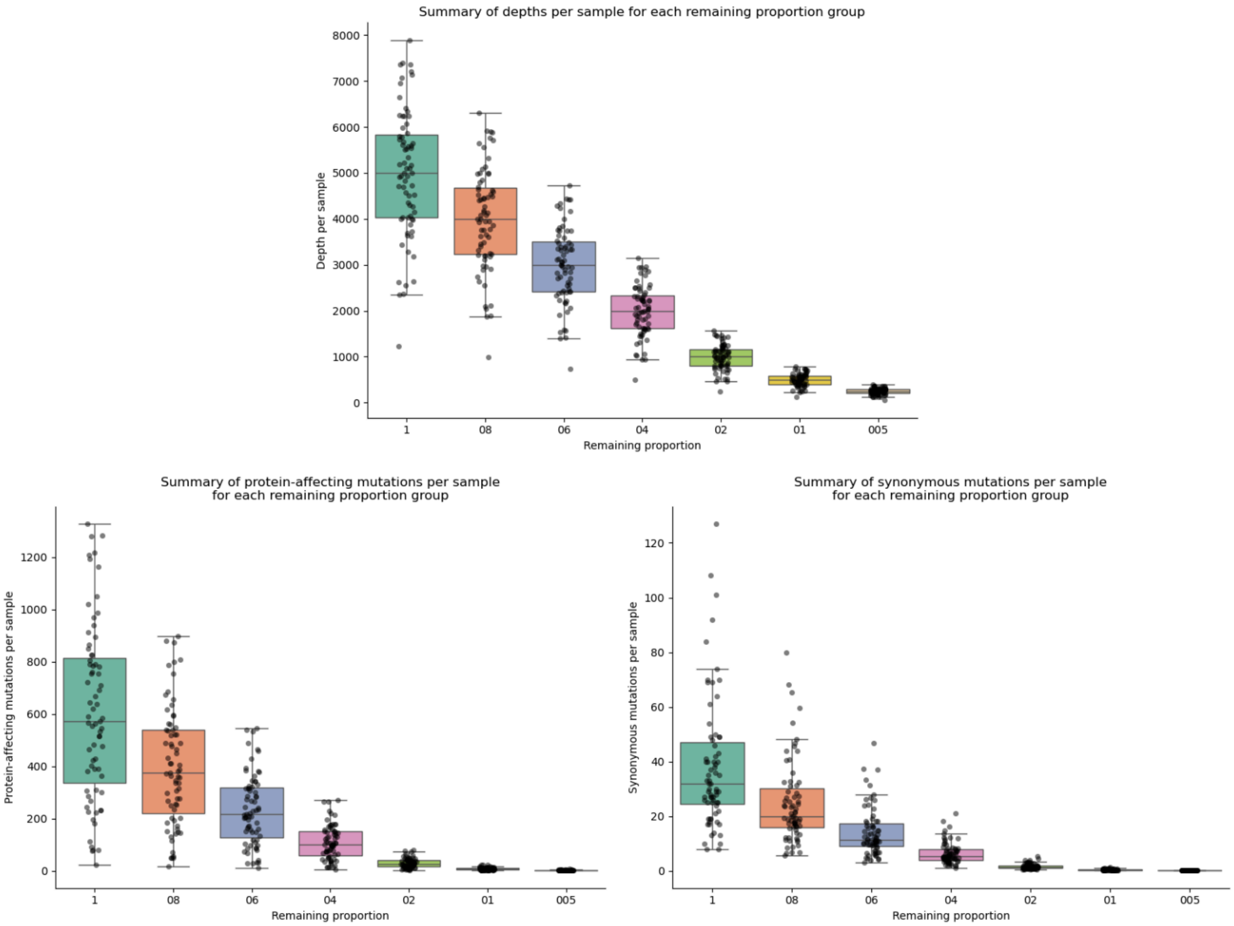

Supplementary Figure 34. Summary of average depths, number of protein-affecting and synonymous mutations per sample, for each of the different downsampling proportions.
a) The boxplots represent the distribution of values of sequencing depth remaining across samples after each down-sampling step.
b) The boxplots represent the distribution of the number of protein affecting (truncating or missense) mutations remaining across samples after each down-sampling step.
c) The boxplots represent the distribution of the number of synonymous mutations remaining across samples after each down-sampling step.
The x-axis of the plots represent the fraction or proportion of sequencing depth of the original samples that remains after each down-sampling step.

For every sample of each RP, we computed the dN/dS with both omega and omega globalloc approaches (see above) for each gene. When omega was unable to compute the dN/dS (i.e., due to absence of mutations with the given consequence in the gene in the sample), a NA value was recorded.

Results

For every sample in every down-sampling step (i.e., for different proportions of remaining depth), we made a try to calculate *dN/dS truncating* and *dN/dS* missense for every gene. The maximum possible number of dN/dS values that can be calculated in a sample are 31 (2 for each of the 15 genes + pTERT omega). This is only possible if truncating and missense mutations (besides synonyms mutations to estimate neutrality) are observed in the genes. The absence of synonymous mutations in a gene can be solved (if synonymous mutations are observed in other genes in the same sample) through the Omega globalloc approach (Supplementary Note 6). Nevertheless, the absence of truncating or missense mutations in a gene in a sample renders Omega incapable of calculating the corresponding *dN/dS truncating* and *dN/dS missense*. We observed (Supplementary Fig. 35) that as the remaining sequencing depth decreases with every down-sampling step (from 100% to 5% of the observed depth, that is, from ~5,000x to ~250x), the number of dN/dS values is proportionally reduced. In the extreme case of average depth ~250x, the dN/dS value for almost no sample/gene can be calculated.

Being able to calculate the dN/dS value for a gene in every sample is key for the success of the regression-based approach taken in this study. As the number of samples with missing dN/dS values increases, the power of the cohort to calculate the association between sex and the clonal landscape effectively decreases. An alternative approach, such as pooling all mutations observed across males and females would be required in the case that dN/dS values cannot be computed for every sample. While such an approach could suggest which genes show differences in the magnitude of positive selection between males and females, the influence of relevant covariates is not easy to take into account.

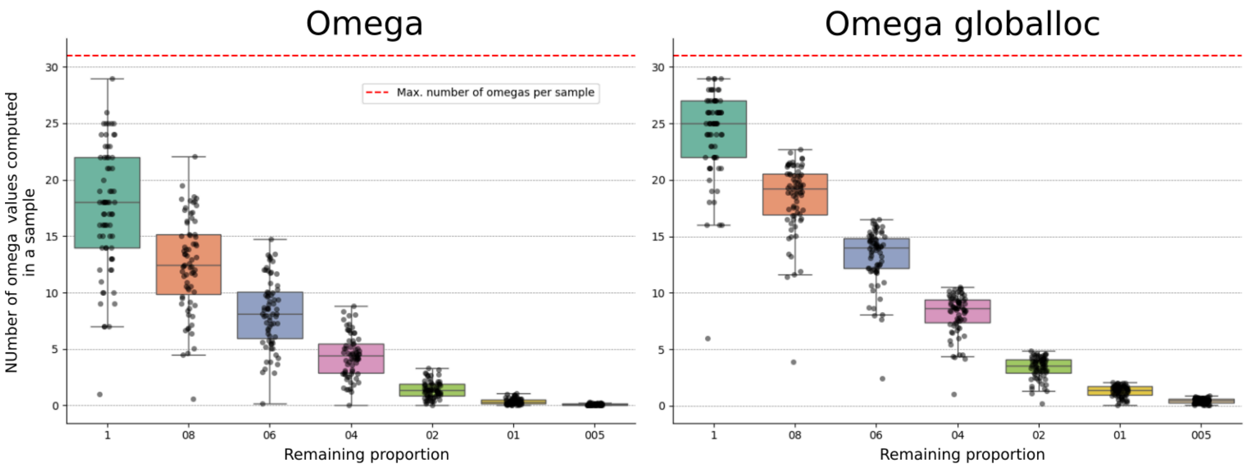

Supplementary Figure 35. Summary of the results of the down-sampling experiment.
In both panels, the boxplots represent the number of dN/dS values (*dN/dS truncating* and *dN/dS missense* per gene) that can be computed across samples after every down-sampling step using the Omega or Omega globalloc (Supplementary Note 6). The dashed red line corresponds to the case where all possible dN/dS values (31) can be computed for all genes in a sample. The plots indicate that, as the depth of sequencing is reduced, the decrease in the number of synonymous, truncating and missense

mutations makes it impossible to compute dN/dS for an increasing number of samples. These samples will be given missing dN/dS values, thus reducing the power of across-samples regressions on sex with decreasing sequencing depth.
All numbers of dN/dS values available per sample are averages over 10 replicates, except for *RP=1* that represents our cohort.

Conclusions

The depth of sequencing of the study (~5,000x per sample) is essential to be able to robustly estimate the clonal landscape of each sample, and to be able to observe their sex bias. Sequencing at lower depths (for example, 10%-20%, that is 500x-1,000x) would produce an intolerably high number of missing dN/dS values, effectively reducing the statistical power of the cohort to detect this bias. Even if sequencing at 2,000x we would only be able to compute less than half of the possible dN/dS values. An alternative study design, with a large number of individuals with samples sequenced at lower depths is difficult to implement for a study based on autopsy samples. Moreover, such a study would need to rely on grouping the samples to carry out comparisons, rather than directly regressing their dN/dS values, which complicates to correctly account for possible confounding factors.

Supplementary Note 8: Analysis of tolerance to errors

Abstract

We estimated that around 4 bases out of every 10^8 bases sequenced are expected to produce artifacts (Supplementary Note 4). In this Supplementary Note we explore the effect that this or higher error rates can have on the calculation of positive selection in the cohort studied here. We developed a robust simulation analysis to inject artifacts with different mutational profiles to synthetic samples bearing mutations with the same trinucleotide probabilities as those observed in the cohort, with predetermined levels of positive selection. We then evaluate how much the calculated positive selection across synthetic samples with errors differs from that built into them. This analysis demonstrates that the injection of errors with different mutational profiles would result in a moderate decrease of the magnitude of positive selection computed with respect to the actual value of the sample. This analysis also shows that there is no chance that a high value of positive selection is computed as a result of artifacts in a sample. In summary, the calculation of positive selection across genes is robust to levels of artifacts higher than the ones estimated in our study.

Tolerance of positive selection calculations to errors

We wondered how artifacts detected in the samples (as they occur randomly across genomic positions) impact the magnitude of positive selection estimated for different genes. We wanted to assess how the dN/dS estimates calculated with Omega across samples could be impacted by artifactual mutations (herein ‘errors’) generated with the error rates estimated from sequencing cord blood.

We simulated synthetic catalogs of mutations (*i.e.*, synthetic samples) whereby two signals are combined: ground truth mutations (obtained through sampling of the mutational profile of a real bladder sample) distributed across synonymous and non-synonymous sites to obtain a pre-defined dN/dS value, and error mutations. The baseline synthetic samples only contained “true” mutations sampled from the mutational profile of the cohort. In different simulations, we then injected increasing levels of artifacts sampled from mutational profiles representing different sources of errors.

Artifacts’ mutation probability vectors

We tested the tolerance of Omega to known frequent sequencing artifacts (SBS43, SBS45, SBS52, and SBS58 in the Cosmic reference catalog of mutational signatures), and to the BotSeq mutational profile in cord blood reported in Abascal *et al.* (Nature 2021)[11], which is enriched in artifactual C>A mutations common to this technology (artifacts hereafter) (Supplementary Fig. 36).

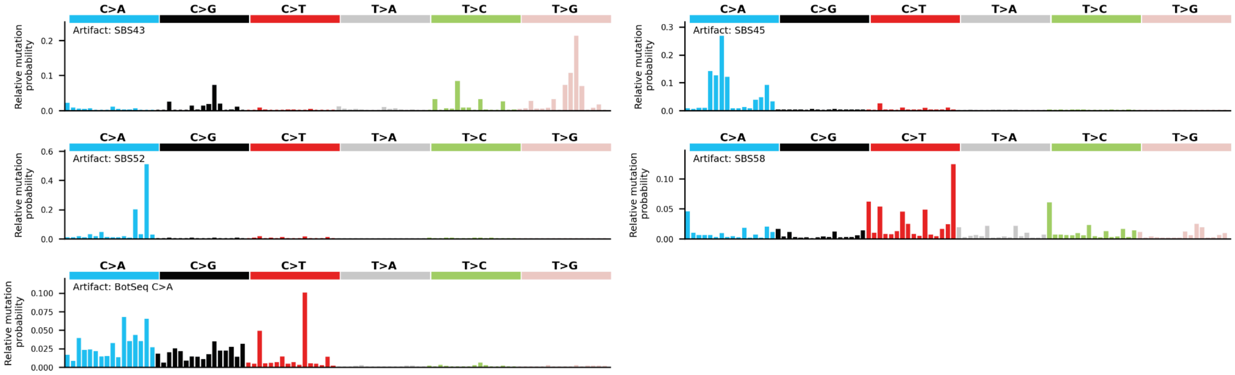

Supplementary Figure 36. Single base substitutions (SBS) profile of 5 artifacts tested as error sources.
The name of each artifact signature in the reference catalog (or ref[11]) is indicated within each plot.

When ultradeep duplex sequencing mixtures of clones, the probability of observing a mutation is significantly impacted by the number of times the given position is sequenced. If we simulate error injections in this type of data, we also need to account for the sequencing depth at each position, as it would impact the probability of an artifact mutation to be sequenced. To make this adjustment in the vector of trinucleotide mutation density, we used the pooled cohort's depths and followed the same approach as in Supplementary Note 6 (see "Relative mutation density per tri-nucleotide context" section).

Synthetic samples

In order to draw simulated mutations with a prescribed neutral mutation density, non-synonymous dN/dS and artifact mutation density, denoted ε and expressed in mutations per sequenced base, we model the mutation density per site (*i.e.* accounting for genomic position and nucleotide change) as being additively compound with a true and an artifact signal, *i.e.*:

$$m_s = t_s + e_s,$$

where t stands for the true mutation density, e is an artifact rate (artifactual mutations consistent with a given artifact mutational signature) and s is the index of the site.

For this simulation exercise, we employ the background mutation density estimates per site $\hat{\mu}_s$ that we inferred to run the positive selection fitting on the real data (Supplementary Note 6). The true mutation density is then $t_s = \hat{\mu}_s$ whenever s is a synonymous site and $t_s = \omega\hat{\mu}_s$ whenever s is non-synonymous, with ω representing the non-synonymous dN/dS.

We define the artifact rate per site e_s as given by the normalized frequency of the trinucleotide context of s in the artifact signature of interest p_s corrected by a factor λ so that the total burden of artifact mutations is consistent with the artifact rate ε, *i.e.*:

$$e_s = \lambda p_s \quad \text{such that} \quad \sum_s e_s = \sum_s \lambda p_s = \varepsilon \sum_s d_s,$$

where d is the number of bases sequenced.

Upon calculation of the m_s for all sites s, we randomly draw mutations from each site as Poisson distributed with mean m_s for a number of replicates (N=100). We create synthetic catalogs of mutations for the entire cohort across a two-dimensional grid of ground truth dN/dS and artifact rate: ground truth dN/dS values were taken from the set $\{1, 2, 5, 10, 50\}$, while artifact rates were taken from the set

$$\{10^{-9}, 5 \cdot 10^{-9}, 10^{-8}, 3 \cdot 10^{-8}, 5 \cdot 10^{-8}, 7 \cdot 10^{-8}, 9 \cdot 10^{-8}, 10^{-7}\}$$

expressed in mutations per sequenced base. For example, an artifact error rate of 10^{-8} implies that in a genomic region of 1,000 bases sequenced at 1000X we expect to find 0.01 artifactual mutations.

With the catalog of mutations obtained we can then run our dN/dS determination method Omega and assess the concordance between the ground truth dN/dS ω and the reconstructed dN/dS $\hat{\omega}$.

Results of the experiment

We calculated the dN/dS of each of the genes probed in this study across a set of synthetic samples with a given ground truth dN/dS (between 1 and 50), and a rate of errors (between 10^{-9} and 10^{-7} per base pair sequenced) injected on top of the true mutations. This calculation was replicated across 100 synthetic samples. As an example, Extended Data Figure 7a shows the reconstructed *dN/dS truncating* for RBM10 after receiving injections of increasing artifactual mutations generated in accordance with the BotSeq C>A mutational profile (Supplementary Fig. 36). We observed that increasing rates of errors decrease the estimated dN/dS. However, even in the most extreme case of injecting one artifactual mutation every 10^7 base pairs sequenced, Omega is capable of reconstructing more than 80% of the ground truth dN/dS (Extended Data Fig. 7a,b).

Extended Data Figure 7c shows the overall reconstruction of dN/dS across 5 artifact sources (see Supplementary Fig. 36), different ground truth values of dN/dS, and genes. As expected, artifactual mutations do not have an effect in the dN/dS estimates when the ground truth value is 1 (*i.e.* no selection). With increasing values of ground truth dN/dS, the reduction in dN/dS increases, although it is comparable across scenarios of high selection (*i.e.* ground truth dN/dS of 5, 10 and 50). Despite minor differences, the trend of underestimation of dN/dS across artifact sources and genes is maintained.

Conclusions

The error rate produced by the ultradeep sequencing technology used in this study is remarkably low, in the order of 4×10^{-8} errors per nucleotide sequenced in cord blood. This analysis shows that with an error rate of this magnitude Omega is capable of reconstructing around 90% of the real dN/dS value of the sample. Moreover, we will not be overestimating dN/dS in any of the scenarios explored.

Supplementary Note 9: Association of risk factors with the normal urothelium clonal landscape

Abstract

We assessed the association of bladder cancer risk factors with the clonal landscape of the normal urothelium. In this supplementary note we describe the workflow followed to this end, including the definition of the metrics of the clonal landscape, the selection of covariates and the linear models used to test the associations. We also describe several controls that demonstrate the association with sex and *dN/dS truncating* of RBM10, ARID1A and STAG2 is robust. Finally, we describe other associations detected between the clonal structure and several clinical factors.

Metrics of the clonal landscape of normal urothelium

Somatic mutagenesis and selection are the two fundamental evolutionary forces that shape the clonal structure of a tissue. In this study, we hypothesized that the clonal structure of the urothelium can be explained in part by sex. To do that, we used 3 quantitative surrogates of the clonal landscape of the normal urothelium that are directly derived from the data we obtained with ultradeep duplex sequencing: non-protein affecting mutation density, protein-affecting mutation density, and dN/dS (see main Methods and Supplementary Note 6). For the TERT promoter, we use the density of likely activating mutations (observed in tumors more than once) and non-activating mutations.

In principle, non-protein affecting mutations are not subjected to selection and thus they constitute a good proxy of the contribution of neutral mutagenesis to the fraction of mutated urothelium. Conversely, the subset of protein-affecting mutations is enriched for variants more likely to be under selection. Positive selection can also be measured more accurately via dN/dS analysis, whereby the excess of non-synonymous mutations over expected is inferred. We focused on specific dN/dS estimates for missense and truncating mutations.

We evaluated the association between these metrics and sex for all the genes except FGFR3, which is not under positive selection at the cohort level, and PIK3CA, for which we compute these metrics only for a specific hotspot. Regarding dN/dS, we excluded from the analysis FOXQ1 truncating, TP53 truncating, RB1 truncating and TERT promoter (computed using activating TERT promoter mutations, as explained in Supplementary Note 6), as we could not compute an estimate for many of the samples (Supplementary Figure 37).

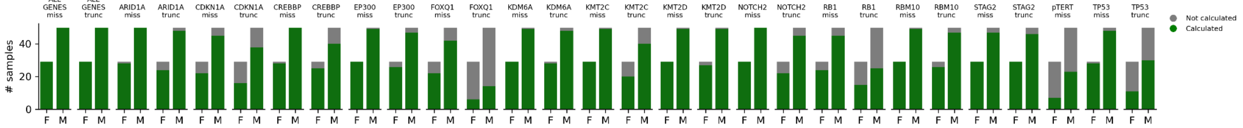

Supplementary Figure 37. Missing dN/dS values across samples for every gene.

For every gene and mutation type (miss: missense, trunc: truncating), the bars represent the number of samples from males or females color coded based on whether dN/dS could be calculated. The green segments represent samples for which we are able to compute dN/dS and the grey segments represent samples with missing dN/dS values.

Selection of covariates

To study the associations between sex, mutation density and dN/dS, we selected a subset of clinical variables collected for the cohort as covariates, including some that are known or suspected to be associated with risk of bladder cancer (e.g. age, exposure to chemotherapy, and body mass index (BMI), Supplementary Table 1). We prioritized covariates based on annotation reliability, correlation with other variables, and balance across the cohort. For example, we did not include *history of urinary infections* in the association analysis as it was not balanced across the cohort. Given the high similarity of the urothelium clonal structure between dome and trigone (Extended Data Fig. 6a-c), we excluded the bladder location as a relevant covariable. We assessed the correlation between continuous variables using Pearson correlation, and the correlations involving at least one categorical variable via logistic regression using McFadden's pseudo-R^2 (Supplementary Figure 38). As expected, *cancer history* and *chemotherapy history* showed a perfect correlation; therefore we chose *chemotherapy history* to be included in the analysis, given the evidence that it promotes somatic mutations, specifically in clonal hematopoiesis[47–49]. We binarized the selected categorical covariates (0 indicating the baseline effect) and scaled BMI and age to 0-1 and decades, respectively.

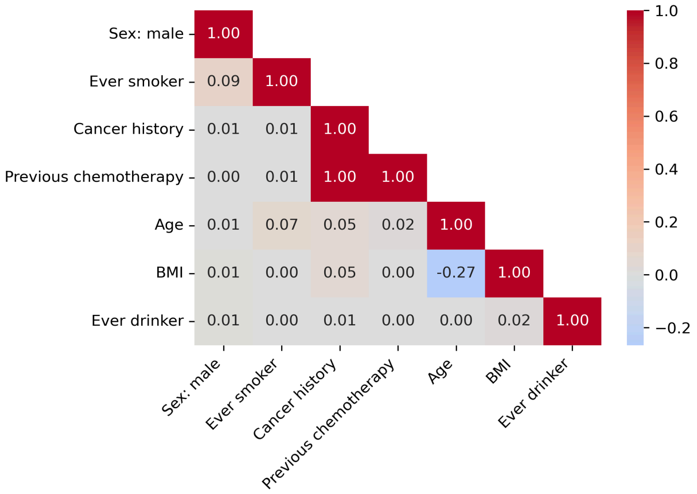

Supplementary Figure 38. Correlogram of clinical variables.

Heatmap reporting the correlation coefficients across selected clinical variables. For comparisons between continuous variables, the Pearson correlation coefficient is calculated; for comparisons involving at least one categorical variable, we report the McFadden's pseudo-R^2.

Linear mixed-effects models

To assess the influence of the selected clinical factors on the surrogates of the clonal structure of the urothelium of individuals, we followed a two-step strategy leveraging the use of linear mixed-effects models (LMEMs) to include all the samples in our cohort. LMEMs are composed of both fixed and random effects and are suitable for cases in which there is not complete independence between data points (in our case, some donors provide two samples). The fixed effects in our models are the clinical variables for which we want to evaluate the association with the urothelium clonal structure. The random effect in our models is the donor of the sample (random intercept). As dependent variables, we use the chosen surrogates of the clonal structure (*i.e.* protein affecting and non-protein affecting mutation density, and dN/dS).

We first built univariate LMEMs to test the independent association between the surrogates of the clonal structure and the clinical variables. Thus for each surrogate we fit one LMEM per gene and clinical variable. We corrected for multiple testing (Benjamini-Hochberg FDR) and deemed as significant all associations below a false discovery rate (FDR) cutoff of 20%. Because the surrogates of the clonal structure are intertwined, therefore not statistically independent from each other, we carried out FDR correction for each surrogate separately.

Although univariate analysis constitutes a first necessary step to resolve the possible causal link between the clinical factors and the clonal structure, it is not sufficient as some clinical factors might confound with one another. Therefore, for those associations reaching significance, we carried out a more stringent multivariate LMEM analysis to discard the potential confounding effects of other clinical variables. We consider as potential confounders any correlated exposures (*i.e.* smoking and sex, smoking and age, and BMI and age), as well as any other significant exposure in the univariate analysis. To assert significance we then apply the same FDR multiple testing correction strategy as described for the univariate models.

In the univariate and multivariate models for age the intercept of the model is set to zero.

Robustness of regressions

We carried out several supplementary analyses to verify that the sex bias inferred for the clonal landscape through the multivariate regression was robust.

Technical variables and sequencing depth

We reasoned that five variables related with the extraction of DNA and the duplex sequencing could potentially introduced some bias in the results: the type of sequencer used (NovaSeq 6000 or NovaSeq X Plus), the degree of DNA integrity upon extraction (measured through the DNA Integrity Number, or DIN), the number of days between death and sample collection, the number of days between sample collection and DNA extraction, and the depth of sequencing. As the first four variables can be potentially associated with increased errors that could be fixed as artifactual mutations, we first verified whether there is any discernible relationship between the mutation density observed across samples and any of these variables (Supplementary Fig. 39). Despite certain trends observed, we only identify a slight significant increase in the mutation density of samples sequenced with the NovaSeq 6000 machine.

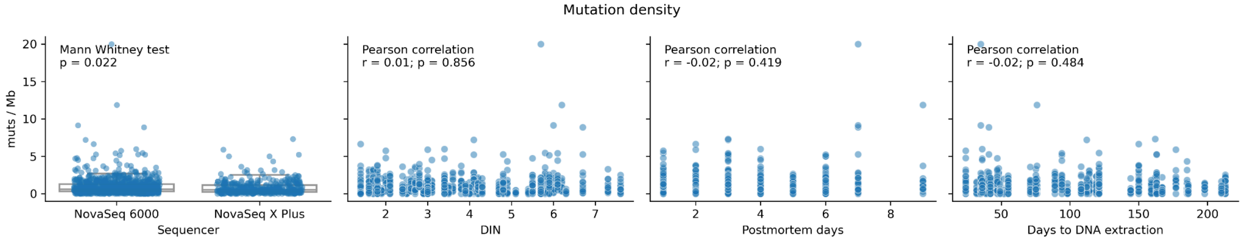

Supplementary Figure 39. Comparison of mutation density across samples defined by differences in technical variables.

For categorical variables, the p-value (p) of a Mann Whitney test is shown. For continuous variables, the coefficient (r) and p-value (p) of the Pearson correlation test are shown.

Next, we explored whether the values of dN/dS computed for the four genes showing a trend of association with sex exhibit any relationship with these variables (Fig. 3e). Overall, we observed a trend of increased dN/dS values in samples processed with the NovaSeq 6000 machine, although this difference is not significant for any gene (Supplementary Fig. 40). Regarding the depth of sequencing, we observe a trend of decreased dN/dS values at higher sequencing depths, although this is only significant for RBM10 (Supplementary Fig. 41).

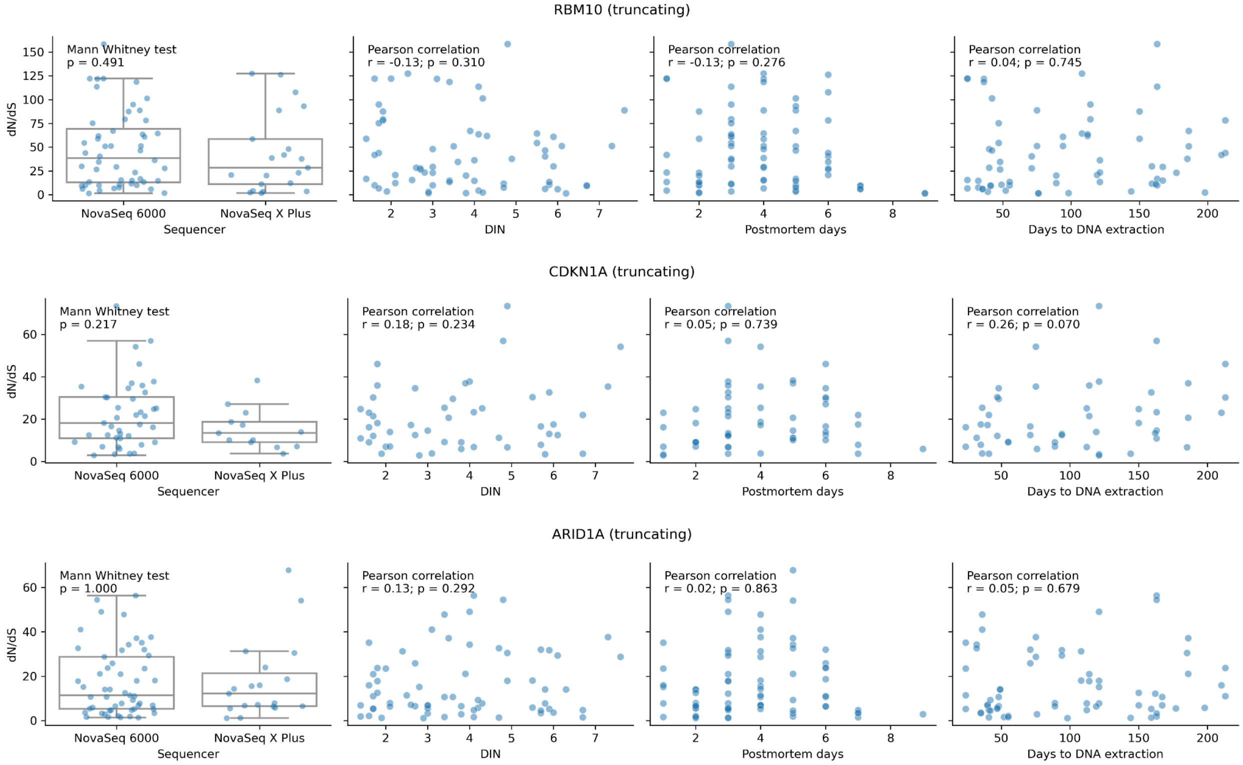

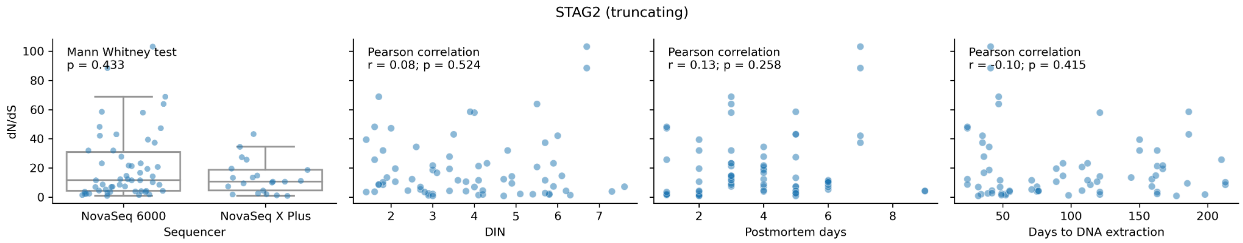

Supplementary Figure 40. Comparison of dN/dS values for genes with significant differences between males and females with technical variables.
For categorical variables, the p-value (p) of a Mann-Whitney test is shown. For continuous variables, the Pearson correlation coefficients (r) and corresponding p-values (p) are shown.

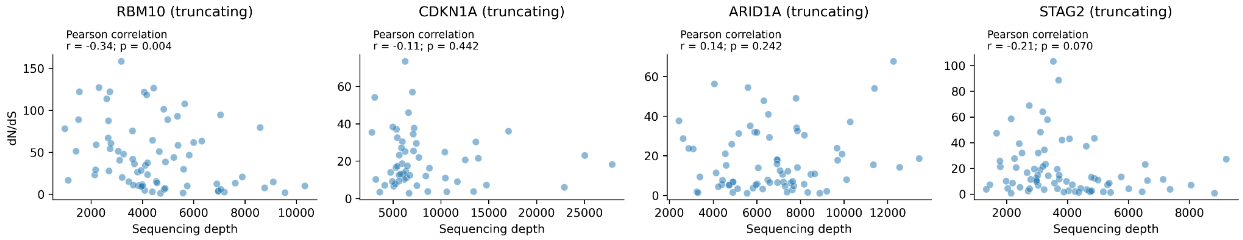

Supplementary Figure 41. Relationship between *dN/dS* and depth of sequencing for genes with significant differences between males and females. The coefficient (r) and p-value (p) of the Pearson correlation test are shown.

To verify any potential confounding effect of these technical variables on the results of the regressions, we repeated the analysis including each of them separately as covariates (Supplementary Fig 42). We observed that the calculated effect size for sex of ARID1A, RBM10 and CDKN1A are similar in range to those observed without including technical variables, and that in all the cases the association is significant (FDR<0.2) except for STAG2, which also had high effect size values but a non significant association in the analysis without technical covariates (Fig. 3e).

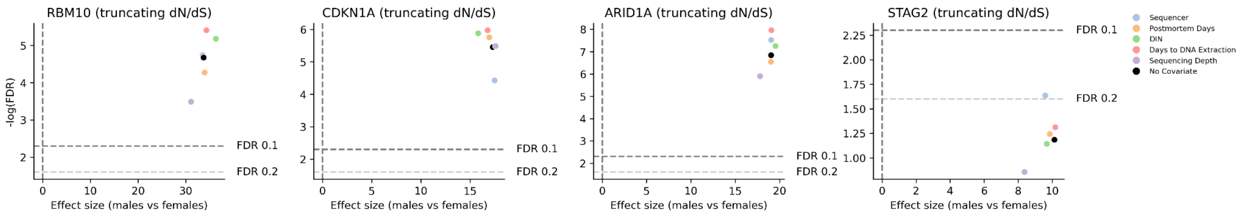

Supplementary Figure 42. Effect of technical variables on the association between sex and dN/dS.
Scatter plots showing the effect size (x-axis) and false discovery rate (FDR, y-axis, in log-scale) of the multivariate association between dN/dS and sex when accounting for each of the technical covariates separately in the genes where we report a sex association. FDR thresholds of 0.1 and 0.2 are depicted in the figure as grey dashed lines, as well as an effect size of 0.

Balance between male and female groups

Unbalanced groups can bias associations deemed significant in linear models. As there are more male than female subjects in the cohort (28 males providing 50 samples and 17 females providing 29 samples), we decided to carry out the analysis in a smaller cohort with a balanced number of male and female samples. To that end, we randomly subsampled by excluding sets of 20 samples from males, and recomputed the regression on these balanced cohorts (50 times). In all these balanced regressions we verified the significant association between dN/dS and sex for ARID1A, CDKN1A (only 6 replicates FDR<0.2), and RBM10 (only 4 replicates FDR<0.2). The loss of significance in these few replicates was likely due to the smaller sample size and thus lower power, but the effect size was similar to that observed in the complete cohort. For STAG2, the majority of replicates were around the significance in the full cohort, which is already borderline (Supplementary Fig. 43).

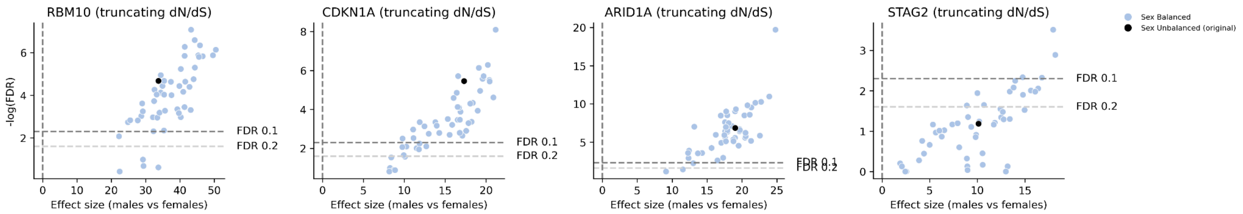

Supplementary Figure 43. Sex associations with dN/dS persist in the sex biased genes upon balanced subsampling.

Scatter plots showing the effect size (x-axis) and false discovery rate (FDR, y-axis, in log-scale) of the multivariate association between dN/dS and sex in the 50 sex-balanced replicates, in the genes where we report a sex association. FDR thresholds of 0.1 and 0.2 are depicted in the figure as grey dashed lines, as well as an effect size of 0.

Exclusion of samples exposed to chemotherapy

We also checked whether the samples belonging to subjects previously exposed to chemotherapy could be biasing the sex association with dN/dS, although this variable was taken into account in the multivariate analysis whenever it was found significant in the univariate analysis. In the regression conducted with the smaller cohort resulting from the exclusion of those samples, we obtained a significant association between the dN/dS and sex for ARID1A, CDKN1A and RBM10 (Supplementary Fig. 44). For STAG2 we reported a high effect size although not significant, as with the full cohort (Fig. 3e).

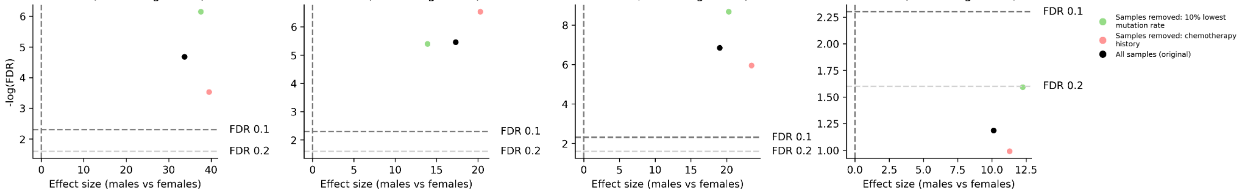

Supplementary Figure 44. Sex associations with dN/dS persist after excluding samples from donors exposed to chemotherapy or excluding samples with the 10% lowest mutation density.

Scatter plots showing the effect size (x-axis) and false discovery rate (FDR, y-axis, in log-scale) of the multivariate association between dN/dS and sex when excluding subjects with a chemotherapy history in the genes where we report a sex association. Results are also shown for the exclusion of samples with the 10% lowest mutation density. FDR thresholds of 0.1 and 0.2 are depicted in the figure as grey dashed lines, as well as an effect size of 0.

Exclusion of samples with lower mutation density

Assuming a fixed rate of errors introduced by the DNA duplex sequencing technology, samples with lower mutation density should have higher percentage of erroneously called mutations, although the similarity of their mutational profile to that of the entire cohort suggests this is not abnormally high (Supplementary Note 4). Regardless, to guarantee that the presence of samples with low mutation density was not influencing the results of the regression, we recalculated them excluding the 10% (8) samples with the lowest mutation density (3 from males and 5 from females). In the regression conducted with the smaller cohort resulting from excluding these samples, we obtained a significant association between the dN/dS and sex for ARID1A, CDKN1A and RBM10. In the case of STAG2, we report a similar effect size and borderline significance, as with the full cohort (Supplementary Fig. 44).

Potential sex bias in the exposure to smoking

We checked the potential confounding effect of smoking for the association between sex and dN/dS truncating for RBM10, CDKN1A, ARID1A and STAG2 and observed a maintained significant effect. Given that our smoking variable only takes into account the smoking history (never smokers *vs* ever smokers), we are not considering the effect of the amount of smoking consumption, especially if this would be different between the males and females in our cohort. As data on the smoking packs per year of the individuals in our cohort was not available, we used the ratio of median smoked packs per year between males and females calculated with the UK Biobank data, which is 1.32 (Supplementary Fig. 45a). We recodified the smoking history variable as *{never smoker: 0, ever smoker female: 1, ever smoker male: 1.32}*. For RBM10, CDKN1A and ARID1A we reconstruct a similar effect size and significance. For the case of STAG2, we get a similar effect size and borderline significance, as we see when smoking is not adjusted by the potential bias in the amount of consumption between males and females (Supplementary Fig. 45b).

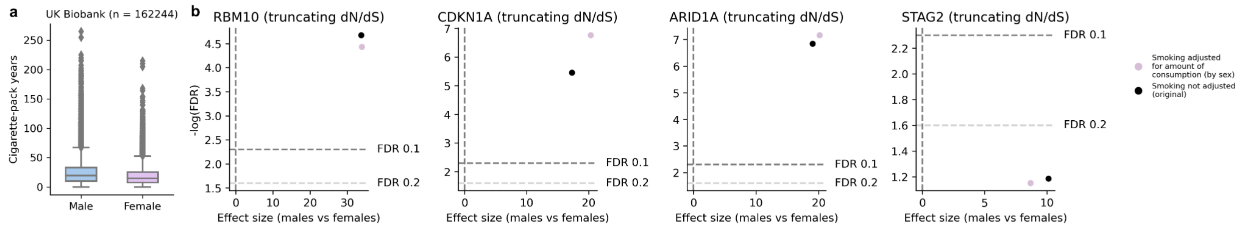

Supplementary Figure 45. Analysis of the effect of a potential sex bias on the exposure to smoking in the differential positive selection of genes in males vs females.

a) Number of cigarette-pack years consumed by males and females in the UK Biobank cohort

b) Scatter plots showing the effect size (x-axis) and false discovery rate (FDR, y-axis, in log-scale) of the association between dN/dS across four genes and sex, correcting for smoking as a binary variable

(dark-colored dots) or using the magnitude of the ratio of median male/female cigarette-pack years obtained from the UK Biobank cohort (light-colored dots)

In the regression plots in b, the color legend is the same as in previous plots.

Genes in the X chromosome

As females have double the amount of X chromosomes than males, it is expected that sequencing any gene in this chromosome would yield higher depth in females than males. We verified this is the case for the three genes in our panel that are in the X chromosome (RBM10, KDM6A and STAG2; Supplementary Fig. 46). While the magnitude of positive selection on truncating mutations in two of these genes (RBM10 and STAG2) appears associated with sex, we found no association for KDM6A, for which there have been reports of different mutation density in bladder cancers from males and females[50].

We observed a slight increase in the number of missense and truncating mutations in females in KDM6A, which is probably due to the higher sequencing depth in females. However, we did not observe any sex bias in the mutation density or dN/dS between males and females for KDM6A (see results of regressions in Supplementary Table 7). Instead we observed a higher density of protein affecting mutations and truncating dN/dS values in RBM10 and STAG2 in males.

We also checked whether the non-protein affecting mutation density for the genes in the X chromosome was higher in females than in males. As the majority of genes in one of the copies of the X chromosome are imprinted[51], it is possible that females have a bigger load of neutral mutations. Although there is a tendency of a higher non-protein affecting mutation density in females in RBM10 and STAG2, this effect is not significant when assessing it in a mixed-effects multivariate linear regression (Supplementary Table 7).

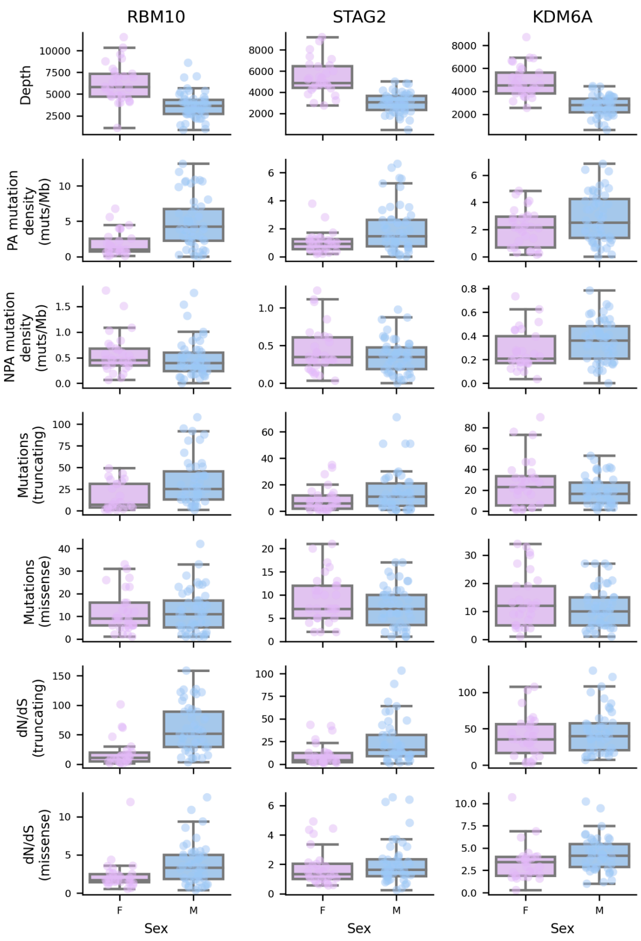

Supplementary Figure 46. Distribution of several features computed in our study for the three genes in the X chromosome for samples obtained from male and female donors.
PA = protein-affecting, NPA= non-protein affecting.

Other observed associations

Below, we describe and discuss several other associations detected between the density of mutations (and/or the magnitude of positive selection) and different clinical annotations. We only describe those that we deem more relevant, or which we are immediately capable of explaining. Other associations detected in the study (see Supplementary Table 7) will require further studies on larger cohorts to tease apart. That is the case, for example, of a potential negative association of CDKN1A *dN/dS truncating* values with a history of smoking (Supplementary Table 7).

The effect of chemotherapy

We observed that the *dN/dS* truncating of several genes, such as KDM6A, RBM10, STAG2 and KMT2D, as well as that computed across all genes appeared significantly (negatively) associated with prior exposure to chemo/radiotherapy (Supplementary Table 7). The reason for this was a significant positive association of the non-protein affecting mutation density of these genes with prior exposure to chemo/radiotherapy and a lack of association of the protein

affecting mutation density with this clinical feature. We reasoned that upon exposure to mutagenic chemo/radiotherapy, the density of non-protein affecting mutations across the urothelium is expected to suffer a sudden increase. If the *dN/dS* is computed soon after this exposure, it will be lower than that of a non-exposed individual because of the excess of non-protein affecting mutations introduced by chemotherapy. With the action of selection over time, however, it is reasonable to expect that clones bearing chemo/radiotherapy induced protein-affecting mutations will expand, resulting in a growth of the *dN/dS* with respect to unexposed individuals.

It is important to not lose sight of the meaning of the metrics of selection. The negative association of *dN/dS* values with chemo/radiotherapy does not imply less selection on mutations of these genes in exposed individuals versus their non-exposed counterparts. It is just an effect of the sudden exposure to a strong mutagenic source.

Association with mutation burden

We calculated a separate metric that takes into account the density of protein affecting mutations and the number of mutant reads carrying each mutation, which we refer to as "mutation burden". In duplex sequencing, each duplex read corresponds to a unique DNA molecule and therefore the number of duplex mutant reads for a given mutation is proportional to the size of the mutant clone. Thus, the mutation burden takes into consideration not only the number of mutations but also the size of the mutant clones. Regressing the clinical features of donors on this metric we identified several genes with the mutation burden significantly associated with sex, and other features. The associations largely overlap those observed using dN/dS and the density of protein affecting mutations (Supplementary Table 7), which is not surprising, given that most genes in the panel act through loss of function and the fraction of the urothelium covered by mutations affecting them is, in general, rather small (Extended Data Fig. 5b)

Supplementary Note 10: Statistical power analysis

Abstract
When conducting any type of statistical study, it is important to understand the statistical power profile implicit to the study design, which informs the likelihood that the test yields a significant association whenever there is a true underlying association with a given effect. Here we calculate the power profile of the linear mixed-effects univariate association analysis and use this profile to gain more insight about the robustness of the association between sex and dN/dS. The conclusion of this calculation is that this study has the power to detect true associations at the effect sizes we observe in the three sex biased genes. It also reveals that at the baseline dN/dS truncating we observe in these three genes it is extremely unlikely that the effects observed occur by chance due to the cohort composition.

Power analysis strategy
We conducted a study to characterize the statistical power and false positive rate of the univariate association study with the dN/dS estimates calculated with omega as response variable and either of the variables “sex” and “history of smoking” (variables of interest) as explanatory variables. To this end we simulated random datasets with distributional properties and composition comparable to the actual dataset. In particular, we study the range of between-group differences and within-group dispersions that we assert from the actual data. By conducting regression on the synthetic datasets thus generated, we can provide estimates of the statistical power and false positive rate.

Explanatory variables of interest
Both “sex” and “history of smoking” are dichotomous categorical variables: the variable “sex” has levels “male” and “female”, while the variable “history of smoking” has levels “never” and “ever”, where “ever” indicates samples annotated as either current or former smokers (Methods and Supplementary Note 9).

Regarding history of smoking, the actual cohort has 51 samples (from 29 donors) annotated as “ever” and 28 samples (from 16 donors) annotated as “never”. Regarding sex, the actual cohort has 50 samples (from 28 donors) annotated as “male” and 29 samples (from 17 donors) annotated as “female”.

Learning the features of our dataset
In order to provide a dataset with simulations that resemble as closely as possible to the actual in terms of statistical features, we looked into three main aspects of our dataset: i) the distributional properties of dN/dS across samples, genes and consequence types; ii) the dispersion of the random intercepts associated with samples coming from the same donor; iii) the effect of age on dN/dS.

dN/dS distribution

We observed that in our dataset, the mean and standard deviation of dN/dS ratios across genes were proportional for truncating as well as missense mutations, indicating that genes with more selection (higher dN/dS) had more variability across samples. More concretely, if μ and σ are the respective sample mean and standard deviation of the dN/dS values specific for a given gene and consequence type (either “missense” or “truncating”), the proportion $\sigma = k\mu$ holds (Supplementary Fig. 47), where k only depends on the consequence type, not on the specific gene. From the data we infer that k(missense)~0.9 and k(truncating)~0.75. We used this property to generate realistic synthetic values of dN/dS values attached to different groups of samples (see section “Random generation of datasets” below).

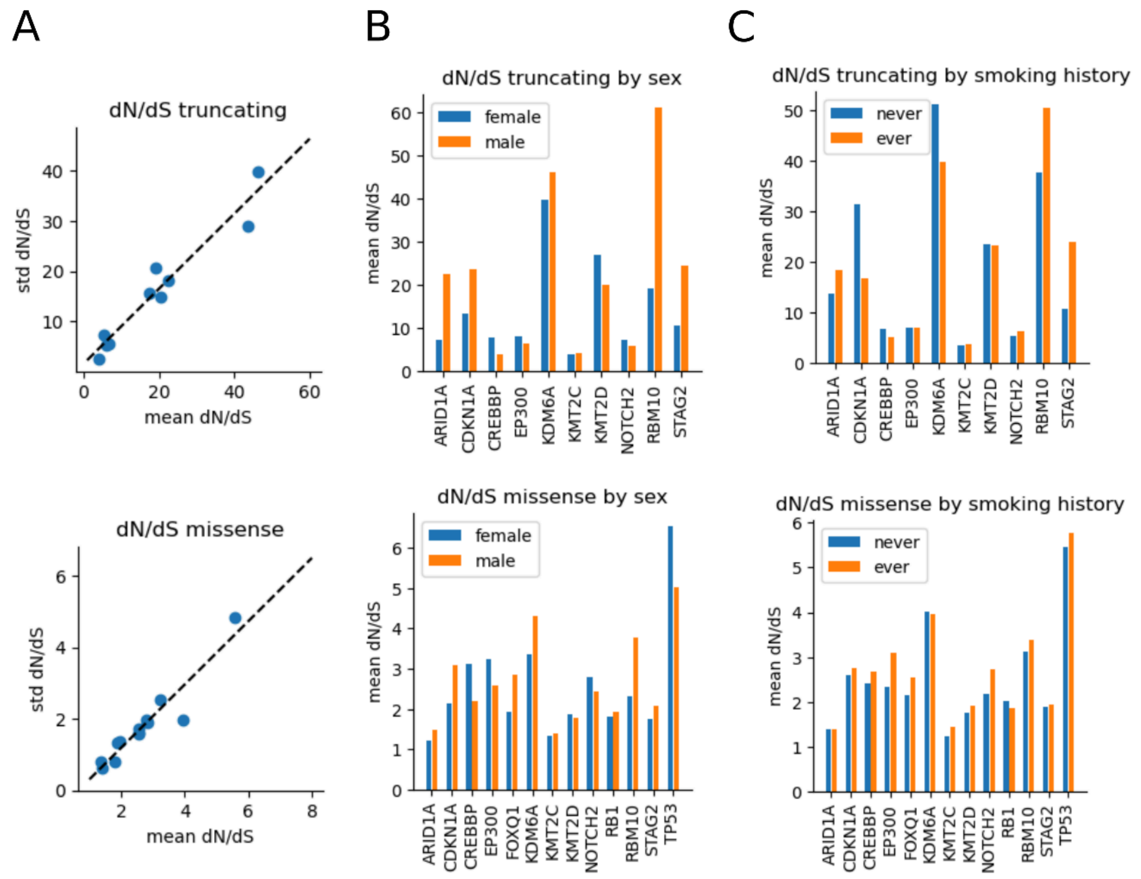

Supplementary Figure 47. Analysis of the underlying distribution of dN/dS values in the cohort. A) A mean-standard deviation proportionality holds in the context of dN/dS truncating and missense, respectively. B) Mean dN/dS values per gene and group category by sex. C) Mean dN/dS values per gene and group category by history of smoking. Female and never smoker levels are considered baseline in the regression analysis by sex and history of smoking, respectively.

Dispersion of random intercepts

We conducted univariate linear mixed-effects regression with dN/dS as response variable, the variable of interest and the donor's age as fixed effects, and the donor as a random intercept, i.e. using R-style notation:

```
dN/dS ~ variable_of_interest + age + (1|donor)
```

We conducted the fitting with Python's function `statsmodels.formula.api.mixedlm`, from which we can retrieve the estimated standard deviation s of the random intercept variable "donor". For each consequence type, we conducted the regression for all the possible combinations of gene and variable of interest, collected all the dispersion estimates (Supplementary Fig. 48) and computed the median donor random intercept across genes: for smoking history s(missense)=1.2 and s(truncating)=11.4 for consequence type missense and truncating, respectively, whereas for sex s(missense)=1.1 and s(truncating)=10.3.

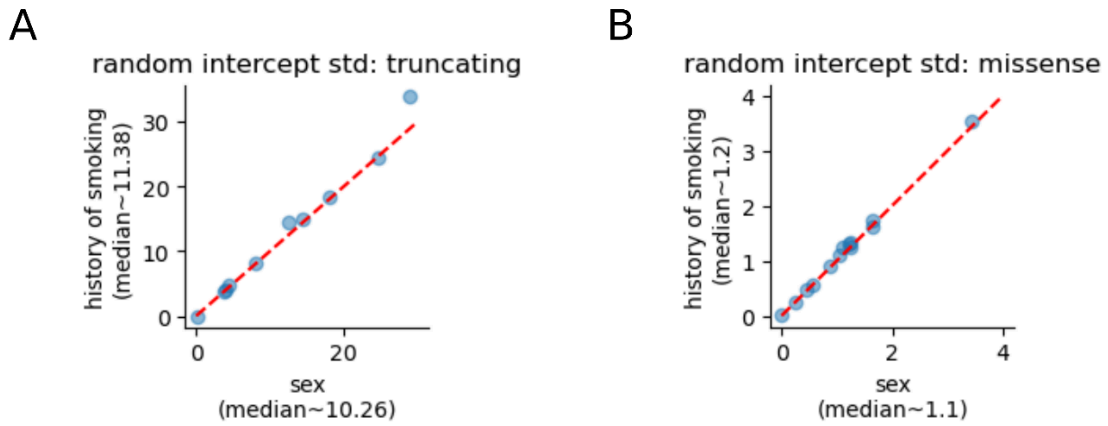

Supplementary Figure 48. Standard deviation of the underlying distribution of donor random intercepts across genes inferred from the univariate linear mixed-effects model fitting from the actual data with the variables sex and history of smoking.
A) dN/dS truncating and B) dN/dS missense.

Age-dependent effect

In our simulations it will be important to introduce the effect of the variable "age" as it can be a confounding factor of the association with the variables of interest in the real data (Supplementary Fig 49).

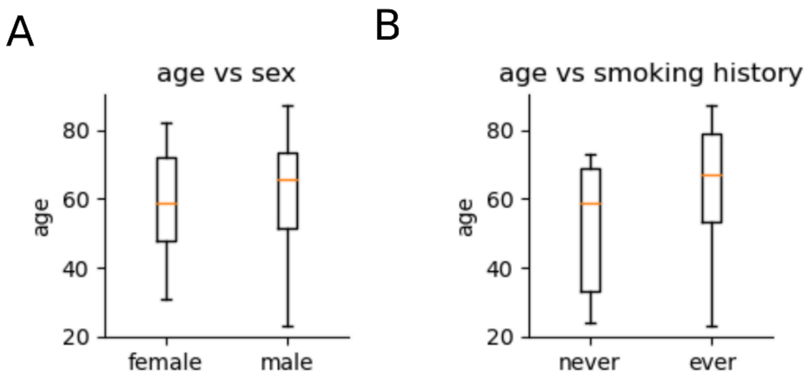

Supplementary Figure 49. Age distribution per group category in sex (A) and history smoking (B). Intuitively, age has a higher confounding potential in the association analysis with smoking.

In order to choose a realistic age-dependent effect for the simulations, we collected the age effects across genes from the univariate linear mixed-effects regressions using age as explanatory variable, i.e. in R-style notation:

```
dN/dS ~ age + (1|donor)
```

Taking the distribution of age effects across genes for missense and truncating consequence type and computing their medians yields 0.007 dN/dS units per year both in the missense and truncating context.

Random generation of datasets

We generate random synthetic replicates of the dataset to conduct regression analysis that reflect the main characteristics of the actual data used to conduct the following association analyses:

dN/dS truncating ~ sex
dN/dS missense ~ sex
dN/dS truncating ~ history of smoking
dN/dS missense ~ history of smoking

Each replicate thus consists of a collection of synthetic samples, each represented by a vector of features (variable of interest, age and donor) alongside a dN/dS value that is randomly drawn from a distribution that depends on the features of the sample. Furthermore, the dataset replicates for each association will reflect the same composition as in the actual cohort (number of samples per group as well as the consistency between group and donor).

More concretely, we make the dN/dS of each sample depend on 1) a group-specific (male/female or never/ever smoker) component, 2) an age-dependent component, and 3) a donor-specific random effect. Moreover, each replicate will also depend on a prescribed

baseline effect, so-called μ, as well as an effect size e representing the expected dN/dS difference between sample groups by variable of interest level (sex or history of smoking).

For each of the four association setups, we generate N=100 replicates for each value (μ, e) within a grid of configurations, baselines μ (grid of 20 values from 1 to 20 in missense, grid of 20 values from 1 to 50 in truncating) and effects e (grid of 21 values from 0 to 10 in missense, grid of 41 values from 0 to 40 in truncating), amounting to a total of 124,000 simulations.

Group component

For a sample within a baseline group with mean μ, the group specific component is drawn from a gamma distribution that has mean μ and standard deviation $\sigma = k\mu$, with a suitable choice of k depending on the study case, as described above: we denote this distribution $\Gamma(\mu)$. For a sample belonging to the other group, where e is the mean difference between groups, we will draw samples from the distribution $\Gamma(\mu + e)$. We say that μ is the baseline in this comparison.

Donor component

For each donor we draw a donor random intercept from a normal distribution with zero mean and standard deviation given by s(missense)=1 and s(truncating)=10, for consequence type missense and truncating, respectively, as described above.

Age component

For each donor we will derive the dN/dS component explained by the age t simply as $a \cdot t$, where a stands for the effect size of the age, as described above, with a=0.007 units per year both in missense and truncating context.

In sum, for a given configuration (μ, e) and sample, we randomly draw values of dN/dS according to the following formula

$$dN/dS = 1 + g + at + d$$

where either $g \sim \Gamma(\mu)$ or $g \sim \Gamma(\mu + e)$ depending on whether the sample belongs to the baseline group or not, where t represents the age of the donor measured in years, and $d \sim N(0, s^2)$ is the random intercept of the donor.

Overdispersed synthetic dN/dS

Although this simulation strategy is convenient, we must observe on a technical note that it produces a slightly higher dispersion on the synthetic dN/dS values than observed in our cohort, the reason being that the distribution we draw g already owes part of its dispersion to the age and the donor structure of the data. As a result, the synthetic dN/dS will have a slightly higher dispersion compared to the real data, thus making it more difficult for the regression methods to reach significance. Therefore, our method to estimate the power is expected to cast a conservative underestimation.

Linear mixed-effects reconstruction

Accordingly, for each configuration we fit a linear mixed-effects model, with dN/dS as a response variable, the variable of interest and the donor as a random intercept, i.e. in R-style notation:

```
dN/dS ~ variable_of_interest + (1|donor)
```

For the variable of interest we retrieve the effect and the p-value.

Statistical power

We define the *statistical power* at a specific alternative hypothesis configuration as the proportion of replicates for which the fitting yields significance, which we set as a variable of interest p-value < 0.05.

Although there are differences in the cohort composition split by sex compared with history of smoking, which a priori justifies a separate analysis (age has more potential to confound the history of smoking analysis) this distinction does not seem to produce sharp differences in the power profile across baselines and effects (Supplementary Fig. 50).

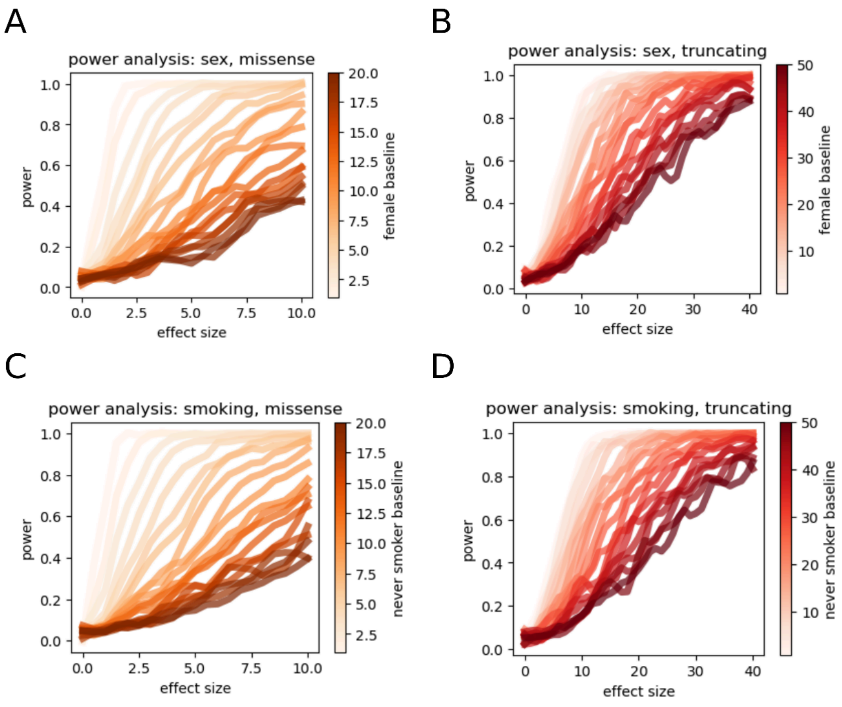

Supplementary Figure 50. Statistical power profiles computed with our simulation strategy.
We represent 20 curves, each representing the baseline (mean dN/dS of the lower group) in steps of dN/dS=1 (missense) and dN/dS=2.5 (truncating).

Power to discover sex associations

For all the genes and consequence type combinations we conducted a univariate linear mixed-effects regressions with our data and compared the results with the theoretical power and false positive rate that would correspond in each case given the baseline and inferred effect of the variable of interest. We also computed the proportion of simulations with the same baseline and zero ground truth effect that yielded an effect higher or equal than the one inferred, which we refer to as “effect p-value”: it plays the role of a false positive rate metric whereby we assess the likelihood that effects as high as the observed are reached just by chance due to the data composition.

In Supplementary Table 10 we show the results of the comparison for sex association for those genes that we deem robust associations upon univariate, multivariate and FDR correction in the study: ARID1A, CDKN1A, RBM10 in the context of truncating dN/dS. We also include STAG2 in the context of truncating dN/dS as it shows an intriguing trend, albeit non-significant. The power of the tests is high (>0.75) for three genes, and moderate for one (CDKN1A truncating). Importantly, none of the 100 simulations with ground truth zero of effect (meaning no difference between males and females) reach an effect size as big as the one observed, indicating that these results are unlikely to be false positives (p-value <0.01) (Supplementary Fig. 51).

Supplementary Table 10. Statistical power to test association of the clonal landscape with sex

GENE	CSQN	ESTIMATE	CI_LOW	CI_HIGH	PVAL	INTERCEPT	COVARIATE	BASELINE	POWER	EFFECT_PVAL
RBM10	truncating	39.92	19.20	60.64	<0.01	17.22	is_male	19.22	1.00	<0.01
ARID1A	truncating	15.47	6.97	23.97	<0.01	6.59	is_male	7.27	0.99	<0.01
STAG2	truncating	13.23	1.68	24.78	0.025	9.85	is_male	10.42	0.90	<0.01
CDKN1A	truncating	9.28	-0.049	18.62	0.05	13.87	is_male	13.33	0.57	<0.01

Supplementary Table 10 Summary of the three robust associations (green) and the one non-significant trend (orange) found between dN/dS and sex in the study. CSQN: Either missense or truncating, represents the specific dN/dS used as response variable in the association analysis. ESTIMATE: Coefficient of the binary variable of interest (“is_male”) inferred via linear-mixed effects regression against dN/dS using the donor as a random intercept. CI_LOW, CI_HIGH: Lower and upper 95% CI bounds of ESTIMATE. PVAL: p-value associated with the variable of interest in the regression analysis. INTERCEPT: Inferred intercept in the regression analysis. COVARIATE: The (binary) explanatory variable representing sex. BASELINE: Average CSQN-specific dN/dS value in the baseline group of samples (female). INTERCEPT and BASELINE are expected to follow closely one another. POWER: Statistical power corresponding to the BASELINE and ESTIMATE in the power profile. EFFECT_PVAL: The “effect p-value” is an ad-hoc metric that we defined as the proportion of times the sex coefficient attains a value at least as high as ESTIMATE upon regression with a dataset corresponding to BASELINE and zero ground-truth effect. It can be thought of as an effect-aware false positive rate.

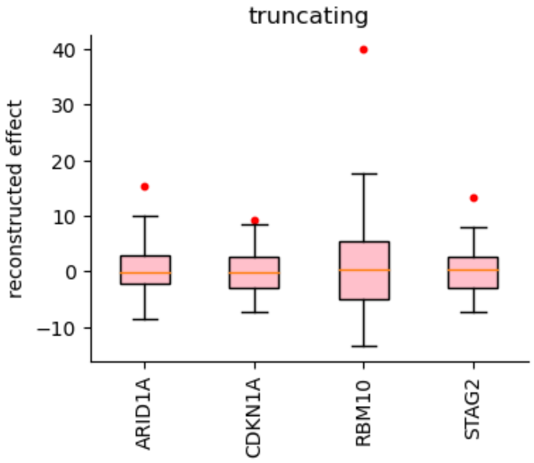

Supplementary Figure 51. Reconstructed baseline effect size of the association between the positive selection on truncating mutations in genes and sex
Boxplots represent the distributions of reconstructed male bias effects from the simulated data when the ground truth male bias effect is zero. For each gene the data has been randomly generated in accordance with the mean truncating dN/dS values in the female cohort. Red dots represent the effect inferred with the real data after univariate linear mixed-effects regression for ARID1A, CDKN1A, RBM10 and STAG2 for truncating dN/dS.

This has two important implications regarding our study design: 1) the cohort is adequate to identify associations as strong as the ones observed in these four genes, 2) the structure of the cohort makes it very unlikely that such strong effects show in the regression analysis just by chance, which further reinforces the conclusion that these four associations are real.

pTERT association with smoking is not confounded by depth

We find a strong association between the presence of activating pTERT mutations and the interaction between age and smoking. We wanted to assess the robustness of this result to changes in sequencing depth across samples. In our cohort we observed a moderate preference towards higher sequencing depth in pTERT in the smokers group (Supplementary Fig. 52) which could in principle imply that more mutations are observed even with the same underlying mutation density across groups, thereby potentially leading to spurious associations (or lack of). To make sure this is not confounding our analysis, we devised a test to assert how much the sequencing depth influences the number of mutations we observe in pTERT.

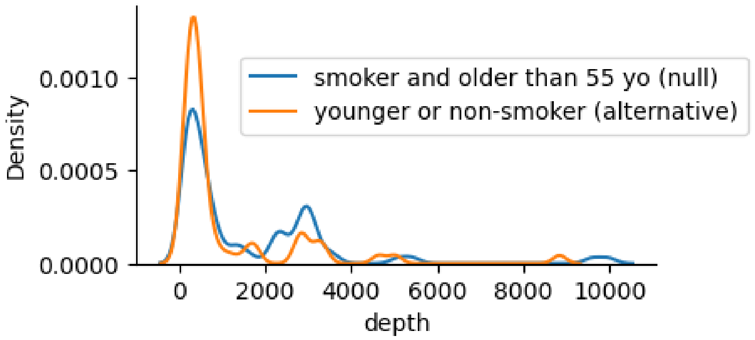

Supplementary Figure 52. Density plot showing the depth differences between the null group of samples (smoking history, older than 55 yo) and the alternative group (either younger than 55 yo or without smoking history) in the two pTERT sites of interest: chr5:1295113 and chr3:1295135.

Method

For the sake of the test's simplicity and consistency, we draw our attention towards the two well known mutational hotspots of pTERT: chr5:1295113 and chr5:1295135 (GRCh38) denoted H1 and H2, respectively. These are highly mutated positions among smokers for which we can a priori provide a more accurate estimation of the mutation density in the null group, i.e., the samples from donors aged > 55 years old with a history of smoking.

We proceed by making an initial guess of the mutation density at the hotspots in the null group, which we compute as follows:

$$\tilde{\mu}_1 = \sum_s a_{s1} / \sum_s d_{s1}, \quad \tilde{\mu}_2 = \sum_s a_{s2} / \sum_s d_{s2}$$

where a_{si} and d_{si} denote the alternate depths and depths in sample s at hotspot i, and where the sums run across samples in the null group.

While these are reasonable estimates if we assume that the mutation density is homogeneous across samples at the same hotspot, the lack of homogeneity may imply the paradoxical fact that with these estimates the expected number of times these hotspots are found mutated in the null group is much higher than the observed. This is exactly the case.

Instead, we recalibrate to yield mutation density μ_1 and μ_2 such that they satisfy the following:

1) $\tilde{\mu}_1/\tilde{\mu}_2 = \mu_1/\mu_2$
2) μ_1 and μ_2 yield an expected number of times that the hotspots are found mutated in the null group equal to the observed in the null group.

With these estimates, we can then test whether the number of times the pTERT hotspots are found mutated in the alternative group (composed with samples from either younger or non-smoking donors) is smaller than expected. If we model the number n of alternate reads at a hotspot site sequenced with depth N and mutation density p as binomially distributed, where N

is the number of trials and p is the probability, we can conduct a test by randomly sampling alternate read counts from each hotspot-sample for samples in the alternative group. If the count for a sample hotspot is ≥ 1, we count the hotspot-sample once (i.e. we binarize the value drawn from the binomial distribution and sum across hotspot sites and samples). By sampling several times, we can compute the distribution of the number of hotpot-samples mutated and derive an empirical p-value by comparing it with the observed number of hotspot-samples.

Results

Upon randomization, the distribution of randomized values is significantly higher than the observed value (n=3) in the alternative group. For consistency, we repeated the same randomization experiment with reduced mutation density resulting from the ones inferred from the null group by multiplying by 1/f, where f is what we define as "reduction factor". Only after reducing the mutation density inferred from the null group between 10-20 fold the significance was lost. We conclude that the differences in number of mutations observed in the hotpots between the two groups are not explained by the differences in sequencing depth (Supplementary Fig. 53).

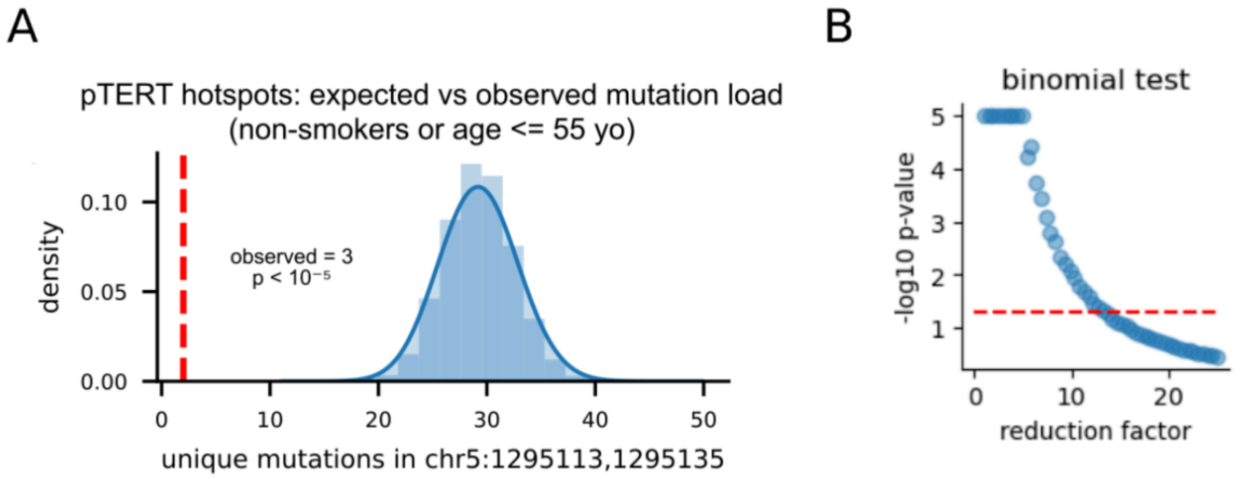

Supplementary Figure 53. Observation of pTERT activating mutations across young and/or never smoker donors is not explained by lower depth.

(A) Density plot showing the null distribution of times a pTERT hotspot would be found mutated in the simulated cohort of non-smokers or younger than 55 yo upon 10,000 randomizations, drawn as the sum of binarized binomial distributions. The red dashed line represents the number of observed events, which was never attained across randomizations, hence an empirical p-value < 1/10,000. (B) Curve of p-values vs reduction factors applied on the mutation density inferred from the null group.

Supplementary Note 11: Comparison of normal urothelium and bladder tumors

Abstract
We explored the differences of mutation density of the genes included in the study between bladder cancer and normal urothelium. We observed different relative mutation density across bladder cancer subtypes and normal urothelium. We also see RBM10 and CDKN1A more frequently mutated in bladder tumors of males, resembling the sex bias in the normal urothelium. In contrast, TERT promoter activating mutations do not show an increase in bladder tumors from smokers although they appear more frequently in older individuals.

Different relative mutation frequency in normal urothelium and bladder cancer
Ultradeep sequencing of a mixture of clones of the normal urothelium of 79 samples rendered approximately 16-fold more mutations than those detected on the same set of genes across close to 900 bladder tumors (Fig 1b). The difference in the absolute number of mutations identified can be explained by the high sequencing depth and the fact that we are not just sequencing one clone (plus subclonal mutations at sufficiently high variant allele frequency) as we do in tumors, but rather a collection of expanded clones that colonize the normal tissue. In addition to observing a much larger number of mutations in normal urothelium compared to bladder tumors, there is a striking difference in the ranking of most mutated genes (Extended Data Figure 9a).

For example, FGFR3 is infrequently mutated in the normal urothelium compared to other genes in the panel and appears under negative selection for truncating mutations. However, it is the most mutated gene among those analyzed in non-muscle invasive bladder tumors (NMIBC) but not frequently mutated in the muscle invasive subtype (MIBC). There is no mandatory pathogenesis pathway from NMIBC to MIBC, although this transition can occasionally take place[2]. The depletion of FGFR3 clones in the normal bladder and its frequent presence in non-muscle invasive tumors could be pointing to FGFR3 being a driver in the evolution towards NMIBC but not necessarily to MIBC. On the other hand, TP53 is a gene moderately mutated in the normal urothelium, compared to the other genes in the panel, and under positive selection. However, it is the fourth most mutated gene in NMIBC and the most mutated gene in MIBC, with a mutation density that doubles that of the rest of the genes. Thus, it appears that TP53 is a driver in both bladder cancer subtypes but has a greater weight in the evolution of MIBC. Conversely, RBM10 is the third most frequently mutated gene in the normal urothelium, but it appears with a low number of mutations across bladder tumors in relative terms (Extended Data Fig. 9a). These differences in the relative frequency of mutations in these genes across normal urothelium and bladder cancer samples point at potential differences in their roles in the process of tumorigenesis in this tissue. Genes with lower relative frequency of mutations across tumors may play a more preponderant role in early clonal expansions, while those more frequently mutated in relative terms may appear more involved in later steps of tumorigenesis. For example, in esophagus, the discordant relative frequency of NOTCH1 and NOTCH2 mutations

in normal esophagus and esophageal cancer has raised the notion that these may actually be protective in the face of malignization[52].

Sex bias in bladder cancer

We asked the question of whether the sex difference observed in the clonal selection of mutations in RBM10, CDKN1A and ARID1A also occurs in bladder tumors. To count with a large enough cohort, we used the exome data of 2,965 bladder urothelial carcinomas (BLCA) obtained from the GENIE project[53] (Supplementary Figure 54). We classified tumors in mutated and not mutated for each gene analyzed in the normal bladder study and ran univariate logistic regressions with sex as the dependent variable and the gene mutational status as the independent variables (with the exception of FOXQ1, which does not appear mutated in this cohort). For those genes with a significant association in the univariate analysis we ran a multivariate regression to account for the confounding effect of age. These analyses demonstrated that males have a significantly higher frequency of bladder tumors with mutations in RBM10 and CDKN1A than females (Extended Data Fig. 8c,d and Supplementary Figure 54).

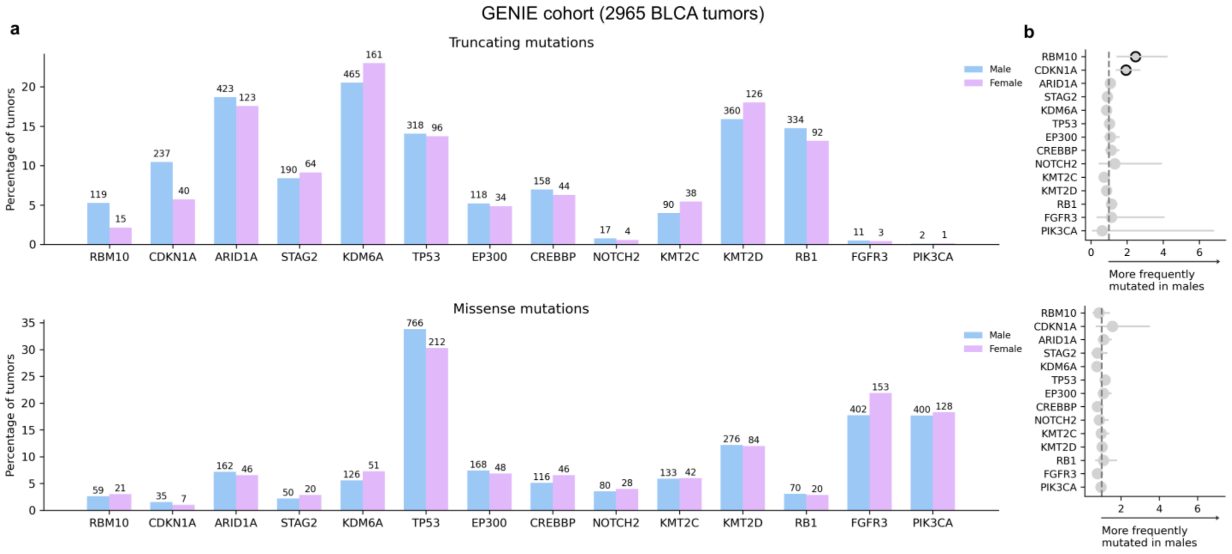

Supplementary Figure 54. Analysis of sex bias in a cohort of bladder tumors.

a) Percentage of mutated tumors for truncating (above) and missense (below) mutations across males and females for the 15 coding genes sequenced in the normal bladder study. FOXQ1 is not shown because no mutation was identified in the cohort. The absolute number of tumors belonging to each category is shown on top of each bar.

b) Association of sex with the presence of truncating (above) and missense (below) mutations in 14 of the genes sequenced in the normal bladder study. The plots show the effect size and confidence intervals of univariate logistic regressions for all genes except RBM10 and CDKN1A truncating, for which the results of multivariate regressions accounting for age are shown (q-value < 0.05 indicated with a dark outer circumference).

Supplementary Figure 55 details the association of RBM10 and CDKN1A with sex in this cohort of bladder carcinomas. For both genes, the fraction of tumors from males with mutations is higher than its counterpart of tumors from females, independently of the age of the patients.

in

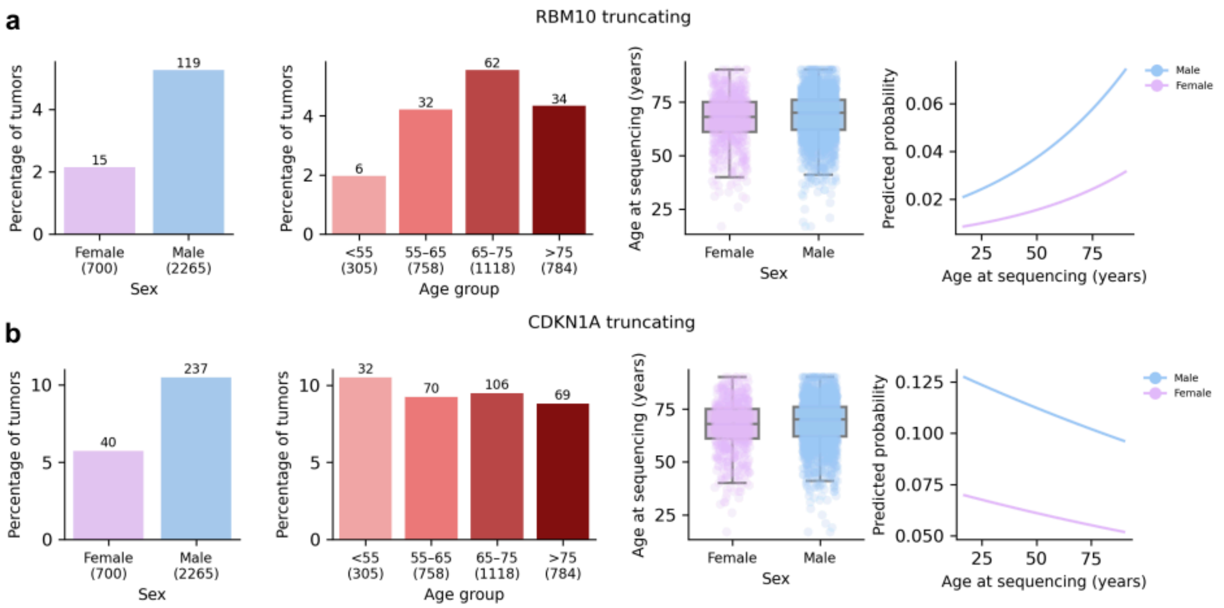

Supplementary Figure 55. Details of the sex bias of RBM10 (a) and CDKN1A (b) in a cohort of bladder tumors.

In both panels, from left to right, comparison of the frequency of tumors with truncating mutations in RBM10 (a) or CDKN1A (b) across males and females, comparison of the frequency of tumors with truncating mutations in RBM10 (a) or CDKN1A (b) across patients in different age groups, distribution of the age of male and female patients at the moment of sequencing of their tumors, probability of truncating mutations in RBM10 (a) or CDKN1A (b) in tumors from males or females depending on their age. In the first and second panel from the left, the total number of tumors belonging to each group is indicated below the x axis ticks. The specific number of tumors belonging to the mutated subset per group is shown on top of each bar.

TERT activating mutations and smoking in tumors

We next asked if activating mutations in the TERT promoter across bladder tumors are associated with smoking, as observed across normal urothelium samples. Leveraging data from 1011 bladder carcinomas sequenced using the MSK-IMPACT panel (which includes the TERT promoter), we found no significant association between TERT promoter mutations and a history of smoking, although we observed a slight increase in the frequency of TERT promoter mutations across smokers (Supplementary Figure 56)[54]. It is important to bear in mind that TERT promoter mutations are very frequent (close to 70% in this cohort) across bladder tumors, irrespective of the smoking status of patients. Importantly, a significant association was found between age and the presence of TERT promoter mutations across these tumors.

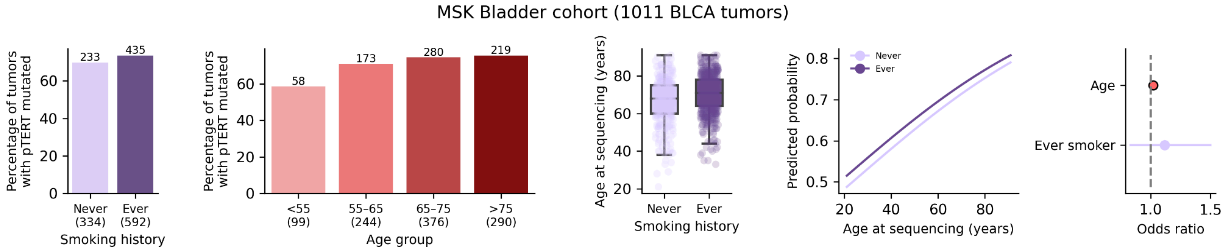

Supplementary Figure 56. TERT promoter mutated tumors association with smoking history and age in a bladder cancer cohort.

From left to right, comparison of the frequency of tumors with TERT promoter mutations across ever smokers and never smokers, comparison of the frequency of tumors with TERT promoter mutations across patients in different age groups, distribution of the age of ever smokers and never smokers at the moment of sequencing of their tumors, probability of TERT promoter mutation in tumors from ever smokers and never smokers depending on their age, and multivariate regression analysis of TERT promoter mutations on the age and smoking status of patients. In the first and second panel from the left, the total number of tumors belonging to each group is indicated below the x axis ticks. The specific number of tumors belonging to the pTERT mutated subset per group is shown on top of each bar.

We repeated this analysis in a cohort of lung tumors, since smoking is the main risk factor for lung cancer (MSK-CHORD)[55]. In this case, we identified a significant association of the presence of TERT promoter mutations with the smoking status of patients. In contrast to bladder cancer, where pTERT mutations are found in 70-80% of tumors[2], pTERT mutations are only found in a small percentage of lung cancers (<5%). However, those with pTERT mutation tend to be smokers and also of older age, as observed for bladder cancer (Supplementary Figure 57).

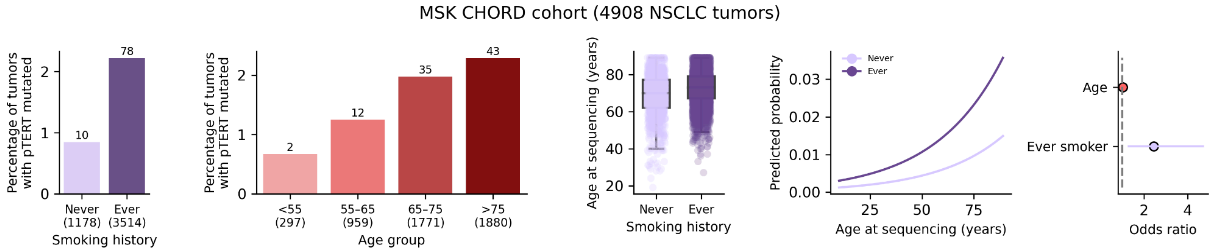

Supplementary Figure 57. TERT promoter mutated tumors association with smoking history and age in a lung cancer cohort.

From left to right, comparison of the frequency of tumors with TERT promoter mutations across ever smokers and never smokers, comparison of the frequency of tumors with TERT promoter mutations across patients in different age groups, distribution of the age of ever smokers and never smokers at the moment of sequencing of their tumors, probability of TERT promoter mutation in tumors from ever smokers and never smokers depending on their age, multivariate regression analysis of TERT promoter mutations on the age and smoking status of patients. In the first and second panel from the left, the total

number of tumors belonging to each group is indicated below the x axis ticks. The specific number of tumors belonging to the pTERT mutated subset per group is shown on top of each bar.

Implications for bladder cancer detection and risk prediction using urine molecular testing

The findings from this study indicate that caution must be taken when designing and interpreting tests to identify bladder cancer based on mutations found in urine. While this study did not investigate mutations in urine, urothelial cells are naturally shed into urine. We have demonstrated that normal urothelial cells carry common bladder cancer mutations in individuals without bladder cancer. If disseminated in urine, these mutant cells could lead to false positives in high-sensitive tests for mutation detection.

Conversely, however, while diagnosis and early detection of cancer based on mutation detection in urine or other non-invasive samples might be compromised by the high prevalence of cancer mutations in normal tissue, the characterization of the mutational landscape of such biopsies might harbor value for cancer risk prediction. Our results indicate that common cancer risk factors such as age, sex, and smoking affect the clonal landscape of the bladder epithelium and similar results have been reported in oral epithelium collected using buccal swabs[56]. Thus, while somatic evolution challenges the specificity of cancer mutations to detect cancer, it opens the possibility of using the patterns of clonal expansion to molecularly assess cancer risk.

Supplementary Note 12: Analysis of saturation mutagenesis

Abstract
We explored the kinetic of natural saturation mutagenesis, that is, the fraction of sites in a gene covered by mutations depending on the number of haploid genomes sequenced. This Supplementary Note describes this exploration under the assumption of neutral mutagenesis, that is, complete lack of selection on mutations, which is inferred from synonymous mutations. We call this the theoretical kinetic of natural saturation mutagenesis. We also computed an observed kinetic of natural saturation mutagenesis using down-sampling of the haploid genomes sequenced in this study. We hypothesize that the presence of selection in the normal urothelium (positive and negative) will make the observed kinetic of natural saturation mutagenesis different from the theoretical. Positive selection might favor the observation of more mutations for a given depth, especially if it affects a large number of residues, whereas negative selection will decrease the proportion of observed mutant residues since some mutations might be incompatible with cell survival.

Purpose
The large number of somatic mutations revealed within and across samples in our study suggests that observing all possible mutations (saturation mutagenesis) is plausible by adding more depth and samples. How much depth per site would be required to observe mutations in all sites? How is the dependency between proportion of mutated sites and depth? How does this compare with the empirical proportion of mutated sites if we take as reference the observed reads?

Throughout this note we will distinguish two different notions of site: 1) genomic site, i.e., nucleotide position in the genome, 2) residue site, i.e., specific amino acid position corresponding to the gene’s protein product.

Theoretical kinetic of natural saturation mutagenesis
We aimed to compute the expected proportion of mutated sites as a function of the sequencing depth. For this quantification, we take as reference the neutral mutation densities inferred across sites based on the number of synonymous mutations observed for each gene across the entire cohort of samples (Supplementary Note 6).

Assuming neutral selection, each time that an instance of a site S is sequenced (sequenced read) there is a probability p(S) to observe a non-synonymous mutation: we can estimate p(S) from our neutral mutagenesis estimates per sequenced read (Supplementary Note 6). With this information per site, we can then compute the probability to observe a mutation in S given a certain number of draws N (representing the number of duplex reads mapping to S, i.e., the duplex depth at S).

If p(S) is the probability to call a mutation in S when a sequencing duplex read overlaps S, given a certain number of draws N, the probability to observe a mutation at least once is

$$\psi(S, N) = 1 - (1 - p(S))^N$$

which can be approximated, assuming p(S) = 1/m for a sufficiently large integer m, by the following exponential expression

$$\psi(S, N) = 1 - (1 - 1/m)^N \approx 1 - e^{-N/m} = 1 - e^{-Np(S)}.$$

Assuming a collection of sites with neutral mutation density p(S) per sequenced read and a uniform depth of N, we can compute the expected number of mutated sites as:

$$U = \sum_S \psi(S, N) = \sum_S 1 - e^{-Np(S)} = n - \sum_S e^{-Np(S)},$$

where n is the total number of mutable sites.

This calculation was conducted both at the level of genomic sites and protein residues. For the residue-wise calculation we summed up the probabilities that any nucleotide in the respective codon would undergo a non-synonymous mutation and applied the method described.

The theoretical curves obtained by plotting the expected number of mutated sites against the sequencing depth indicated that a depth between 10^6 and 10^7 would enable to observe all possible residue mutations for all tested genes assuming absence of selection and the same aggregated mutation density as the samples in the study.

Observed kinetic of natural saturation mutagenesis

We next wanted to compare the theoretical expectation against the empirical proportion of mutated sites as we downsample reads from the observed. For a specified subsampling rate $\rho \in [0, 1]$ and for each sequenced position where a mutation has been observed, we aimed to subsample $N = [\rho D]$ reads with replacement from the total set of reads mapping to the site. By doing this across sites, we can compute the proportion of sites with at least one mutation.

For a site that has n alternate allele reads, the probability P to get at least one alternate allele when drawing k times without replacement from the set of N reads is:

$$P(N, n) = 1 - \prod_{i=0}^{k-1} \frac{N-n-i}{N-i}$$

Therefore, we can realize the sought subsampling experiment by randomly drawing a Bernoulli distribution with probability P(N(S),n(S)) as in the equation above, where N(S) and n(S) denote

the total depth and alternate depth at site S, respectively. Adding the result across all sites would give us the total sites mutated, hence the proportion of sites mutated. We did this experiment for a grid of subsampling rates $\rho \in [0.01, 0.9]$ with 20 uniform steps in log10 scale and across R=100 replicates, both for genomic sites and protein residues. For the residue-wise calculation, we simply binned the results obtained per genomic position within each codon: if at least one nucleotide of the codon undergoes a non-synonymous mutation, we deem the residue mutated at least once.

For each gene, the proportion of mutated residues obtained in this downsampling experiment was compared against the theoretical kinetic of natural saturation mutagenesis derived from the mutation probabilities assuming neutral evolution (Supplementary Note 6). We observed than in several genes (i.e. TP53, KDM6A, RBM10) the proportion of mutated residues observed was higher than expected due to a large number of sites with positive selection whereas for FGFR3 it was lower than expected due to overall strong negative selection (Fig. 3c, Fig. 5b, Extended Data Fig. 9c, Supplementary Fig. 1 and 2).

Conclusion

The large number of mutations identified for each gene with ultradeep duplex sequencing, specifically the number of synonymous mutations, enabled to estimate the theoretical aggregated duplex depth (i.e. haploid genomes sequenced) required to eventually identify all possible mutations in the absence of selection (theoretical kinetic of saturation mutagenesis). We estimated that, in theory, given the mutation density of the samples in this study, an aggregated depth of 10^7 would enable us to identify all possible mutations in most genes. However, the normal urothelium, as most human tissues, is affected by the dynamic forces of selection (positive and negative), which ultimately determines which and how many mutations will be observed. Mutations that are constitutively deleterious for the cell will never be observed whereas others might be observed or not depending on their phenotypic effect on the cell at a given time and context. Probing more patients and samples with ultradeep duplex sequencing will enable us to add even greater resolution to the rich mutational landscapes already generated in this cohort (Supplementary Figures 1 and 2). This analysis proves the value of natural saturation mutagenesis for widescreen study of mutational functionality in vivo across genes and tissues.

Supplementary References

1. Sudlow, C. *et al.* UK Biobank: An Open Access Resource for Identifying the Causes of a Wide Range of Complex Diseases of Middle and Old Age. *PLOS Med.* **12**, e1001779 (2015).
2. Dyrskjøt, L. *et al.* Bladder cancer. *Nat. Rev. Dis. Primer* **9**, 1–21 (2023).
3. Jubber, I. *et al.* Epidemiology of Bladder Cancer in 2023: A Systematic Review of Risk Factors. *Eur. Urol.* **84**, 176–190 (2023).
4. Cumberbatch, M. G. K. *et al.* Epidemiology of Bladder Cancer: A Systematic Review and Contemporary Update of Risk Factors in 2018. *Eur. Urol.* **74**, 784–795 (2018).
5. Kar, S. P. *et al.* Genome-wide analyses of 200,453 individuals yield new insights into the causes and consequences of clonal hematopoiesis. *Nat. Genet.* **54**, 1155–1166 (2022).
6. Jackson, S. S. *et al.* Sex disparities in the incidence of 21 cancer types: Quantification of the contribution of risk factors. *Cancer* **128**, 3531–3540 (2022).
7. Trinder, M., Walley, K. R., Boyd, J. H. & Brunham, L. R. Causal Inference for Genetically Determined Levels of High-Density Lipoprotein Cholesterol and Risk of Infectious Disease. *Arterioscler. Thromb. Vasc. Biol.* **40**, 267–278 (2020).
8. Martínez-Jiménez, F. *et al.* A compendium of mutational cancer driver genes. *Nat. Rev. Cancer* **20**, 555–572 (2020).
9. Campbell, P. J. *et al.* Pan-cancer analysis of whole genomes. *Nature* **578**, 82–93 (2020).
10. Sabarinathan, R. *et al.* The whole-genome panorama of cancer drivers. *bioRxiv* (2017) doi:10.1101/190330.
11. Abascal, F. *et al.* Somatic mutation landscapes at single-molecule resolution. *Nature* **593**, 405–410 (2021).
12. Li, H. & Durbin, R. Fast and accurate short read alignment with Burrows–Wheeler transform. *Bioinformatics* **25**, 1754–1760 (2009).
13. Miller, C. A. *et al.* Failure to Detect Mutations in U2AF1 due to Changes in the GRCh38 Reference Sequence. *J. Mol. Diagn.* **24**, 219–223 (2022).
14. Arnedo-Pac, C., Muiños, F., Gonzalez-Perez, A. & Lopez-Bigas, N. Hotspot propensity across mutational processes. *Mol. Syst. Biol.* **20**, 6–27 (2024).
15. Quinlan, A. R. & Hall, I. M. BEDTools: a flexible suite of utilities for comparing genomic features. *Bioinformatics* **26**, 841–842 (2010).
16. Hoang, M. L. *et al.* Genome-wide quantification of rare somatic mutations in normal human tissues using massively parallel sequencing. *Proc. Natl. Acad. Sci.* **113**, 9846–9851 (2016).
17. Mohammad, A., Laboulaye, M. A., Shenhar, C. & Dobberfuhl, A. D. Mechanisms of oxidative stress in interstitial cystitis/bladder pain syndrome. *Nat. Rev. Urol.* **21**, 433–449 (2024).
18. Lawson, A. R. J. *et al.* Extensive heterogeneity in somatic mutation and selection in the human bladder. *Science* **370**, 75–82 (2020).
19. Osorio, F. G. *et al.* Somatic Mutations Reveal Lineage Relationships and Age-Related Mutagenesis in Human Hematopoiesis. *Cell Rep.* **25**, 2308-2316.e4 (2018).
20. Machado, H. E. *et al.* Diverse mutational landscapes in human lymphocytes. *Nature* **608**, 724–732 (2022).
21. Tate, J. G. *et al.* COSMIC: the Catalogue Of Somatic Mutations In Cancer. *Nucleic Acids Res.* **47**, D941–D947 (2019).
22. Alexandrov, L. B. *et al.* Signatures of mutational processes in human cancer. *Nature* **500**, 415–21 (2013).
23. Alexandrov, L. B. *et al.* The repertoire of mutational signatures in human cancer. *Nature* **578**, 94–101 (2020).
24. Lee-Six, H. *et al.* The landscape of somatic mutation in normal colorectal epithelial cells. *Nature* **574**, 532–537 (2019).
25. Liu, M., Wu, Y., Jiang, N., Boot, A. & Rozen, S. G. mSigHdp: hierarchical Dirichlet process mixture modeling for mutational signature discovery. *NAR Genomics Bioinforma.* **5**, lqad005 (2023).
26. Bergstrom, E. N. *et al.* SigProfilerMatrixGenerator: a tool for visualizing and exploring patterns of small mutational events. *BMC Genomics* **20**, 685 (2019).
27. Islam, S. M. A. *et al.* Uncovering novel mutational signatures by de novo extraction with SigProfilerExtractor. 2020.12.13.422570 Preprint at https://doi.org/10.1101/2020.12.13.422570 (2022).
28. Alexandrov, L. B. *et al.* Clock-like mutational processes in human somatic cells. *Nat. Genet.* **47**, 1402–1407 (2015).
29. Mularoni, L. *et al.* OncodriveFML: a general framework to identify coding and non-coding regions with cancer driver mutations. (2016) doi:10.1186/s13059-016-0994-0.
30. Pellegrini, Stefano, Muinos, F., Lopez-Bigas, N. & Gonzalez-Perez, Abel. Oncodrive3D: Fast and accurate detection of structural clusters of mutations under positive selection. *Prep.*
31. Harrison, P. W. *et al.* Ensembl 2024. *Nucleic Acids Res.* **52**, D891–D899 (2024).

32. McLaren, W. *et al.* The Ensembl Variant Effect Predictor. *Genome Biol.* **17**, 122 (2016).
33. Morales, J. *et al.* A joint NCBI and EMBL-EBI transcript set for clinical genomics and research. *Nature* **604**, 310–315 (2022).
34. Schuster-Böckler, B. & Lehner, B. Chromatin organization is a major influence on regional mutation rates in human cancer cells. *Nature* **488**, 504–507 (2012).
35. Lawrence, M. S. *et al.* Mutational heterogeneity in cancer and the search for new cancer-associated genes. *Nature* **499**, 214–218 (2013).
36. Gonzalez-Perez, A., Sabarinathan, R. & Lopez-Bigas, N. Local Determinants of the Mutational Landscape of the Human Genome. *Cell* **177**, 101–114 (2019).
37. Martincorena, I. *et al.* Universal Patterns of Selection in Cancer and Somatic Tissues. *Cell* **171**, 1029-1041.e21 (2017).
38. Kircher, M. *et al.* A general framework for estimating the relative pathogenicity of human genetic variants. *Nat. Genet.* **46**, 310–5 (2014).
39. Jumper, J. *et al.* Highly accurate protein structure prediction with AlphaFold. *Nature* **596**, 583–589 (2021).
40. Varadi, M. *et al.* AlphaFold Protein Structure Database: massively expanding the structural coverage of protein-sequence space with high-accuracy models. *Nucleic Acids Res.* **50**, D439–D444 (2021).
41. Eilbeck, K. *et al.* The Sequence Ontology: a tool for the unification of genome annotations. *Genome Biol.* **6**, R44 (2005).
42. Mcdonald, J. H. HANDBOOK OF BIOLOGICAL STATISTICS.
43. Priestley, P. *et al.* Pan-cancer whole-genome analyses of metastatic solid tumours. *Nature* **575**, 210–216 (2019).
44. Martínez-Jiménez, F. *et al.* Pan-cancer whole-genome comparison of primary and metastatic solid tumours. *Nature* **618**, 333–341 (2023).
45. Rheinbay, E. *et al.* Analyses of non-coding somatic drivers in 2,658 cancer whole genomes. *Nature* **578**, 102–111 (2020).
46. Hafner, C. *et al.* Evidence for oligoclonality and tumor spread by intraluminal seeding in multifocal urothelial carcinomas of the upper and lower urinary tract. *Oncogene* **20**, 4910–4915 (2001).
47. Pich, O., Reyes-Salazar, I., Gonzalez-Perez, A. & Lopez-Bigas, N. *Discovering the Drivers of Clonal Hematopoiesis*. http://biorxiv.org/lookup/doi/10.1101/2020.10.22.350140 (2020) doi:10.1101/2020.10.22.350140.
48. Bolton, K. L. *et al.* Cancer therapy shapes the fitness landscape of clonal hematopoiesis. *Nat. Genet.* **52**, 1219–1226 (2020).
49. Hagiwara, K. *et al.* Dynamics of Age- versus Therapy-Related Clonal Hematopoiesis in Long-term Survivors of Pediatric Cancer. *Cancer Discov.* **13**, 844–857 (2023).
50. Hurst, C. D. *et al.* Genomic Subtypes of Non-invasive Bladder Cancer with Distinct Metabolic Profile and Female Gender Bias in *KDM6A* Mutation Frequency. *Cancer Cell* **32**, 701-715.e7 (2017).
51. Berletch, J. B., Yang, F., Xu, J., Carrel, L. & Disteche, C. M. Genes that escape from X inactivation. *Hum. Genet.* **130**, 237–245 (2011).
52. Martincorena, I. *et al.* Somatic mutant clones colonize the human esophagus with age. *Science* **362**, 911–917 (2018).
53. AACR Project GENIE: Powering Precision Medicine through an International Consortium. *Cancer Discov.* **7**, 818–831 (2017).
54. Clinton, T. N. *et al.* Genomic heterogeneity as a barrier to precision oncology in urothelial cancer. *Cell Rep.* **41**, 111859 (2022).
55. Jee, J. *et al.* Automated real-world data integration improves cancer outcome prediction. *Nature* **636**, 728–736 (2024).
56. Yokoyama, A. *et al.* Somatic mosaicism in the buccal mucosa reflects lifestyle and germline risk factors for esophageal squamous cell carcinoma. *Sci. Transl. Med.* **17**, eadq6740 (2025).
