# Supplementary material for: Sex and smoking bias in the selection of somatic mutations in human bladder
Source: Nature. 2025 Oct 8;647(8089):436–44. doi: 10.1038/s41586-025-09521-x (PMC12611770; doi:10.1038/s41586-025-09521-x)
Supplement: Supplementary file 2 — Reporting Summary [file 41586_2025_9521_MOESM2_ESM.pdf]

nature portfolio

Corresponding author(s): Nuria Lopez-Bigas and Rosa Ana Risques

Last updated by author(s): Jul 6, 2025

Reporting Summary

Nature Portfolio wishes to improve the reproducibility of the work that we publish. This form provides structure for consistency and transparency in reporting. For further information on Nature Portfolio policies, see our Editorial Policies and the Editorial Policy Checklist.

Statistics

For all statistical analyses, confirm that the following items are present in the figure legend, table legend, main text, or Methods section.

n/a	Confirmed	
☐	☒	The exact sample size (*n*) for each experimental group/condition, given as a discrete number and unit of measurement
☒	☐	A statement on whether measurements were taken from distinct samples or whether the same sample was measured repeatedly
☐	☒	The statistical test(s) used AND whether they are one- or two-sided<br>*Only common tests should be described solely by name; describe more complex techniques in the Methods section.*
☐	☒	A description of all covariates tested
☐	☒	A description of any assumptions or corrections, such as tests of normality and adjustment for multiple comparisons
☐	☒	A full description of the statistical parameters including central tendency (e.g. means) or other basic estimates (e.g. regression coefficient) AND variation (e.g. standard deviation) or associated estimates of uncertainty (e.g. confidence intervals)
☐	☒	For null hypothesis testing, the test statistic (e.g. *F*, *t*, *r*) with confidence intervals, effect sizes, degrees of freedom and *P* value noted<br>*Give P values as exact values whenever suitable.*
☒	☐	For Bayesian analysis, information on the choice of priors and Markov chain Monte Carlo settings
☒	☐	For hierarchical and complex designs, identification of the appropriate level for tests and full reporting of outcomes
☐	☒	Estimates of effect sizes (e.g. Cohen's *d*, Pearson's *r*), indicating how they were calculated

Our web collection on statistics for biologists contains articles on many of the points above.

Software and code

Policy information about availability of computer code

Data collection

DNA extracted from 79 samples obatained by brusing one or two sites (2-3cm2) of the bladder of 45 donors upon autopsy was sequenced using a duplex DNA sequencing technology with commercially available kits (TwinStrand Biosciences, Seattle, WA). Sample processing and the experimental protocol used in the duplex sequencing are described in detail in the Methods section and in the Supplementary Note 2.

DNA extracted from 3 cord blood samples was also obtained and sequenced using the same DNA sequencing technology. This is described in the Supplementary Note 4.

Clinical data for the 45 donors, including age, sex, BMI, tobacco smoking history, alcohol use, prior cancer, and chemotherapy exposure was also obtained.

Mutations identified across 622 muscle invasive and 105 non-muscle invasive bladder cancer (MIBC and NMBIC, respectively) cohorts were downloaded from cBioPortal together with the clinical data of the combined study. From the MIBC BGI cohort only samples labeled as invasive were considered MIBC and added to the MIBC dataset. Mutations identified in an additional cohort of 79 NMIBCs were obtained. from the literature and included as part of the NMIBC dataset. Mutations identified across 33,218 tumors (892 bladder tumors) in intOGen were downloaded from intogen.org. These mutations were used to obtain the total number of mutations observed in each gene, their distribution along the sequence of the genes in the study, and the percentage of sites affected by different numbers of mutations. The same data of 109,017 tumors (3,909 bladder tumors) were obtained from the GENIE project to calculate the frequency of mutations in each of the genes and the TERT promoter. Mutations in the TERT promoter were also obtained from two cohorts of tumors (included in intOGen) sequenced at the whole genome level (Hartwig Medical Foundation: N=5,582 and PCAWG: N=2,554). The classification (and score) of all possible mutations in TP53 into drivers and passengers via in silico saturation mutagenesis was obtained from boostDM (intogen.org/boostDM). Details of analyses involving tumor mutations appear in Supplementary Note 11.

	Structural models for all proteins used to run Oncodrive3D were obtained from the AlphaFold database (AlphaFold 2 v.4), as were the structural features of proteins. Solvent accessibility and secondary structure information were extracted from the AlphaFold-predicted PDB structures using PDB_Tool (https://github.com/realbigws/PDB_Tool).<br><br>We obtained two saturation mutagenesis experiments estimating the functional impact of mutations in the TP53 DNA binding domain and along the sequence of the TERT promoter from their original publications, cited in ther Methods section.
Data analysis	Somatic mutations from the raw sequencing data were called using a computational pipeline (deepUMIcaller) implemented by us in Nextflow on the basis of an early version of nf-core/fastquorum64 pipeline, which implements the fgbio Best Practices FASTQ to Consensus Pipeline (https://github.com/fulcrumgenomics/fgbio/blob/main/docs/best-practice-consensus-pipeline.md) and downstream variant calling based on VarDictJava (https://github.com/AstraZeneca-NGS/VarDictJava). A series of filters to discard potential artifacts are included in the pipeline. The code implementing deepUMIcaller is publicly available at (github.com/bbglab/deepumicaller) upon publication. For a detailed description of the pipeline, see Supplementary Note 3.<br><br>A second pipeline, deepCSA was implemented by us also in Nextflow to automate all downstream analyses caried out on somatic mutations. This second pipeline is available at https://github.com/bbglab/deepCSA.<br><br>The analyses implemented in deepCSA include:<br><br>-Extraction of mutational signatures de novo using Bayesian hierarchical Dirichlet process using HDP_sigExtraction pipeline (https://github.com/McGranahanLab/HDP_sigExtraction) based on the R-package hdp developed by Nicola Roberts (https://github.com/nicolaroberts/hdp)9 and SigProfilerExtractor (v.1.2.1) (https://github.com/AlexandrovLab/SigProfilerExtractor).<br>-Four methods to compute positive selection on the mutations observed across genes are employed in this article. One of them (omega), a dN/dS approach to assess the strength of selection on the mutational pattern of genes, was developed de novo for this study and is described at length in Supplementary Note 6 (https://github.com/bbglab/omega). Two others, OncodriveFML and Oncodrive3D, which compute the deviation in the average functional impact and clustering in the three-dimensional structure of proteins, respectively, from those expected under neutrality, had been developed previously, and were adapted here to work on duplex sequencing data. These are also described in Supplementary Note 6. A fourth method, assessing the relative enrichment for frameshift indels observed across genes was also developed de novo for this study and is thoroughly described in Supplementary Note 6.<br>-Mixed-effects linear models were implemented to test the association between clinical variables and the clonal structure of samples, represented by the magnitude of positive selection on mutations of different genes. Associations with FDR below 0.2 were deemed significant. We also carried a binomial test to rule out a spurious dependence of the mutation density on group differences in terms of the sequencing depth. All statistical details of these models and tests carried out to assess their power are explained in Supplementary Notes 9 and 10.<br><br>We also designed a method to compute the natural saturation mutagenesis kinetics of genes across samples, described in details in Methods and Supplementary Note 12.

For manuscripts utilizing custom algorithms or software that are central to the research but not yet described in published literature, software must be made available to editors and reviewers. We strongly encourage code deposition in a community repository (e.g. GitHub). See the Nature Portfolio guidelines for submitting code & software for further information.

Data

Policy information about availability of data

All manuscripts must include a data availability statement. This statement should provide the following information, where applicable:

- Accession codes, unique identifiers, or web links for publicly available datasets
- A description of any restrictions on data availability
- For clinical datasets or third party data, please ensure that the statement adheres to our policy

Raw sequencing data for this study were deposited in dbGaP under accession number phs004105.v1.p1. The set of mutations used in all analyses presented in the paper is available at https://doi.org/10.5281/zenodo.15836679. Executing the code provided in the third repository mentioned below, all figures in the paper can be reproduced. Reference mutational signatures were obtained from https://cancer.sanger.ac.uk/signatures/sbs/. Tumor mutations were obtained through cBioPortal (datasets from references16,17,74 at https://www.cbioportal.org/), intogen (intogen.org) and the GENIE synapse data portal (https://genie.synapse.org/) as described in Methods. Protein structural models for the entire human proteome were obtained from the AlphaFold database (https://alphafold.ebi.ac.uk/). The results of two experimental saturation mutagenesis studies on TP53 and the TERT promoter were obtained from references51,55, respectively.

Research involving human participants, their data, or biological material

Policy information about studies with human participants or human data. See also policy information about sex, gender (identity/presentation), and sexual orientation and race, ethnicity and racism.

Reporting on sex and gender	One of the most salient results of the study is the difference in the clonal landscape of the urothelium between males and females.
Reporting on race, ethnicity, or other socially relevant groupings	N/A
Population characteristics	Described in Supplementary Note 2 and Extended Data Table 1

Recruitment	Samples were obtained from deceased individuals without known bladder pathology and no history of bladder cancer upon autopsy at the University of Washington after obtaining consent from next-of-kin.
Ethics oversight	The study was deemed not human subjects by the Institutional Review Board at University of Washington (STUDY00016707) because research involving deceased individuals is not human subjects research by US Federal guidelines (45CFR46).

Note that full information on the approval of the study protocol must also be provided in the manuscript.

Field-specific reporting

Please select the one below that is the best fit for your research. If you are not sure, read the appropriate sections before making your selection.

☒ Life sciences ☐ Behavioural & social sciences ☐ Ecological, evolutionary & environmental sciences

For a reference copy of the document with all sections, see nature.com/documents/nr-reporting-summary-flat.pdf

Life sciences study design

All studies must disclose on these points even when the disclosure is negative.

Sample size	79 samples from 45 deceased individuals (53 before exclusions). No prior sample size calculation was carried out; however, we conclusively demonstrate (Supplementary Note 10) that the sample size of the cohort provides the statistical power required to detect the associations reported in the manuscript.
Data exclusions	Samples from three individuals with active or chronic inflammation and four individuals with insufficient DNA were discarded. The two samples of a separate individual with cystitis and evidence of a large proportion of artifacts among mutations were also discarded.
Replication	All samples in the cohort were used in discovery. Nevertheless, controls with subsets of the samples were carried out.
Randomization	All samples in the cohort were used in all main analyses. Nevertheless, in particular analyses in the manuscript, such as the determination of the conservation of positive selection across dome and trigone samples, and the evaluation of the impact of errors on the analyses, randomization of samples or sets of mutations was carried out.
Blinding	All samples in the cohort were used in all main analyses, precluding the need for blinding.

Reporting for specific materials, systems and methods

We require information from authors about some types of materials, experimental systems and methods used in many studies. Here, indicate whether each material, system or method listed is relevant to your study. If you are not sure if a list item applies to your research, read the appropriate section before selecting a response.

Materials & experimental systems

n/a	Involved in the study	
☐	☒	Antibodies
☒	☐	Eukaryotic cell lines
☒	☐	Palaeontology and archaeology
☒	☐	Animals and other organisms
☒	☐	Clinical data
☒	☐	Dual use research of concern
☒	☐	Plants

Methods

n/a	Involved in the study	
☒	☐	ChIP-seq
☒	☐	Flow cytometry
☒	☐	MRI-based neuroimaging

Antibodies

Antibodies used	CK7. Supplier: Agilent (Dako). Clone name: OV-TL 12/30. Catalog number: M7018. Lot number: 41491921.<br>Uroplakin. Supplier: Biocare Medical. Clone name: BC21. Catalog number: AP13051 AA. Lot number: 022724A.<br>CD45. Supplier: Agilent (Dako). Clone name: 2B11&PD7/26. Catalog number: IR751. Lot number: 41718528.<br>Smooth Muscle Actin. Supplier: Cell Marque. Clone name: 1A4. Catalog number: 202M-95. Lot number: 323477.
Validation	CK7. Intended use: For in vitro diagnostic use by immunohistochemistry. Species reactivity: Human. Validation: Monoclonal Mouse Anti-Human Cytokeratin 7 consistently labels a large number of simple-, complex- and transitional epithelia, including all cell layers of urothelium (transitional epithelium) (1).<br><br>Uroplakin. Intended use: For in vitro diagnostic use by immunohistochemistry. Species reactivity: Human. Validation: UPII and UPIII may be found in the urothelial surface membrane of human renal pelvis, ureter, bladder and urethra. UPII and UPIII have also been identified as sensitive and highly specific markers for urothelial carcinoma (2-5). New mouse monoclonal antibodies to UPII, clone

BC21, and UPIII, clone BC17, have been developed and evaluated for sensitivity in urothelial carcinoma and specificity versus normal and neoplastic tissues (6).

CD45. Intended use: For in vitro diagnostic use by immunohistochemistry. Species reactivity: Human. Validation: Anti-CD45 is a mixture of two monoclonal antibodies, clones 2B11 and PD7/26, directed against different epitopes. Clone 2B11 was clustered as anti-CD45 at the Third International Workshop and Conference on Human Leucocyte Differentiation Antigens and reacts with all the known isotypes of the CD45 family (7). Clone PD7/26 was clustered as anti-CD45RB at the Fifth International Workshop and Conference on Human Leucocyte Differentiation Antigens (8).

Smooth Muscle Actin. Intended use: For in vitro diagnostic use by immunohistochemistry. Species reactivity: Human. Validation: Anti-smooth muscle actin immunohistochemical reactivity is seen in smooth muscle cells, myofibroblasts and myoepithelial cells (9-11). The antibody was validated by Cell Marque in a collection of normal tissues and demonstrated positive staining for smooth muscle and myoepithelium.

References
1. van Niekerk CC, Jap PHK, Ramaekers FCS, van de Molengraft F, Poels LG. Immunohistochemical demonstration of keratin 7 in routinely fixed paraffin-embedded human tissues. J Pathol 1991;165:145-52
2. Moll R, et al. Uroplakins, specific membrane proteins of urothelial umbrella cells, as histological markers of metastatic transitional cell carcinomas. Am J Pathol. 1995 Nov; 147(5):1383-97.
3. Kaufmann O, Volmerig J, Dietel M. Uroplakin III is a highly specific and moderately sensitive immunohistochemical marker for primary and metastatic urothelial carcinomas. Am J Clin Pathol. 2000 May; 113(5):683-7.
4. Olsburgh J, et al. Uroplakin gene expression in normal human tissues and locally advanced bladder cancer. J Pathol. 2003 Jan; 199(1):41-9.
5. Huang HY, et al. Persistent uroplakin expression in advanced urothelial carcinomas: implications in urothelial tumor progression and clinical outcome. Hum Pathol. 2007 Nov; 38(11):1703-13.
6. Hoang LL, et al. A newly developed Uroplakin II antibody with increased sensitivity in urothelial carcinoma of the bladder. Arch Pathol Lab Med. 2014 Jul;138(7):943-9.
7. Cobbold S, Hale G, Waldmann H. Non-lineage, LFA-1, and leucocyte common antigens: new and previously defined clusters. In: McMichael AJ, Beverley PCL, Cobbold S, Crumpton MJ, Gilks W, Gotch FM, et al., editors. Leukocyte typing III. White cell differentiation antigens. Proceedings of the 3rd International Workshop and Conference; 1986 Sep 21-26; Oxford, England. Oxford, New York, Tokyo: Oxford University Press; 1987. p. 788-803.
8. Morimoto C. T18. CD45 cluster report. In: Schlossman SF, Boumsell L, Gilks W, Harlan JM, Kishimoto T, Morimoto C, et al., editors. Leukocyte typing V. White cell differentiation antigens. Proceedings of the 5th International Workshop and Conference; 1993 Nov 3-7; Boston, USA. Oxford, New York, Tokyo: Oxford University Press; 1995. p. 386-9.
9. Cooke PH. A filamentous cytoskeleton in vertebrate smooth muscle fibers. J Cell Biol. 1976; 68:539-56.
10. Skalli O, et al. A monoclonal antibody against alpha-smooth muscle actin: a new probe for smooth muscle differentiation. J Cell Biol. 1986; 103:2787-96.
11. Perez-Montiel MD, et al. Differential expression of smooth muscle myosin, smooth muscle actin, h-caldesmon, and calponin in the diagnosis of myofibroblastic and smooth muscle lesions of skin and soft tissue. Am J Dermatopathol. 2006; 28:105-11.

Plants

Seed stocks

Report on the source of all seed stocks or other plant material used. If applicable, state the seed stock centre and catalogue number. If plant specimens were collected from the field, describe the collection location, date and sampling procedures.

Novel plant genotypes

Describe the methods by which all novel plant genotypes were produced. This includes those generated by transgenic approaches, gene editing, chemical/radiation-based mutagenesis and hybridization. For transgenic lines, describe the transformation method, the number of independent lines analyzed and the generation upon which experiments were performed. For gene-edited lines, describe the editor used, the endogenous sequence targeted for editing, the targeting guide RNA sequence (if applicable) and how the editor was applied.

Authentication

Describe any authentication procedures for each seed stock used or novel genotype generated. Describe any experiments used to assess the effect of a mutation and, where applicable, how potential secondary effects (e.g. second site T-DNA insertions, mosiacism, off-target gene editing) were examined.
